# Phylogenetic review of tonal sound production in whales in relation to sociality

**DOI:** 10.1186/1471-2148-7-136

**Published:** 2007-08-10

**Authors:** Laura J May-Collado, Ingi Agnarsson, Douglas Wartzok

**Affiliations:** 1 Florida International University, Department of Biological Sciences, 11200 SW 8^th ^Street, Miami, FL 33199, USA; 2 Universidad de Costa Rica, Escuela de Biología, Apto. 2060 San Pedro, Costa Rica; 3 Department of Biology, University of Akron, Akron, OH 44325-3908, USA

## Abstract

**Background:**

It is widely held that in toothed whales, high frequency tonal sounds called 'whistles' evolved in association with 'sociality' because in delphinids they are used in a social context. Recently, whistles were hypothesized to be an evolutionary innovation of social dolphins (the 'dolphin hypothesis'). However, both 'whistles' and 'sociality' are broad concepts each representing a conglomerate of characters. Many non-delphinids, whether solitary or social, produce tonal sounds that share most of the acoustic characteristics of delphinid whistles. Furthermore, hypotheses of character correlation are best tested in a phylogenetic context, which has hitherto not been done. Here we summarize data from over 300 studies on cetacean tonal sounds and social structure and phylogenetically test existing hypotheses on their co-evolution.

**Results:**

Whistles are 'complex' tonal sounds of toothed whales that demark a more inclusive clade than the social dolphins. Whistles are also used by some riverine species that live in simple societies, and have been lost twice within the social delphinoids, all observations that are inconsistent with the dolphin hypothesis as stated. However, cetacean tonal sounds and sociality are intertwined: (1) increased tonal sound modulation significantly correlates with group size and social structure; (2) changes in tonal sound complexity are significantly concentrated on social branches. Also, duration and minimum frequency correlate as do group size and mean minimum frequency.

**Conclusion:**

Studying the evolutionary correlation of broad concepts, rather than that of their component characters, is fraught with difficulty, while limits of available data restrict the detail in which component character correlations can be analyzed in this case. Our results support the hypothesis that sociality influences the evolution of tonal sound complexity. The level of social and whistle complexity are correlated, suggesting that complex tonal sounds play an important role in social communication. Minimum frequency is higher in species with large groups, and correlates negatively with duration, which may reflect the increased distances over which non-social species communicate. Our findings are generally stable across a range of alternative phylogenies. Our study points to key species where future studies would be particularly valuable for enriching our understanding of the interplay of acoustic communication and sociality.

## Background

Cetacean tonal signals are broadly defined as narrowband, frequency modulated sounds [1-3]. Such sounds are produced by both baleen whales (Mysticeti) and toothed whales (Odontoceti) – sister clades containing all extant whales. They are also produced by other mammals [e.g., [4]] and thus appear primitively present in the order. Baleen whales produce sounds that have fundamental frequencies generally below 5 kHz [2,5], as do members of the sister lineage of Cetacea, the hippos [4]. In toothed whales, in contrast, these sounds most commonly range from 5–20 kHz [2], and in some species, e.g. *Delphinus delphis*, *Stenella attenuata*, *S. coeruleoalba*, *S. longirostris *[6]*Lagenorhynchus albirostris *[7], *Tursiops truncatus *[8], fundamental frequencies can go as high as 48 kHz in *Inia geoffrensis *[9]. In delphinid toothed whales these high frequency tonal sounds, especially when complex, are often referred to as 'whistles', although within the group whistle acoustic characteristics vary enormously. Several species produce both frequency modulated whistles (e.g., sine, convex, concave, upsweep, downsweep) and simple whistles that are relatively constant in frequency (e.g., *Lagenorhynchus albirostris*, [7]; *Sotalia fluviatilis *[10]; *Stenella longirostris *[11], others are limited to simple whistles (*Lipotes vexillifer*) [12] or to few frequency modulated whistles (e.g., mostly downsweep in *Inia geoffrensis*) [9]. In addition, whistle contour may be continuous or consist of a series of breaks and segments [2]. Whistles may or not contain harmonics [2]. In delphinid species like *S. longirostris *[13] and *L. albirostris *[14] whistles can contain high order-harmonics. Finally, whistle duration is very variable. For instance, in *Sousa chinensis *whistles can range from 0.01 to 1.3 seconds [15] and in *Tursiops truncatus *from 0.05 to 3.2 seconds [16]. In delphinids, whistle frequency modulation and duration varies within species in relation to geography [e.g., [10,11,16,17]], and related species differ in many whistle frequency components (e.g., maximum, minimum, end, and start frequency) [e.g., [18-22]].

Baleen whales produce a great variety of sounds, among them tonal sounds that like toothed whale 'whistles', are narrowband and frequency modulated, although typically much lower in frequency [1]. These tonal sounds can be produced in isolation or in combination with other sounds (e.g. pulsative sounds). In the Right whale (*Balaena glacialis*) these tonal sounds, again like 'whistles' in toothed whales, are used in a social context [23]. For example, in Blue whales (*Balaenoptera musculus*) tonal sounds are presumably used for long-distance communication [24], and in Right whales tonal sounds are used in combination with pulsative sounds in a sexual context [25]. However, in baleen whales, these tonal sounds are never referred to as whistles, but as 'calls', 'moans' or 'tones' [26,24-29]. Nomenclature of sounds, both in toothed and baleen whales, is confusing. As stated by Au (2000: 31) [1] in baleen whales "as with dolphins there is a lack of any standard nomenclature for describing emitted sounds", this frustrates comparison of sounds across taxa and obscures homologies. It remains unclear exactly what is a 'whistle', and if narrowband, frequency modulated tonal sounds of baleen whales and toothed whales are homologous at some level. One reason to question tonal sound homology across whales is that the sound production mechanisms of baleen whales and toothed whales are dramatically different. In baleen whales tonal sounds are thought to be laryngeal [30,31], as they are in other related mammals [e.g. [4,32]], but in toothed whales sounds are produced by a unique and complex nasal system [e.g. [33,34]]. This offers some support for the hypothesis that toothed whales 'whistles' are unique and different from (not homologous with) baleen whale tonal sounds. However, this also suggests that high frequency tonal sounds are homologous across toothed whales and such sounds in non-delphinid toothed whales should also be called whistles (contra Podos *et al*. 2002) [35]. To accommodate both possibilities we do all analyses across all whales (allowing for potential homology of tonal sounds across the order) and separately within toothed whales.

Most of the work on whistles has been done with social delphinids, where they are often referred to as "social signals" and are thought to facilitate individual recognition, group cohesion, recruitment during feeding activities, and overall communication [e.g., [1,3,36-44]]. Generalizations about the function of whistles have translated into the hypothesis that whistles evolved in concert with sociality, and that the two traits are tightly correlated [e.g., [45,35]]. Herman and Tavolga (1980) [45] suggested that the degree of gregariousness in toothed whales seemed to be related to whistle production [see also [46]]. More specifically, they proposed that species that live in small groups or are solitary tend not to whistle, whereas species that live in large groups frequently do. Recently, Podos *et al. *(2002) [35] proposed that whistles are an innovation of social delphinids; in other words that whistles are synapomorphic for a clade within Delphinidae. However, even within delphinids some social species such as *Cephalorhynchus *spp and some species of *Lagenorhynchus *do not whistle [e.g., [46,47]], which seems to contradict the dolphin hypothesis. The hypothesis was furthermore based on an assumption of the absence of whistles in river dolphins (*Inia*, *Lipotes*, *Platanista*, and *Pontoporia*), porpoises (Phocoenidae), beaked whales (ziphids) and belugas and narwhals (Monodontidae). However, we do not believe this assumption is justified. Tonal sounds from *Inia geoffrensis*, for example, have been independently recorded in several studies [9,21,22,48]. These sounds, just like in other toothed whales, have been referred to as whistles, although they are simpler and shorter in duration, and higher in frequency than the whistles of some dolphins [9]. Similar whistles have also been reported in another river dolphin *Lipotes vexillifer *[e.g., [12,49,50]] and in social non-delphinid toothed whales such as some beaked whales [51,52], and the Monodontidae, belugas and narwhals [e.g. [53-57]]. Podos *et al*. (2002) [35] concluded that the tonal sounds in these species should not be classified as 'whistles', and hence found support for the dolphin hypothesis. While we agree with Podos *et al*. that whistle structure seems different in delphinids and non-delphinid toothed whales we believe this demonstrates the basic problem of treating broad, arbitrary, concepts as single traits in evolutionary analyses. To define whistles as social sounds produced by delphinids – a priori denying homology with tonal sounds in related taxa – and then concluding that they evolved in association with sociality in Delphinidae risks circularity. In such a framework reconstructing the origin of 'whistles' on a phylogeny will simply depend on the whistle definition chosen by any given author.

**Figure 1:**
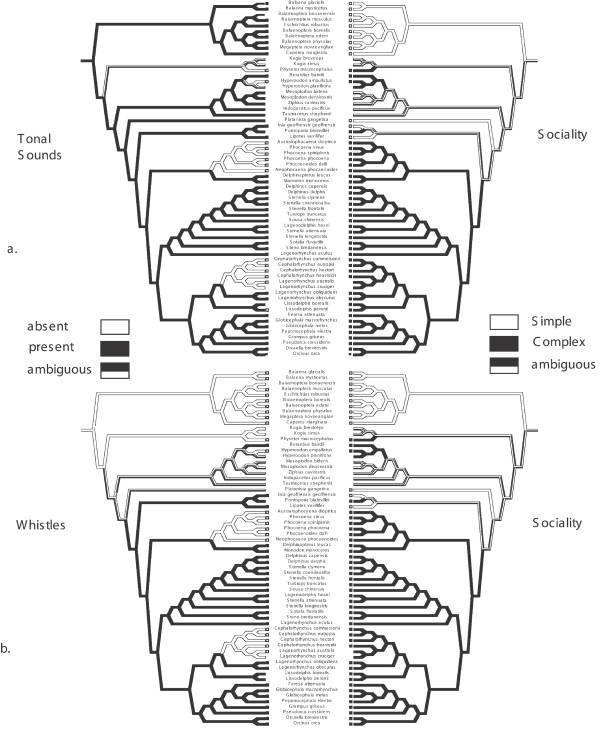
Optimizations of tonal sounds (a) and whistles (b) versus sociality using the broad concept approach [see additional file 1]. A brief glance at the black branches (indicating presence of tonal sounds/whistles and 'complex' sociality) on each side does not suggest detailed correspondence of acoustic structure with sociality. In other words whistles have a different phylogenetic distribution than does complex sociality etc, indicating that their co-evolutionary history (if any) may be more complicated than previously thought.

To facilitate discussion, and comparability with previous research, we use the word 'whistle' for toothed whales tonal sounds, however, we do not imply that whistles are necessarily non-homologous to baleen whale tonal sounds – their homology requires further study. We use whistles as a category for some of our analyses, mainly to test the dolphin hypothesis as it was proposed. It is not very informative, however, to simply map the distribution of 'whistles' on a phylogeny (Fig. 1, [see Additional file 1]). Authors differ in their interpretation on the presence or absence of whistles across species, e.g. some define them in the context of a behavior that may have much more limited distribution than the sounds themselves. Furthermore, even within dolphins 'whistles' can be highly variable. We thus highlight the need to focus on the various acoustic parameters (such as frequency variables, modulation, etc.) that may vary independently and have non-identical phylogenetic distributions [see Additional file 1 for rationale]. Hence, our major focus is on such analyses which may reveal which, if any, of the characteristics of 'whistles', or tonal sounds in general, seem associated with sociality.

Our understanding of tonal sound acoustic structure, diversity, and use, is growing, but the evolution of tonal sounds and their association with sociality remains highly speculative. We therefore believe we here improve upon previous studies by providing a more detailed analysis, and using novel and more detailed phylogenies than any study hitherto. We also test these hypotheses across a range of alternative phylogenies.

In sum, we here review current knowledge of both tonal sound production and social structure in Cetacea, and explore the evolution of tonal sounds and the association of individual tonal sound components with sociality (overall social structure and social components). Taking advantage of a new species-level cetacean phylogeny [58,59] we provide the first phylogenetic test of the hypotheses of Herman and Tavolga (1980) [45] and Podos *et al. *(2002) [35]. This study identifies large gaps in knowledge on both traits, and points to key species where future studies would be particularly valuable for enhancing our understanding of the interplay of tonal sounds and sociality.

## Results

### Testing the Dolphin Hypothesis

The following is presented merely to test the dolphin hypothesis as stated (see Introduction, Methods, and [see Additional file 1] for problems with this coarse approach). Under the definition of 'whistle' we use here, the optimization of whistles on the phylogeny is ambiguous (Fig. 1b). However, all of the equally most parsimonious reconstructions reject the dolphin hypothesis. The phylogeny implies that whistles either evolved independently twice, once in *Berardius *and once in the node leading to Delphinida *sensu *Muizon (1988) [60], delphinoids plus river dolphins + *Platanista *(a clade we here refer to as Pandelphinida), with secondary losses in Phocoenidae and within Delphinidae (*Cephalorhynchus *spp. and *Lissodelphis *spp.). Alternatively whistles evolved once in the common ancestor of ziphiids plus pandelphinids and then were subsequently lost thrice in *Hyperoodon*, phocenids and within delphinids (the optimization of whistles is equally ambiguous on previously published phylogenies, [see Additional files 2, 3, 4], while dual origin of whistles is better supported when optimized across the entire set of filtered post-burnin trees, see Methods). Likewise, there are two possible optimizations of sociality under a broad concept approach. One is that sociality evolved in the common ancestor of Odontoceti and was then lost secondarily twice in the riverine species (Fig. 1b). Alternatively sociality may have evolved independently four times (in *Physeter macrocephalus*, within *Ziphiidae*, *Pontoporia*, and in Delphinoidea). The optimization of sociality is ambiguous on over 99% of the alternative trees examined, however, the multiple loss of sociality within Cetacea seems more likely in general, given that relatives of whales are social. Regardless of choice of optimizations, whistles did not originate in the lineage leading to the social dolphins, contra the dolphin hypothesis.

### Character Optimizations

Results of character optimizations led to the same conclusions across all alternative phylogenies examined (previously published hypotheses, [see additional files 2, 3, 4], and post-burnin trees from our Bayesian analysis of Cytochrome b), unless otherwise noted.

**Figure 2:**
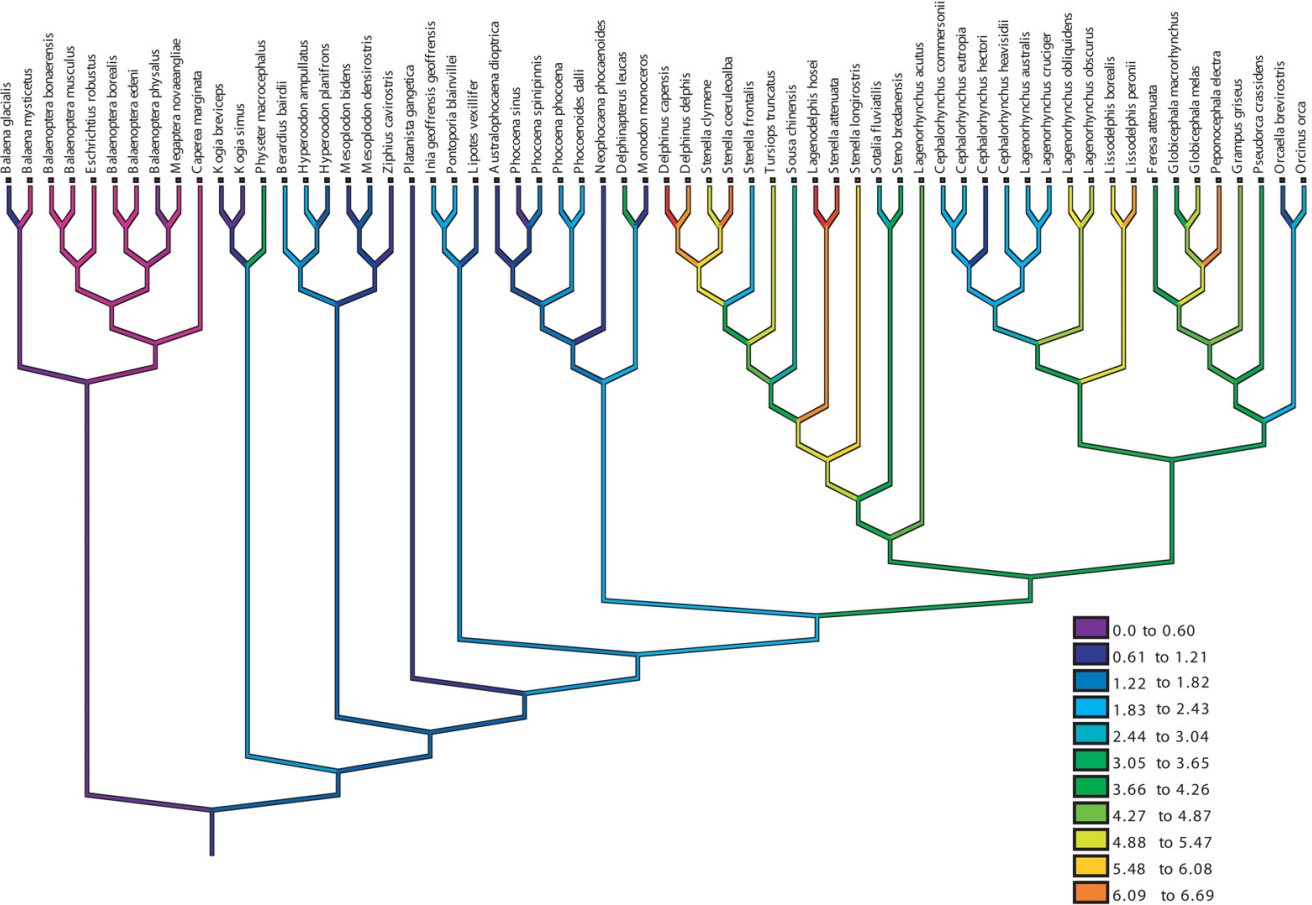
Optimization of group size in Cetacea (using natural log). Dark purple and blue colored branches indicate small groups and demark most of the 'basal' whales. More brightly colored (green, yellow and red) indicate larger groups. The phylogeny suggests gradual increase in group size in the lineage leading to Delphinidae, with independent evolution of huge groups (red) in several lineages and some reversals to smaller groups (e.g. *Cephalorynchus hectori*).

Group sizes in Cetacea [see Additional file 5] appear to have been ancestrally small, but to have gradually increased in the lineage leading to the dolphins, with a number of independent derivations of societies with hundreds of individuals and some secondary reductions in group size (e.g., *Cephalorhynchus *spp, *Orcaella *and *Orcinus *Fig. 2).

Here we present some alternative optimizations of sociality under both a 'broad two and four state concept' framework simply to test the dolphin hypothesis and under a multiple component framework. We note, however, that our study offers limited insights into the evolution of sociality in cetaceans. Future studies will require examining a greater number of component characters of sociality as such data becomes available, and it will require the inclusion of comparative social data also from the outgroups.

**Figure 3:**
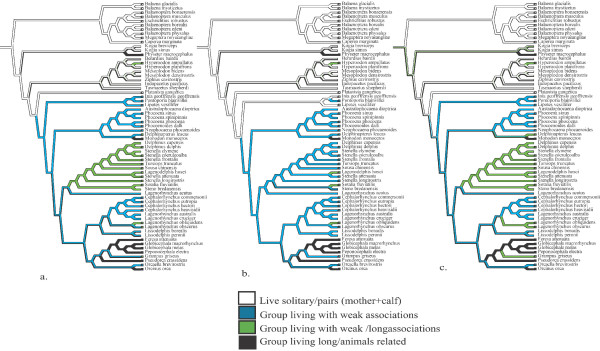
Optimizations of social structure as a four state character (a) leaving polymorphic species as such, (b) lowest social state, (c) highest social state. All analyses were done using the highest social state optimizations (see Methods).

We compare three optimizations of sociality represented as a four-state character (social structure) (see Table 1 and [see Additional file 5]). First, we keep polymorphic species (species reported to show more than one type of social organizations) as such and then compare results when the 'lowest' and 'highest' social state is chosen for each polymorphic species (Fig. 3). All three optimizations have some ambiguity, but optimizations across all trees suggest that family based groups evolved independently at least three times (*Physeter*, *Monodon*, and Globicephalinae Fig. 3). The optimization of social components (including polymorphism) is shown in additional material [see Additional file 6]. Group composition appears to have ancestrally been simple groups consisting only of mother and calf. Segregated (by sex and/or age) and mixed groups may have evolved independently at least four times [see Additional file 6b]. Finally, member associations appear to have evolved from simple mother and calf interactions to complex family based associations [see Additional file 6c].

**Table 1:** Definitions of sociality and tonal sound characters and respective states

SOCIALITY-BROAD CONCEPT APPROACH				
**CHARACTER/STATES**	**0**	**1**	**2**	**3**

SOCIALITY	Species do not live in groups. Mainly found singly or in pairs. Pairs are primarily mother with their calf. Sometimes groups may form but these are temporal (e.g., breeding, feeding, or migration) and do not show any social structure apart from that of mother and calf	Group living species. In addition to mother and calf associations animals are continuously associating with other conspecific. These associations may be short or long-term. Animals within a group may or not be related. Living singly is extremely rare within this species and it is probably limited to old or outcast animals.		
SOCIAL STRUCTURE	Solitary species with strong social bonds limited to the time the calf is dependent of the mother. Animals may aggregate for breeding, feeding, or migration but associations are limited to the duration of these periods. Groups are not socially structured	Group living species where all group members show weak or fluid associations. Both sexes disperse from natal group.	Group living species. Group members show fluid associations but may have long-term associations with specific group members that are not close relatives e.g, male alliances and coalitions. Both sexes disperse from natal group.	Group living species. Group members are close relatives. Natal philopatry is sex dependent but in some species there is no dispersion. Long-term associations.

SOCIALITY-MULTI COMPONENT APPROACH				

**Group Type**	Species described as largely solitary, but that are often found in pairs (mother-calf)	Group living species that are generally found in small groups	Group living species that are generally found in medium to large size schools	
**Group Stability/Associations**	Short when found in non-socially structured groups. Limited to the time the calf is dependent of the mother.	Species where group stability is short. Animals join and leave the group through the day. Described in literature as fluid societies.	Species with fluid societies but were some conspecific group show relatively long lasting associations e.g., male alliances, female nurseries	Species that live in their natal group for life. Animals are related to group members and dispersal is limited showing long-lasting associations
**Group Composition**	Mother and calf	Segregated by age and sex	Mixed (contain both sexes and several ages)	Both segregated and mixed (state only used for the test of association not for optimizations)

TONAL SOUND COMPLEXITY DISCRETE APPROACH				

**Tonal Sound Complexity (2-state)**	Mean inflection point is less or equal to 1	Mean inflection point is more than 1		
**Tonal Sound Complexity (2-state)**	Mean inflection point is between 0–1	Mean inflection point is between 1.1–2	Mean inflection point is between 2.1–3	Mean inflection point is more than 3.1

**Figure 4:**
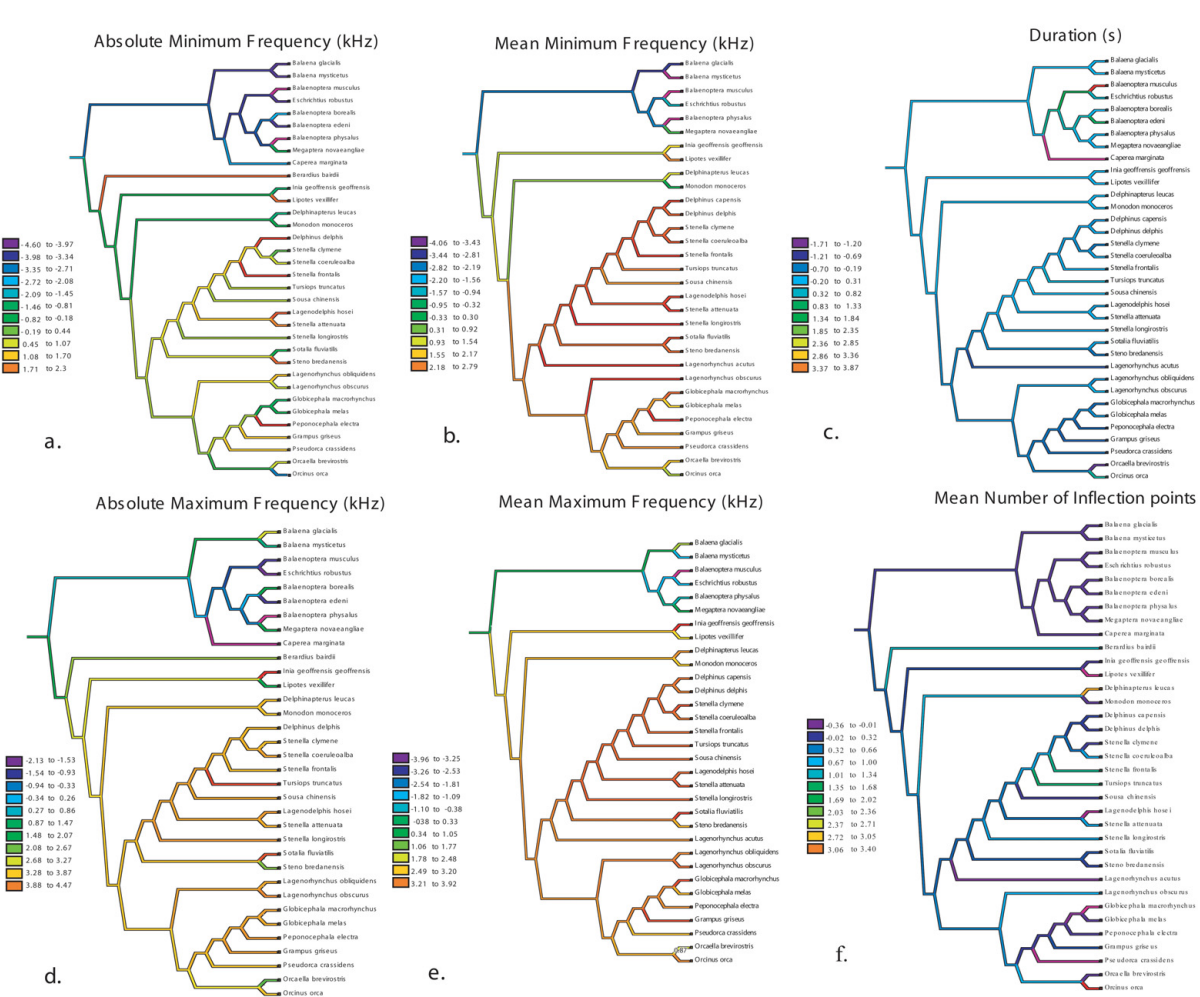
Optimization of Cetacean tonal sound standard acoustic parameters (using natural log). Dark colors (purple and blue) indicate low values, while brighter colors (green, yellow, red) indicate higher values.

Figure 4 shows the optimization of each acoustic character (all transformed using the natural log). Relatively high maximum and minimum frequencies (both absolute and mean) appear derived in toothed whales (Fig. 4a–b, d–e). Particularly high mean maximum and minimum frequencies have evolved within delphinids (note that some of the variation within delphinids and other groups is visually masked by the way Mesquite groups continuous variables in color ranges; [see Additional file 7 for greater detail].

There appears to be a similar trend in the number of tonal sound inflection points (an indicator of tonal sound complexity) going from few ancestrally and increasing in the lineage leading to the dolphins (Fig. 4f). There is an inverse trend in tonal sound duration, where particularly short tonal sounds appear to be derived within the delphinids (Fig. 4c).

### Character regressions and correlations

**Figure 5:**
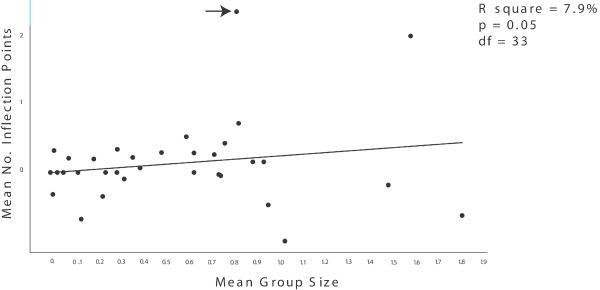
Regression analysis between independent contrasts of mean group size and mean number of inflection points. One conspicuous outlier (arrow) represents a contrast including the killer whale (*Orcinus orca*) which forms relatively small social groups but produces highly modulated whistles. It has been proposed that the killer whale uses whistles in a manner different from any other delphinid to indicate motivational state. That multiple factors are at work shaping tonal sounds in cetaceans may obscure and make difficult to discover true co-evolutinary histories of characters. Accordingly when *O. orca *is removed from the analysis the regression between the two characters becomes stronger.

Under the independent contrast method the regression between group size and the mean number of inflection points was marginally significant: species with larger groups tend to produce tonal sounds with greater mean number of inflection points. Group size explained approximately 7.9% of the variation in inflection points across cetaceans (p = 0.05, df = 33 see Fig. 5 (this and some of the following results are dependent on the choice of phylogeny, see section *Phylogenetic uncertainty*). Group size also significantly explained variation in the mean minimum tonal sound frequency within toothed whales (R^2 ^= 12.4%, df = 23, p-_1tailed _= 0.04). We justify using a one-tailed test based on the expectancy that low frequency sounds travel longer distances so that a priori one might expect that low frequency tended to be associated with solitary species, while species that live their entire lives in large groups need only communicate over short distances. However, given that the two tail test is non-significant we consider this hypothesis only weakly supported. Regressions between group size and other acoustic parameters were not significant.

In addition, there is a significant negative relationship between tonal sound duration and absolute and mean minimum frequency both for all cetaceans (Abs-MinF, R-square = 17%, p = 0.02, df = 31, Mean-MinF, 17.5%, p = 0.02, df = 29) and for toothed whales (Asb-MinF, R-square = 38%, p < 0.001, df = 22, Mean-MinF, R-square = 24%, p = 0.01, df = 23). There was a significant positive relationship between tonal sound duration and complexity for all cetaceans (R-square = 12%, p = 0.04, df = 32) and for toothed whales (R-square = 45%, p < 0.001, df = 23).

Changes in tonal sound complexity were significantly concentrated within social lineages in four of the five most parsimonious reconstructions when both traits were treated as two state characters [see Additional file 8f] .

Tests of character state associations (SIMMAP) show that complex whistles (state 1 = more than one inflection points) were positively associated with group living species (Dij = 0.13, p > 0.999) and negatively with less social species (Dij = -0.024, p < 0.001) treating social complexity as a two state character. In general there was an association between tonal sound complexity and social structure (D_statistic _= 0.376, p < 0.001, Table 2). However, the associations between individual states vary depending on how finely tonal sound and social characters are divided (Table 2). For instance, when treating social complexity as a four state character but tonal complexity as a two state character we find a significant positive association between highly social species (states 2 and 3) and complex tonal sounds and a negative association between complex tonal sounds and 'solitary' (state 0) species (Table 2). When both are treated as four state characters only negative associations are significant (but in the same directions as before, see Table 2).

**Table 2:** Probabilities of association between sociality (selecting the highest social state for polymorphic species) and tonal sound complexity. Significant positive associations at p-values > 0.972 and 0.973** for two and four state complexity characters, respectively and significant negative associations at p-values < 0.028 and 0.027* for two and four state complexity characters, respectively

TONAL SOUND COMPLEXITY [TWO & FOUR STATE CHARACTER]	SOCIAL STRUCTURE [FOUR STATE CHARACTER]			
	
	0	1	2	3
0 (≤ 1 mean inflection point) *D-statistic*	0.0821	0.0536	-0.0424	-0.0047
*p-value*	0.798	0.728	p < 0.0001*	0.003*
1 (≤ 1 mean inflection point) *D-statistic*	-0.0440	0.00045	0.113	0.0360
*p-value*	p < 0.0001*	0.90	0.99**	0.99**
0 (0–1) *D-statistic*	0.084	-0.00029	-0.0338	0.009
*p-value*	0.93	0.055	0.002*	0.88
1 (1.1–2) *D-statistic*	-0.038	0.027	0.0781	0.022
*p-value*	0.002*	0.91	0.92	0.92
2 (2.1–3) *D-statistic*	-0.003	0.0121	0.0198	-0.0033
*p-value*	0.018*	0.89	0.91	0.014*
3 (>3.1) *D-statistic*	-0.0046	0.0151	0.0065	0.0023
*p-value*	0.012*	0.90	0.86	0.84

*Significant negative associations **Significant positive associations

D = 0.362 p < 0.0001, n_p-value _= 1465, n_D _= 2000 Social Structure and Tonal Sound Complexity (4-state)

D = 0.376 p < 0.0001 n_p-value _= 343, n_D _= 2000 Social Structure and Tonal Sound Complexity (2-state)

When three component characters of sociality were analyzed we found similar significant character associations with inflection points (Group size D_statistic _= 0.394, p < 0.001; Group Composition D_statistic _= 0.306, p < 0.001; Stability/Associations D_statistic _= 0.364, p < 0.001, [see Additional file 9 and legend for detail], all indicating association between complex whistles and high levels of sociality.

### Phylogenetic uncertainty

In general, most of our findings are not strongly dependent on the phylogeny of choice, as long as all the species are included. In other words, results in most cases are similar whether the data are analyzed across the trees favored by our own analyses (all post burnin trees and post burnin trees filtered using agreement among multiple studies), or restricted to trees filtered to be congruent with the alternative hypotheses of Messenger and McGuire (1998) [61], Nikaido *et al*. (2001) [62] or Arnason *et al*. (2004) [63], respectively (see Methods for detail). On the all-species phylogenies results significant in the main analyses were also significant across all sets of trees for all SIMMAP analyses. The only difference between analyses was that social and whistle character states were more strongly associated on the trees constrained by the Messenger and McGuire hypothesis than in the remainder [see Additional file 10]. Similarly, the PDAP analyses results agree irrespective of phylogeny choice [see Additional files 11 and 12], except the following. Group size and number of inflexion points correlate significantly except on trees constrained by the hypotheses of Arnason *et al*. (2004) [63] or Nikaido *et al*. (2001) [62], and group size and mean minimum frequency correlate except on trees constrained by the Messenger and McGuire (1998) [61] hypothesis. For ancestral character reconstruction under parsimony, the optimizations of the continuous characters such as group size, tonal sound frequencies, duration, and inflexion points are nearly identical across the trees considered. The optimization of whistles as a presence/absence character was ambiguous on our, and previous, phylogenetic hypotheses. However, on 70% of the filtered post-burnin trees dual origin of whistles was preferred (see above). The optimization of sociality (as a two state character) was ambiguous (single origin followed by multiple losses, or two origins followed by fewer losses), except on the Nikaido *et al*. (2001) [62] hypothesis which favors two origins of sociality. Similarly optimizations of whistles and sociality as multistate characters varied little across trees with no impact on conclusions.

When we used the phylogenies resulting from reanalyzes of the data of Messenger and McGuire (1998) [61], however, significance was lost in a higher number (although not the majority) of the hypotheses tests [see Additional files 10, 11, 12] and some character optimizations changed. Although this can in theory imply sensitivity to phylogenetic pattern, a simpler explanation for this finding seems to be that much of the power of the comparative tests is lost as Messenger and McGuire's data [61] includes only a portion of the species of our main dataset. Hence we do not see a reason to discuss these 'disagreements' further.

## Discussion and conclusion

Our results show that the interplay of tonal sounds and sociality is complicated and that studying the relationship between conglomerate characters such as 'whistles' and 'sociality' largely conceals these intricacies. Under the very simple 'concept approach' the cladistic test [see [64]] rejects the dolphin hypothesis stating that 'whistles' evolved as an adaptation for social communication in dolphins. Whistles, as here defined, appear to be a synapomorphy of pandelphinids, or even a more inclusive group including ziphiids (Fig. 1b). Therefore, the current evidence implies that whistles arose earlier in the evolutionary history of whales than presumed by Podos *et al. *(2002) [35], and whistles are furthermore present in some non-social species, and have been lost more than once within social clades. Apparently then, whistles are not necessary for functional cetacean societies and social communication, and they can play some role in communication in solitary species.

Our findings highlight some of the problems with evolutionary analyses of imprecise, broad concepts. Even though 'whistles' do not correlate with any measure of sociality we find evidence that the evolutionary histories of sociality and tonal sounds are intertwined in the direction suggested by many authors, including Podos *et al. *(2002) [35]. This is evidenced mainly by two findings. (1) The significant association between group size and tonal sound inflection points (complexity) whether tested using independent contrasts, concentrated changes, or character association tests; and (2) the association between group size and minimum tonal sound frequency (and the association of the latter with duration). Simple tonal sounds are mostly confined to species with simple societies (mostly solitary) such as river dolphins and rorquals while tonal sound and social complexity increase in the lineage leading to Delphinoidea (Tables 2). Within that lineage reversal to simpler societies has occurred twice and each time tonal sounds have been secondarily lost (Figs 1a–b, 3), although whistle loss may represent a response to predatory pressure rather than change in social structure (see below).

In addition, especially in toothed whales, species emitting longer tonal sounds tend to show a greater number of inflection points. These observations and tests are congruent with hypotheses stating that complex tonal sounds function as social signals for group cohesion (e.g., most delphinids) during social, traveling, and feeding activities [e.g., [42,65]] or individual recognition (e.g., bottlenose dolphins, Atlantic spotted dolphins) [e.g., [3,37,41,66,67]].

But functionality in a social context can only explain a portion of the variation in tonal sound production and complexity. The secondary loss of tonal sounds in porpoises and the dolphin clade containing *Lagenorhynchus australis*, *L. cruciger *and *Cephalorhynchus spp*, for example, suggests these signals may sometimes be costly, for example in terms of energy production or predation risk. These odontocetes live in very fluid societies where acoustic communication is accomplished by means of rapid pulsed sounds [47,68]. One potential costs of tonal sounds is that these signals may be intercepted (eavesdrop) by an unintended receiver [69,70]. Delphinid tonal sounds are within a frequency range that is readily detected by predators like killer whales which are known to predate on many marine mammal species including these non-whistling species. Furthermore, porpoises and *Cephalorhynchus *seem to have converged upon similar morphology and biosonar systems [71,72], both have ears tuned for high frequency sounds and produce narrowband clicks [73] that are used for echolocation purposes and communication [74,75]. As emphasized by Morisaka and Connor (2007) [76] if killer whales poorly detect these signals, then it may be beneficial for these species to use high frequency signals for social communication [73,74] instead of tonal sounds.

In stable societies like those of *Physeter macrocephalus *and *Orcinus orca*, animals tend to produce group-specific sounds (termed codas and calls respectively) whereas in fission-fusion societieslike those of *Tursiops truncatus *and *Stenella frontalis*, animals produce individual-specific whistles, so called "signature whistles" [see [3,15,41,38]]. Signature whistles are sounds (single-loop and multiple-loop) [see [75]] that to date have only been found in species with fluid societies where mother and calf use them as contact calls and some animals (particularly males) form coalitions (individual recognition may be important when forming these alliances) [e.g., [15,37,38,44,66,67,73,77-82]].

We found evidence for association between group size and the mean minimum frequency, as well as between mean minimum frequency and duration. Given that the former was only marginally significant, we will not place much emphasis on this finding. However, if this finding will be better supported with the addition of further data it may suggest that low minimum frequency (and long duration) is selected for in mostly solitary species which must communicate with other individuals over relatively greater distances than do species that live in permanent societies. It should be noted that May-Collado *et al*. (2007) [59] found a correlation between minimum frequency and body size across whales. This may explain a part of the observed pattern here, as social species are often small, but it remains to be explored if sociality and body size are correlated.

Despite the possible differences in the context in which tonal sounds are produced by riverine dolphins and other delphinoids, there is no *a priori *reason to assume that whistles produced by these toothed whales are not homologous (contra Podos *et al. *2002) [35], and phylogenetically their homology is supported (Fig. 1). It has been proposed that marked deviations of *Inia *from delphinids in scaling relationship in body size and frequency [e.g., [21,83]] is evidence that their sounds are produced by mechanisms different from those used by delphinoids. This is primarily based on the assumption that vertebrate scaling of vocal frequency occurs through size-dependent effects on a common vocal apparatus [e.g. [80]], thus deviations from scaling relationships might indicate an independent proximate mechanism [35]. However, these scaling patterns, for maximum frequency disappear once phylogenetic relationships are taken into account [59].

While some cetacean societies have been studied for a long time, detailed observations are lacking for many species and it is difficult to define and compare levels of sociality across cetacean species. Likewise there are many gaps in our knowledge of tonal sound production [see Additional files 5 and 7]. Our study highlights critical gaps in knowledge, and pinpoints key taxa whose future study could quickly enhance our understanding of the evolution of tonal sounds. As can be seen in Figure 1, tonal sound data would be especially valuable from *Kogia*, ziphiids other than *Berardius*, and from *Platanista *and *Pontoporia*. In a similar manner information on social structure of *Kogia*, *Mesoplodon*, and *Ziphius *would help resolve the optimization of sociality.

Many factors in addition to sociality have been proposed to have influenced the evolution of tonal sounds, including body size and maximum frequency scaling [21,35,59,83,84], habitat [21], predation [76], and zoogeographical [20] and phylogenetic relationships [20,21]. Given that multiple factors are at work true co-evolutionary histories of any given characters could easily be masked. Hence, finding significant correlations between tonal sounds and social structure is particularly interesting. For example, we find a significant, but rather weak, correlation between group size and inflexion points using the independent contrast method. One of the conspicuous outliers in this analysis is *Orcinus orca*, a social delphinid living in relatively small groups that nevertheless produces extremely modulated whistles. Thomsen *et al*. (2001) [85] discuss these extreme modulations and suggest that whistles in killer whales serve a different function than in related dolphins. Removing *O. orca *from the analyses increases the strength of the correlation between whistle complexity and group size (R-square = 9.7%, p-value = 0.03). It should furthermore be noted that comparative biology is fraught with difficulty, getting enough data together for a strong hypothesis testing is typically difficult and missing data results in a loss of power. By accounting for uncertainty in phylogenetic relationships we hope to reduce the rate of type I error. Further, accounting for differences in interpreting and scoring whistle and sociality data attempts to reduce type I error. It is quite possible that in an attempt to avoid type I error we are introducing an unacceptable amount of type II errors. In other words, our ability to detect true character correlations in evolutionary history may be compromised. In this study, however, most of the results were not sensitive to choice of phylogeny or alternative scoring scenarios which adds some confidence to our conclusions.

Our findings point to gaps in knowledge of both tonal sounds and social structure that need to be filled to significantly advance our understanding of their putative co-evolutionary histories. Nevertheless, our results allow us to reject the simple hypothesis that 'whistles' evolved for social communication in dolphins. However, group size explains some of the variation in tonal sound frequency and frequency modulation indicating a special role for complex tonal sounds in a (complex) social context and perhaps for low frequency, long-duration sounds in solitary species. May-Collado and Wartzok (2007) [9] suggested that whistles in *Inia geoffrensis *may be use to keep distance between animals rather than to stimulate social interactions. However, this hypothesis needs to be tested. Future studies should focus on particularly poorly known groups of species such as riverine species, ziphiids, and *Kogia *spp.

## Methods

### Definitions

For purposes of this study the association between tonal sounds and sociality will be studied under both a broad concept [tonal sounds and whistles versus sociality, emulating previous studies], and using a 'component' approach whereby tonal sounds and sociality are dissected into (some of) their component characters. For tonal sounds, standard acoustic parameters we use here include absolute and mean minimum and maximum frequencies (kHz), duration (s), and number of inflection points (a measure of whistle modulation, and a proxy for whistle complexity) [see Additional file 7].

Current knowledge on cetacean sociality indicates the existence of a wide range of social structures, ranging from 'solitary' to highly structured group living species [see [86]]. Generally in the study of cetacean sociality, social species are those that show evidence of group living [87] where animals are associated in a nonrandom fashion [88]. Under the broad concept approach, we have classified species into two general social frameworks, one simply organizing species into non-group living species (state 0) and group living species (state 1) and a second one assigning species to four social types (Table 1, [see Additional file 5]. Under the component approach, we also examine some component characters of sociality for which there is sufficient data available (group size, composition, and stability/associations) either from short and/or long term studies as well as anecdotal observations (Table 1, [see Additional file 5]). Table 1 provides detailed descriptions of these character and their states. It is important to note that for any type of qualitative characterization of sociality, some species may fit into more than one category due to intraspecific variation. For instance, some populations of *Stenella longirostris *have unstable (or 'fluid') groups whose compositions change throughout the day, while populations in the Hawaiian atolls exhibit long-term group fidelity and social stability [89]. These, and other limitations of this study should be kept in mind when interpreting our findings, nevertheless, we believe our approach improves upon previous attempts to detect the associations between sociality and tonal sound production in whales.

### Character Optimizations

Published data on cetacean tonal sound production and sociality were obtained from literature and personal communications [see Additional files 5 and 7]. For tonal sounds we compiled information on the most used acoustic parameters: absolute and mean minimum frequency, absolute and mean maximum frequency, duration, and mean number of inflection points. We only considered studies conducted in the wild or in captivity where, based on the information provided by the authors, it could be assumed species were not recorded in mixed-species groups. We assumed authors were not including harmonics in the acoustic measurements of the tonal sounds emitted by the studied species, unless specified. Information about the social structure of cetaceans was obtained from short to long-term studies, as well as anecdotal information. We searched for information for each of the following social components group size, composition, stability and associations patterns. In addition, information on these social components was used to define four social categories. A minimum of two components was required to place a species within a social category as defined in Table 1. Species for which insufficient components were available were coded as unknown. For species with populations that varied in their social structure or any of the social components ('polymorphic') we selected the highest social state for that particular character. Group size is analyzed as a continuous character using the highest mean group size found in the literature, and also as a discrete character which allows the inclusion of more species [see Additional file 6] since many authors do not provide a mean value but instead offer a description of group sizes.

We relied upon the recent species level phylogenies provide by May-Collado and Agnarsson (2006) [58] and May-Collado *et al*. (2007) [59]. All the main analyses were made using the preferred tree from May-Collado *et al*. (2007) [59] [see Additional file 13]. Because polytomies can compromise character optimization and tests of character correlations, characters were optimized on a fully resolved tree, which is the majority rule tree resulting from a MrBayes analysis (see May-Collado and Agnarsson 2007 for details) [58] without collapsing nodes with less than 50% frequency (using the contype = allcompat option). However analyses were also run on a range of alternative phylogenies (see below) Character optimization was performed with the program Mesquite 1.12 [90], using weighted squared-change parsimony [91].

Acoustic characters were optimized in two data sets (1) with of all cetacean species and (2) pruning species that are known not to emit tonal sounds, species for which acoustic behavior is poorly known, and species that are known to produce tonal sounds but for which detailed information for the character under study was not available. When several values were reported in a species for a particular trait the largest maximum frequency and duration, and the smallest for minimum frequency were used for the analyses [see values in bold in Additional file 7]. Number of inflection points was analyzed both as continuous, reflecting the continuous nature of the data, but also as a two and four state discrete character to facilitate additional analyses that require ordinal data (Table 1, [see Additional files 5 and 9]).

Sociality was optimized as discrete two and four state characters, and using the social components: group size, composition, stability and association patterns (Table 1, [see Additional file 5]). Because several species were polymorphic for one or several characters we optimized species in three ways (1) as polymorphic, (2) emphasizing their 'highest' social level reported, and (3) emphasizing their 'lowest' social level reported. Finally, we analyzed group size as a continuous character.

### Independent Contrasts

Assuming group size as a coarse proxy for social complexity (as defined above by Connor 2000) [87] we regressed it against tonal sound parameters to examine the association of sociality and tonal sound production. Contrasts were calculated using the method of phylogenetically independent contrasts [92]. The method takes into account known dependencies among observations due to phylogenetic relationship of species, and therefore reduces error [93]. Independent contrasts were calculated using the PDAP: PDTREE module [[94], using an unpublished version provided by P. Midford] in Mesquite 1.12 (build h47, 85). To estimate independent contrasts, branch lengths were used as estimated by MrBayes; branch length transformations were necessary for group size (Lack of fit test p < 0.05) and were exponentially transformed. We also tested the relationship between tonal sound frequency and complexity [mean number of inflection points] and tonal sound duration using the independent contrast method.

### Character correlations

We also tested character associations between discrete characters of sociality and tonal sound complexity using two different methods. First we used the software SIMMAP 1.0 [95] which allows for multistate character associations. We did the following tests using all post-burnin trees (n = 2000) from our Bayesian analysis (May-Collado *et al*. 2007) [59] using default settings of the program and employing a rough false discovery rate (FDR) to correct for multiple simultaneous comparisons (critical p values for tests of 8, 12, and 16 comparisons are 0.028 (0.972), 0.027 (0.973), and 0.27 (0.973), respectively). We tested the association of (1) sociality and tonal sound complexity both scored as two state characters, (2) social structure and tonal complexity scored as four state characters, and (3) each of the social components and tonal sound complexity scored as two and four states characters [see Additional file 5]. Second using the concentrated changes test [96] in the software MacClade [97] we tested if changes in tonal sound complexity were concentrated on social branches. For this test we used only two state characters.

It is important to note that testing the role (if any) of sociality in tonal sound evolution is challenging due to the large gaps in our knowledge of cetacean societies, difficulties of objectively defining tonal sound complexity, and levels of sociality, and the limitations of available methods. We note that, as with all of the ordinal data we use here, the divisions between character states are rather arbitrary and open to criticism and alternative coding. Nevertheless we believe that our, be it coarse, phylogenetic approach represents an advance over previous studies that have speculated on social and whistle evolution using less data and lacking a phylogenetic reference. We have tried to test the association of characteristics such as group size and whistle parameters using various different approaches (independent contrast test, concentrated changes test, pairwise comparisons on the phylogeny, and character association test for multistate characters), testing them across various alternative phylogenies, and our results are presented in the form of hypotheses that we hope will subsequently be better tested upon the availability of more data and more sophisticated methods. Also, importantly, our data highlight gaps in knowledge and should guide future studies to where allocating resources might be most beneficial.

### Current Knowledge on Cetacean Sociality and Tonal Sounds

Connor *et al. *1998 [86] and Matthews *et al. *1999 [83] provided brief reviews of the evolution of sociality in toothed whales and tonal sounds in cetaceans, respectively. Connor *et al. *1998 [86] review highlighted the lack of knowledge for most toothed whale species and focused on the social structure of a few species including *Tursiops truncatus*, *Orcinus orca*, *Globicephala spp.*, *Berardius bairdii*, *Physeter macrocephalus*. They compared toothed whale social structure with some terrestrial mammals e.g. elephants and chimpanzees, and found both similarities between the two, but also identified some social elements unique to toothed whales. Matthews *et al. *1999 [83] summarized the frequency and time parameters of 40 cetacean species tonal sounds in relation their body size.

This review summarizes information from 335 sources on sociality and tonal sounds for 64 and 36 Cetacean species, respectively [see Additional files 5 and 7]. The information was gathered from via searches on Web of Science and Google Scholar, and include scientific papers in peer-reviewed journals, conference abstracts, M.Sc. theses, Ph.D. dissertations, technical reports to international organizations, etc.

Although not the main aim of this paper, a few summary statements can be made about current knowledge of sociality and tonal sound production in whales [see Additional files 5 and 7]. Baleen whales have a rather uniform social structure, generally live in simple societies where animals spend considerable time solitary. Weak associations are limited to aggregations form during the breeding and feeding time, and long-term associations appear to be limited to the time mother and calf remained together. In contrast, toothed whale social structure varies enormously, ranging from solitary to species living in huge groups. In groups, group members show an array of association patterns, from weak to stable family associations. For porpoises (Phocoenidae) and several of the freshwater cetacean species (e.g., *Platanista*, *Lipotes*, *Inia*) authors have described group member associations as 'undeveloped', 'weak', or 'fluid'. Such description are difficult to interpret and do not necessarily mean that the authors are suggesting these species live in a fission-fusion society as reviewed in Connor *et al*. 1998 [86] for *Tursiops truncatus*. For most delphinids, association patterns have been described as 'fluid', 'highly fluid fussion-fusion', or 'fluid with short-lasting associations'. In these cases authors appear to imply by 'fluid' that the species do live in fission-fusion societies [as described by [86]]. In these species males tend to form coalitions and alliances to 'capture' and maintain consortship with females. Finally, the most stable social structures have been described in the Sperm whale, (Physeteroidea), most members of the subfamily Globicephalinae, and possibly the Narwhal (Monodontidae). Notably, these species are not all closely related so that "stable" societies have evolved convergently, however, species differ in the degree of dispersal particularly male dispersal from the group.

Our review updates Matthews *et al. *(1999) [79] review on Cetacean tonal sounds. We included recently reported information on species like *Delphinus capensis *and *Sotalia guianensis *[see Additional file 7]. We also updated information on several others like the Narwhal and Beluga (Monodontidae) and the river dolphins *Lipotes *and *Inia *where more data has become available. The previous review [83] included tonal sound information from two beaked whale species (*Mesoplodon densirostris*, *M. carlhubbsi*) that we considered controversial due to the possible pulsative nature of these sounds, thus exclude this information from the table. In addition, *Sousa chinensis *and *Sousa plumbea *were considered here a single species, since no clear evidence yet exists to separate them into two distinct species. Likewise, we consider *Stenella plagiodon *as a synonym of *Stenella frontalis*.

Despite of the increasing knowledge on sociality and tonal sounds the information remains lacking, or scattered, for many species. Here we are highlighting some of these species, particularly key species in the phylogeny that would 'resolve' the ambiguities observed in the evolution of sociality and tonal sounds.

Pygmy and Dwarf sperm whales (*Kogia breviceps *and *K. sima*) [98] are close relatives of the Sperm whale (*Physeter macrocephalus*) a species that shows a matrilineal society and does not produce tonal sounds. There are no indications that these species show a similar society to that of the Sperm whale. In general their social structure and acoustic signals are poorly known [99-104]. Pygmy and Dwarf sperm whales are often seen and strand in small groups that are can be segregated by age and sex or mixed [102], [see Table 1]. The few published accounts on their sounds describe click trains [99,101,103] and cry-like sounds [104] but no tonal sounds.

Beaked Whales (Ziphiidae) are largely unknown. The social structure of the Northern Bottlenose Whale (*Hyperoodon ampullatus*) is the best known of all beaked whales [e.g, [105-109]]. The Baird's Beak Whale (*Berardius bairdii*) is believed to live in stable groups where males may perform parental care [e.g., [86,110,111]]. However, other sources suggest these species live in fission-fusion societies [51]. However both sources report anecdotal evidence and long-term studies are necessary. The social structure of other beaked whales is largely unknown. In terms of tonal sounds, Winn *et al. *(1970) [112] reported whistles in *H. ampullatus*, but it appears to be the general consensus that this species does not produce tonal sounds [e.g. [109], Whitehead pers. comn. 2005]. Tonal sounds have been reported as well in the Cuvier's beaked whale, *Ziphius cavirostris *by Manghi *et al. *(1999) [113] but other acoustic studies only recorded pulsed sounds [e.g., [114,115]]. The only beaked whales for which tonal sounds have been reported are the Baird's Beaked Whale [52] and the Arnoux's Beaked whale (*Berardius arnuxii*) [51]. There is some possibility that the recordings of Dawson *et al. *(1998) [52] were of a sympatric dolphin species (Dawson pers. comm.), however, the recordings of Rogers and Brown (1999) [51] seem conclusive.

*Inia*, *Platanista*, *Lipotes*, *Orcaella*, *Neophocaena *live in freshwater environments. Generally riverine species are considered solitary, however in some areas these species are often seen forming small groups [see Additional file 1 and respective references]. Although, most authors describe group member interactions in riverine species as weak, there is really little knowledge about their societies. In terms of sound production, like the rest of the family (Phocoeenidae) [2], *Neophocaena *does not produce tonal sounds instead the species emits burst pulses under social context [2]. Tonal sounds have been described for two of the subspecies of *Inia geoffrensis*, *Lipotes vexillifer *[see Additional file 7], but not for *Pontoporia *[116]. Mizue *et al. *(1971) [117] reported whistles from *Platanista gangetica*, recorded in captive conditions. However, it is not clear if the animals were acoustically isolated from another riverine dolphin (*I. geoffrensis*), which produces tonal sounds.

The dolphins *Lagenorhynchus cruciger*, *L. australis*, *Lissodelphis *spp, *Steno bredanensis*, *Feresa attenuata*, and *Peponocephala electra *social structure is largely unknown. Most available information comes from stranding and anecdotic information. Although Fish and Turl (1976) [118] documented whistles in *Lissodelphis *spp., recent work did not find whistles (Oswald pers. comn). No published accounts on tonal sounds for *Feresa *and *L. cruciger *were found. May-Collado and Agnarsson (2006) [58] predict that *L. cruciger *may not emit whistles as it nests within a clade of species that do not.

### Phylogenetic uncertainty

Taking phylogenetic relationships among species into account is crucial for hypotheses testing in comparative biology. However, this is no simple procedure – phylogenies themselves are merely hypotheses and for any given comparative study the number of possible alternative phylogenetic arrangements grows exponentially with the number of species being considered. The key question then becomes, how dependent are our conclusions on the choice of phylogeny? Do the results remain mostly unchanged – implying robustness to phylogenetic uncertainty – or do they change when tests are run on alternative "reasonable" phylogenies. Alternative phylogenies can come from several sources, e.g. from previously published independent phylogenetic studies, or from the set of near-optimal trees from a given analysis, e.g. each unique tree from the post burnin set of a Bayesian analysis. If the results of the comparative analyses are different under some of the alternative phylogenies we have not rejected our conclusions but we have been cautioned that the conclusions are dependent on the chosen phylogeny and may be altered as new phylogenetic data become available. If, however, the results are the same across the set of alternative phylogenies then confidence is gained in the conclusions. Here, we attempt to account for phylogenetic uncertainty using various approaches.

The total number of trees in the post-burnin set from the Bayesian analysis is 2000. Instead of basing sensitivity analyses on the 95% credibility set (which includes a number of trees that contradict all recent studies of whale phylogenetics) we use all the post burnin trees filtered based on various constraints reflecting external phylogenetic evidence. This filtering reduces the number of trees facilitating analyses, without much risk of compromising concerns for phylogenetic uncertainty as the constrained clades are, by any standard, uncontroversial. Rather, considering trees that contradict all available phylogenetic evidence would seem more likely to be misleading than useful. Here, we (1) ran analyzes across the post-burnin set of trees from May-Collado *et al*. (2007) [59] filtered by constraining major clades all recent phylogenetic studies of Cetacea agree have supported (see below), and (2) using subsets of the post-burnin trees filtered so as to be congruent with other recently published phylogenetic hypotheses of cetaceans chosen as they are based on various types of data: morphological/palaentological (Geisler 2003) [119], mitogenomic (Arnason *et al*. 2004) [53], a combination of molecular and morphological data (Messenger and McGuire 1998) [61] and SINE's (Nikaido *et al*. 2001) [62]. We chose to use previously published phylogenies as guides to filter trees from the Bayesian post-burnin tree set, rather than to use them directly for analyses (but see below). This is simply because each of these phylogenies contains only a small subset of cetacean species making them poor for the purposes of comparative analyses. Nevertheless, they represent relatively well supported and conflicting hypotheses on the interrelationships of some of the major cetacean clades, whose resolution may impact the findings of our study. Finally, we ran analyses on trees resulting from re-analyses of the Messenger and McGuire dataset, which is the most taxon-rich previously published phylogeny.

We constructed constraint trees in McClade [see Additional file 2] representing each of the previously published phylogeny (see above) and filtered trees from the post-burnin set based on these constraint trees. The constraint trees merely reflect the interrelationships of major clades (families and more inclusive clades, [see Additional file 2]). Species level relationships are not constrained as most of the studies include very few species so that they represent poor tests of lower level phylogenetic structure. Finally, we produced one constraint tree representing only clades that all the previously published studies agree on. This filtering process produced the following datasets: Arnason constraint set (325 trees), Nikaido constraint set (341 trees), Messenger and McGuire constraint set (4 trees), and the all study agreement constraint set (1069 trees). None of the post-burnin trees were congruent with the hypothesis of Geisler (2003) [119]. In fact all other recent molecular, morphological, and combined analyses refute aspects of that hypothesis, in particular the monophyly of all river dolphins (other studies all agree that Platanista is not closely related to the remaining river dolphins), and the monophyly of Physeteroidea (other studies refute the sister relationships of Ziphiidae and Physeteridae). Hence we did not further consider that hypothesis, although it played a role in the construction of the 'all study agreement' subset.

SIMMAP analyses were run across all trees in each subset, while PDAP analyses were conducted on the majority rule tree (using contype = allcompat) of each of the subsets. Furthermore, parsimony ancestral character reconstructions were examined on each of the majority rule trees and across all trees from the all study agreement tree subset.

## Authors' contributions

LJMC, DW, and IA designed the study. LJMC collected the data. IA and LJMC carried out phylogenetic and statistical analyses, and drafted the manuscript. DW assisted with multiple drafts of the manuscript. All authors have read and approved the final version of the manuscript.

## Supplementary Material

Additional file 1 Whistles as a unit for evolutionary analyses. As noted above there are several reasons why using conglomerate concepts like 'whistles' as units of study can hinder progress in the understanding of sound evolution. Apart from being rather arbitrarily defined, and hence differently by different authors, 'whistles' represent a set of characters that may vary independently and may each have different phylogenetic distributions. As a thought experiment let us think of an example where sound production is being compared in two sister lineages. Let us assume that some authors are interested in the evolutionary origin of tonal sounds called 'snorts', and that snorts are defined as narrowband, frequency modulated sounds, with a contour containing at least two inflection points and frequency above 10 kHz. In group A it is noted that sounds are narrowband, frequency modulated, with three inflection points and frequency ranging from 12–15 kHz. In group B sounds are narrowband, frequency modulated, with a contour of two inflection points and frequency ranging from 7–9 kHz. Under a 'broad concept' analysis we would therefore conclude that 'snorts' were present in A, but absent in B, and might conclude that snorts originated in the common ancestor of A (diagram a). However, this belies both the similarities and differences that exist in sound production in the two groups. It denies homology of frequency modulation, contours etc, and even suggests that tonal sounds evolved independently in each group (as 'snorts' are 'different' tonal sounds from non-snorts). Under a 'component' analysis (diagrams b and c), traits like frequency modulation and band width would be scored as identical in the two groups – their similarity would be taken as evidence of common ancestry, i.e. homology. Instead of 'snorts' originating in A, we would more simply explain the differences between the two groups in terms of frequency, and if e.g., the outgroups shared the lower frequency (indicated by white branches) of B we would conclude that a switch to higher frequency (indicated by black branches) occurred in the common ancestor of A (diagram b). In other words, we would learn that the difference between what people call 'snorts' and what they don't call snorts may simply be a matter of sound frequency. In this latter case there is no indication of tonal sound production being non-homologous in A and B, and in fact they share most characteristics of the tonal sounds. Additionally we would learn (diagram c) that inflection points increased from two (white branches) to three (dark branches) in the lineage leading to B (supposing the condition in A was shared with the outgroups). This is information that the concept of 'snorts' obscured. By a component analysis we learn a lot more than by a concept analysis. If we now were interested in the association of sounds and sociality, and group A was social and group B (and outgroups) not, it might be claimed that 'snorts' and 'sociality' are associated and evolved in concert (following diagram a). However, a much more precise and informative conclusion would be that sociality and sound frequency (diagram b) might be related. Hence instead of explaining the social context of 'snorts' we would do well to examine how sound frequency might play an important role in social communication etc. We believe that 'whistles' are no better justified as a unit for evolutionary analysis than 'snorts' in the example above. We do use them in an attempt to test the dolphin hypothesis, but then we opt for a component approach for most of our analyses. Click here for file

Additional file 2 A cetacean phylogeny consistent with Arnason (2004). A majority rule consensus of all post-burnin trees from May-Collado et al. (2007) filtered to be congruent with the mitogenomic phylogeny of Arnason (2004). Numbers on nodes represent posterior probabilities. Click here for file

Additional file 3 A cetacean phylogeny consistent with Messenger and McGuire (1998). A majority rule consensus of all post-burnin trees from May-Collado et al. (2007) filtered to be congruent with the combined morphological and molecular phylogeny of Messenger and McGuire (1998). Numbers on nodes represent posterior probabilities. Click here for file

Additional file 4 A cetacean phylogeny consistent with Nikaido et al. (2001). A majority rule consensus of all post-burnin trees from May-Collado et al. (2007) filtered to be congruent with the SINE phylogeny of Nikaido et al. (2001). Numbers on nodes represent posterior probabilities. Click here for file

Additional file 5 Cetacean social structure and group size. This table reviews published data on cetacean social structure and group size. Numbers in parenthesis correspond to state assigned to each characters as described in Table 1 (bold numbers represent the most common state reported for a particular species). Click here for file

Additional file 6 Optimization of components of sociality. This figure shows social components optimization (a = group size, b = group composition, c = group stability/association patterns) on the preferred phylogeny. Note that this optimization contains polymorphic species and thus family based group like *Physeter *and *Monodon *and species with long-term associations between non-related group members are all optimized using the lowest state of sociality. Click here for file

Additional file 7 Cetacean tonal sound acoustic parameters. This table reviews published data on cetacean tonal sound acoustic parameters. Numbers in bold correspond to the preferred value used in the optimizations (see Methods). Click here for file

Additional file 8 Optimization of tonal sound complexity and the association between sociality and tonal sound complexity. Most parsimonious optimizations of tonal sound complexity (based on mean number of inflection points, MIP) and results from the concentrated changes test for sociality and tonal sound complexity (yellow = state 0, tonal sounds with MIP ≤ 1, blue = state 1, tonal sounds with MIP > 1). Click here for file

Additional file 9 Association between components of sociality and tonal sound complexity. This table summarizes results from SIMMAP analyses of character associations between social components (selecting the highest social state for polymorphic species) and components of tonal sound complexity on the preferred phylogeny. Click here for file

Additional file 10 Association between sociality and tonal sound complexity. This table summarizes results from SIMMAP analyses of character associations between social structure (categorized as 1–4) and tonal sound complexity on the preferred phylogeny across reference phylogenies (see Methods). Click here for file

Additional file 11 Regression between group size and tonal sound characteristics. This table summarizes results from PDAP regression between group size and mean minimum frequency (MMinF) and mean number of inflection points (IP) across reference phylogenies (see Methods). Click here for file

Additional file 12 Regression between duration and other acoustic variables. This table summarizes results from PDAP regression analyses between duration (s) and absolute (AbsMinF) and mean minimum (MMin) frequency and mean number of inflection points (IP) across reference phylogenies (see Methods). Click here for file

Additional file 13 Phylogeny of Cetacea. This figure reproduces the preferred phylogenetic hypothesis of May-Collado et al. (2007), used here for all main analyses. Numbers on nodes represent posterior probabilities. Click here for file

## References

[B1] Au WWL, Au WWL, Popper AN, Fay RR (2000). Hearing in whales and dolphins: An overview. Hearing by Whales and Dolphins.

[B2] Richardson WJ, Green GCJ, Malme CI, Thomsom DH (1995). Marine Mammals and Noise.

[B3] Tyack PL, Mann J, Connor RC, Tyack PL, Whitehead H (2000). Functional aspects of cetacean communication. Cetacean Societies: Field studies of dolphins and whales.

[B4] Barklow W (2004). Amphibious communication with sounds in hippos, *Hippopotamus amphibius*. Animal Behaviour.

[B5] Clark CW, Thomas J, Kastelein RA (1990). Acoustic behavior of mysticete whales. Sensory abilities of cetaceans.

[B6] Oswald JN, Rankin S, Barlow J (2004). The effect of recording and analysis bandwidth on acoustic identification of delphinid species. Journal of the Acoustical Society of America.

[B7] Rasmussen MH, Miller LA (2002). Whistles and clicks from white-beaked dolphins, (*Lagenorhynchus albirostris *Gray 1846) recorded in Faxafloi Bay, Iceland. Aquatic Mammals.

[B8] Boisseau O (2005). Quantifying the acoustic repertoire of a population: the vocalizations of free-ranging bottlenose dolphins in Fiordland, New Zealand. Journal of the Acoustical Society of America.

[B9] May-Collado LJ, Wartzok D (2007). The freshwater dolphin *Inia geoffrensis geoffrensis *produces high frequency whistles. Journal of Acoustic Society of America.

[B10] Azevedo AF, Van Sluys M (2005). Whistles of tucuxi dolphins (*Sotalia fluviatilis*) in Brazil: comparisons among populations. Journal of the Acoustical Society of America.

[B11] Barzúa-Durán MC, Au WWL (2002). Whistles of Hawaiian spinner dolphins. Journal of the Acoustical Society of America.

[B12] Wang X, Wang D, Akamatsu T, Fujita K, Shiraki R (2006). Estimated detection distance of a baiji's (Chinese river dolphin, *Lipotes vexillifer*) whistles using a passive acoustic survey method. Journal of the Acoustical Society of America.

[B13] Lammers MO, Au WWL (2003). Directionality in the whistles of Hawaiian spinner dolphins (*Stenella longirostris*): a signal feature to cue direction of movement?. Marine Mammal Science.

[B14] Rasmussen MH, Lammers M, Beedholm K, Miller LA (2006). Source levels and harmonic content of whistles in white-beaked dolphins (*Lagenorhynchus albirostris*). Journal of the Acoustical Society of America.

[B15] Van Parijs SM, Corkeron PJ (2001). Evidence for signature whistle production by a Pacific humpback dolphin, *Sousa chinensis*. Marine Mammal Science.

[B16] Wang D, Wursig B, Evans WE (1995). Whistles of bottlenose dolphins: comparisons among populations. Aquatic Mammals.

[B17] Barzúa-Durán MC, Au WWL (2004). Geographic variations in the whistles of spinner dolphins (*Stenella longirostris*) of the Main Hawaiian Islands. Journal of the Acoustical Society of America.

[B18] Oswald JN, Barlow J, Norris TF (2003). Acoustic identification of nine delphinids species in the eastern tropical Pacific ocean. Marine Mammal Science.

[B19] Rendell LE, Matthews JN, Gill A, Gordon JCD, MacDonald DW (1999). Quantitative analysis of tonal calls from five odontocete species, examining interspecific and intraspecific variation. Journal of Zoology.

[B20] Steiner WW (1981). Species-specific differences in pure tonal whistle vocalizations of five western North Atlantic dolphin species. Behavioral Ecological and Sociobiology.

[B21] Wang D, Wursig B, Evans WE, Kastelien RA, Thomas JA, Nachtigal PE (1995). Comparisons of whistles among seven odontocete species. Sensory Systems of Aquatic Mammals.

[B22] Wang D, Wursig B, Leatherwwod S (2001). Whistles of boto, *Inia geoffrensis*, and tucuxi, *Sotalia fluviatilis*. Journal of the Acoustical Society of America.

[B23] Parks SE, Tyack PL (2005). Sound production by North Atlantic right whales (*Eubalaena glacialis*) in surface active groups. Journal of the Acoustical Society of America.

[B24] Sirovic A, Hildebrand JA, Wiggins SM, McDonald MA, Moore SE, Thiele D (2004). Seasonality of blue and fin whales calls and the influence of sea ice in the Western Antarctic Peninsula. Deep-Sea Research II.

[B25] Clark CW, Johnson JH (1984). The sounds of the bowhead whale, *Balaena mysticetus*, during the spring migrations of 1979 and 1980. Canadian Journal of Zoology.

[B26] Gedamke J, Costa DP, Dunstan A (2001). Localization and visual verification of a complex minke whale vocalization. Journal of the Acoustical Society of America.

[B27] Stafford KM, Bohnenstiehl DR, Tolstoy M, Chapp E, Mellinger DK, Moore SE (2004). Antarctic-type blue whale calls recorded at low latitudes in the Indian and eastern Pacific Oceans. Deep-Sea Research II.

[B28] Watkins WA, Daher MA, George JE, Rodriguez D (2004). Twelve years of tracking 52-Hz whale calls from a unique source in the North Pacific. Deep-Sea Research I.

[B29] McDonald MA, Hildebrand JA, Wiggins SM, Thiele D, Glasgow D, Moore SE (2005). Sei whale sounds recorded in the Antarctic. Journal of the Acoustical Society of America.

[B30] Frankel AS, Perrin WF, Wursig B, Thewissen JGM (2002). Sound Production. Encyclopedia of Marine Mammals.

[B31] Reidenberg JS, Laitman JT (2007). Discovery of a low frequency sound source in Mysticeti (Baleen Whales): anatomical establishment of a new vocal fold. Anatomical Record.

[B32] Carrington-Stein R (1973). Sound production in Vertebrates: summary and prospectus. American Zoologist.

[B33] Cranford W, Au WWL, Popper AN, Fay RR (2000). In search of impulse sound sources in Odontocetes. Hearing by Whales and Dolphins.

[B34] Cranford W, Amundin M, Norris KS (1999). Functional morphology and homology in the odontocete nasal complex: implications for sound generation. Journal of Morphology.

[B35] Podos J, Da Silva VMF, Rossi-Santos MR (2002). Vocalizations of Amazon river dolphins, *Inia geoffrensis*: insights into the Evolutionary origins of delphinid whistles. Ethology.

[B36] Dreher JJ, Evans WE, Tavolga WN (1964). Cetacean communication. Marine Bioacoustics.

[B37] Caldwell MC, Caldwell DK (1965). Individual whistle contours in bottlenose dolphins (*Tursiops truncatus*). Nature.

[B38] Caldwell MC, Caldwell DK, Miller JF (1973). Statistical evidence for individual signature whistles in the spotted dolphin, *Stenella plagiodon*. Cetology.

[B39] Janik VM, Dehnhardt G, Todt D (1994). Signature whistle variations in bottlenosed dolphin, *Tursiops truncatus*. Behavioral Ecology and Sociobiology.

[B40] Janik VM (2000). Whistle matching in wild bottlenose dolphins (*Tursiops truncatus*). Science.

[B41] Herzing DL, Au WWL, Popper AN, Fay RE (2000). Acoustics and social behavior of wild dolphins: implications for a sound society. Hearing by whales and dolphins.

[B42] Acevedo-Guiterrez A, Stienessen SC (2004). Bottlenose dolphins (*Tursiops truncatus*) increase number of whistles when feeding. Aquatic Mammals.

[B43] Fripp D, Owen C, Quintana-Rizzo E, Shapiro A, Buckstaff K, Jankowski K, Wells R, Tyack P (2005). Bottlenose dolphin (*Tursiops truncatus*) calves appear to model their signature whistles on the signature whistles of community members. Animal Cognition.

[B44] Watwood SL, Tyack PL, Wells RS (2004). Whistle sharing in paired male bottlenose dolphins, *Tursiops truncatus*. Behavioral Ecology and Sociobiology.

[B45] Herman LM, Tavolga WN, Herman LM (1980). The communication systems of cetaceans. Cetacean Behavior: Mechanisms and Functions.

[B46] Watkins WA, Schevill WE, Best PB (1977). Underwater sounds of *Cephalorhynchus heavisidii *(Mammalia:Cetacea). Journal of Mammalogy.

[B47] Watkins WA, Schevill WE (1980). Characteristic features of the underwater sounds of *Cephalorhynchus commersoni*. Journal of Mammalogy.

[B48] Diazgranados MC, Trujillo F (2002). Vocal repertoire of the freshwater dolphins *Inia geoffrensis *and *Sotalia fluviatilis *in Colombia, South America [abstract]. Journal of the Acoustical Society of America.

[B49] Wang D, Wang K, Akamatsu T, Fujita F (1999). Study on whistles of the Chinese River Dolphin or baiji *Lipotes vexillifer*. Oceanologia et Limonologia Sinica.

[B50] Jing X, Youfo X, Rongcai J (1981). Acoustic signals and acoustic behavior of the Chinese River Dolphin (*Lipotes vexillifer*). Scientia Sinica.

[B51] Rogers TL, Brown SM (1999). Acoustic observations of Arnoux's beaked whale (*Berardius arnuxii*) off Kemp Land, Antarctica. Marine Mammal Science.

[B52] Dawson S, Barlow J, Ljungblad D (1998). Sounds recorded from Baird's beaked whale, *Berardius bairdii*. Marine Mammal Science.

[B53] Watkins WA, Schevill WE, Ray C (1970). Underwater sounds of *Monodon *(Narwhal). Journal of the Acoustical Society of America.

[B54] Ford KB, Fisher HD (1978). Underwater acoustic signals of the narwhal (*Monodon monocerus*). Canadian Journal of Zoology.

[B55] Sjare BL, Smith TG (1986). The vocal repertoire of white whales, Delphinapterus leucas, summering in Cunningham Inlet, Northwest Territories. Canadian Journal of Zoology.

[B56] Karlsen JD, Bisther A, Lydersen C, Haug T, Kovacs KM (2002). Summer vocalisations of adult male white whales (*Delphinapterus leucas*) in Svalbard, Norway. Polar Biology.

[B57] Shapiro A (2006). Preliminary evidence for signature vocalizations among free-ranging narwhals (*Monodon monocerus*). Journal of the Acoustical Society of America.

[B58] May-Collado LJ, Agnarsson A (2006). Cytochrome b and Bayesian inference of whale phylogeny. Molecular Phylogenetics and Evolution.

[B59] May-Collado LJ, Agnarsson I, Wartzok D (2007). Reexamining the relationship between body size and tonal signals frequency in whales: a comparative phylogenetic approach. Marine Mammal Science.

[B60] de Muizon C (1988). Les relations phylogénétiques des Delphinida. Annales de Paleontologie.

[B61] Messenger SL, McGuire JA (1998). Morphology, Molecules, and the Phylogenetics of Cetaceans. Systematic Biology.

[B62] Nikaido M, Matsuno F, Hamilton H, Brownell RL, Cao Y, Ding W, Zuoyan Z, Shedlock AM, Fordyce RE, Hasegawa M, Okada N (2001). Retroposon analysis of major cetacean lineages: The monophyly of toothed whales and the paraphyly of river dolphins. PNAS.

[B63] Arnason U, Gullberg A, Janke A (2004). Mitogenomic analyses provide new insights into cetacean origna and evolution. Gene.

[B64] Coddington J (1988). Cladistic tests of adaptational hypotheses. Cladistics.

[B65] Moore SE, Ridgway SH (1995). Whistles produced by common dolphins from Southern California Bight. Aquatic Mammals.

[B66] Janik VM, Slate PJB (1998). Context-specific use suggests that bottlenose dolphin signature whistles are cohesion calls. Animal Behaviour.

[B67] Sayigh LS, Tyack PL, Wells RS, Scott MD (1990). Signature whistles of free-ranging bottlenose dolphins, *Tursiops truncatus*: mother-offspring comparisons. Behavioral Ecology Sociobiology.

[B68] Dawson SM, Thorpe CW (1990). A quantitative analysis of the sounds of Hector's dolphin. Ethology.

[B69] Myrberg AA, Tavolga WN (1981). Sound communication and interception in fishes. Hearing and sound communication in fishes.

[B70] Deecke VB, Ford JKB, Slater PJB (2005). The vocal behaviour of mammal-eating killer whales: communicating with costly calls. Animal Behaviour.

[B71] Watkins WA, Daher MA, Samuels A, Gannon DP (1997). Observations of *Peponocephala electra*, the Melon-headed whale, in the southeastern Caribbean. Caribbean Journal of Science.

[B72] Dudok van Heel WH (1981). Investigations on cetacean sonar. III. A proposal for an ecological classification of cetaceans in relation to sonar. Aquatic Mammals.

[B73] Tyack PL, Owings DH, Beecher MD, Thompson NS (1997). Studying how cetaceans use sound to explore their environment. Perspectives in Ethology.

[B74] Amundin M (1991). Sound production in odontocetes with emphasis on the harbour porpoise, *Phocoena phocoena*.

[B75] Dawson S (1991). Clicks and communication: the behavioral and social contexts of Hector's dolphin vocalizations. Ethology.

[B76] Morisaka T, Connor RC (2007). Predation by killer whales (*Orcinus orca*) and the evolution of whistle loss and narrow-band frequency clicks in odontocetes. Journal of Evolutionary Biology.

[B77] Caldwell MC, Caldwell DK, Tyack PL, Leatherwood SRRR (1990). Review of the signature-whistle hypothesis for the Atlantic bottlenose dolphin. The bottlenose dolphin.

[B78] Caldwell MC, Caldwell DK (1970). Statistical evidence for individual signature whistles in the Pacific whitesided dolphin, *Lagenorhynchus obliquidens*. Cetology.

[B79] Tyack PL (1986). Population biology, social behavior and communication in whales and dolphins. Trends in Ecology and Evolution.

[B80] Tyack PL (1986). Whistle repertoires of two bottlenosed dolphins, *Tursiops truncatus*: mimicry of signature whistles?. Behavioral Ecology and Sociobiology.

[B81] Tyack PL, Reynolds JE, Rommen SA (1999). Communication and cognition. Biology of Marine Mammals.

[B82] Sayigh LS, Tyack PL, Wells RS, Scott MD, Irvine AB (1995). Sex difference in signature whistle production of free-ranging bottlenose dolphins, *Tursiops truncatus*. Behavioral Ecology Sociobiology.

[B83] Matthews JN, Rendell LE, Gordon JCD, MacDonald DW (1999). A review of frequency and time parameters of cetacean tonal calls. Bioacoustics.

[B84] Ryan MJ, Fritzch B, Ryan MJ, Wilczynski W, Walkowiak W, Hetheringon TE (1988). Constraints and patterns in the evolution of anuran acoustic communication. The Evolution of the Amphibian Auditory System.

[B85] Thomsen F, Franck D, Ford JKB (2001). Characteristics of whistles from the acoustic repertoire of resident killer whales (*Orcinus orca*) off Vancouver Island, British Columbia. Journal of the Acoustical Society of America.

[B86] Connor RC, Mann J, Tyack PL, Whitehead H (1998). Social evolution in toothed whales. Trends in Ecology and Evolution.

[B87] Connor RC, Mann J, Connor RC, Tyack PL, Whitehead H (2000). Group living in whales and dolphins. Cetacean Societies: Field studies of dolphins and whales.

[B88] Whitehead H, Bejder L, Ottensmayer A (2005). Testing association patterns: issues arising and extensions. Animal Behaviour.

[B89] Karczmarski L, Würsig B, Gailey G, Larson KW, Vanderlip C (2005). Spinner dolphins in remote Hawaiian atoll: social grouping and population structure. Behavioral Ecology and Sociobiology.

[B90] Maddison WP, Maddison DR (2006). Mesquite: A modular system for evolutionary analysis. Version 1.12. http://mesquiteproject.org.

[B91] Maddison WP (1991). Square-change parsimony reconstructions of ancestral states for continuous-valued characters on a phylogenetic tree. Systematic Zoology.

[B92] Felsenstein J (1985). Phylogenies and the comparative method. American Naturalist.

[B93] Rohlf FJ (2006). A comment on phylogenetic correction. Evolution.

[B94] Midford PE, Garland T, Maddison WP (2005). PDAP Package of Mesquite. http://mesquiteproject.org.

[B95] Bollback JP (2006). SIMMAP: Stochastic character mapping of discrete traits on phylogenies. BMC Bioinformatics.

[B96] Maddison WP (1990). A method for testing the correlated evolution of two binary characters: are gains or losses concentrated on certain branches of a phylogenetic tree?. Evolution.

[B97] Maddison WP, Maddison DR (2003). MacClade: Analysis of Phylogeny and Character Evolution. Version 4.07.

[B98] Chivers SJ, Leduc RG, Robertson KM, Barros NB, Dizon AE (2005). Genetic variation of *Kogia *spp with preliminary evidence for two species of *Kogia sima*. Marine Mammal Science.

[B99] Caldwell DK, Caldwell MC (1971). Sounds produced by two rare cetaceans stranded in Florida. Cetology.

[B100] Caldwell DK, Caldwell MC, Ridgway SH, Harrison Sir H (1989). Pygmy sperm whale *Kogia breviceps *(de Blainville 1838) and dwarf sperm whale *Kogia simus *(Owen 1866). Hanbook of Marine Mammals.

[B101] Caldwell DK, Prescott JH, Caldwell DK (1966). Production of pulsed sounds by the pygmy sperm whale, *Kogia breviceps*. Bulletin Southern California Academy of Science.

[B102] McAlpine DF, Perrin WF, Wursig B, Thewissen JGM (2002). Pygm and Dwarf sperm whales (*Kogia breviceps *and *K. sima*). Encyclopedia of Marine Mammals.

[B103] Marten K (2000). Ultrasonic analysis of pygmy sperm whale (*Kogia breviceps*) and Hubb's beaked whale (*Mesoplodon carlhubbsi*) clicks. Aquatic Mammals.

[B104] Thomas JA, Moore PWB, Nachtigall PE, Gilmartin WG (1990). A new sound from a stranded pygmy sperm whale. Aquatic Mammals.

[B105] Dalebout MK, Hooker SK, Christensen I (2001). Genetic diversity and population structure among northern bottlenose whales, *Hyperoodon ampullatus*, in the western North Atlantic Ocean. Canadian Journal of Zoology.

[B106] Gowans SL, Rendell L (1999). Head-butting in northern bottlenose whales (*Hyperoodon ampullatus*): a possible function for big heads. Marine Mammal Science.

[B107] Gowans S, Whitehead H, Hooker SK (2001). Social organization in northern bottlenose whales, *Hyperoodon ampullatus*: not driven by deep-water foraging?. Animal Behaviour.

[B108] Gowans S, Whitehead H (2001). Photographic identification of northern bottlenose whales (*Hyperoodon ampullatus*): sources of heterogeneity. Marine Mammal Science.

[B109] Hooker SK, Whitehead H (2002). Click characteristics of northern bottlenose whales (*Hyperoodon ampullatus*). Marine Mammal Science.

[B110] Kasuya T (1986). Distribution and behavior of Baird's beake whales off the Pacific coast of Japan. The Scientific Report of Whales Research Institute.

[B111] Kasuya T, Brownell RL, Balcomb KC (1997). Life history of Baird's beaked whales off the Pacific coast of Japan. Reports of the International Whaling Commission.

[B112] Winn HEP, Perkins PJ, Winn L (1970). Sounds and behavior of the northern bottlenose whale [abstract]. Proceedings of the 7th Annual Conference on Biological Sonar and Diving Mammals.

[B113] Manghi M, Montesi G, Fossati C, Pavan G, Priano M, Teloni V (1990). Cuvier's beaked whales in the Ionian Sea: First recordings of their sounds. European Research on Cetaceans.

[B114] Frantzis A, Goold JC, Skarsoulis EK, Taroudakis MI (2002). Clicks from Cuvier's beaked whales (*Ziphius cavirostris*). Journal of the Acoustical Society of America.

[B115] Zimmer WMX, Johnson MP, Madsen PT, Tyack PL (2005). Echolocation clicks of free-ranging Cuvier's beaked whales (*Ziphius cavirostris*). Journal of the Acoustical Society of America.

[B116] Busnell RG, Dziedzic A, Alcuri G (1974). Etudes preliminaries de signoux acoustiques du *Pontoporia blainvillei *Gervais and d'Orbigny 1844 (Cetacea, Platanistidae). Mammalia.

[B117] Mizue K, Nishiwaki M, Takemura A (1971). The underwater sound of ganges river dolphin (*Platanista gangetica*). The Scientific Report of Whales Research Institute.

[B118] Hay KA, Mansfield AW, Ridgway SH, Harrison Sir H (1989). Narwhal, *Monodon monocerus *(Linnaeus 1758). Hanbook of Marine Mammals.

[B119] Fish JF, Turl CW (1976). Acoustic source levels of four species of small whales. Naval Undersea Center Technical Report.

