# Supplementary material for: Phylogenetic review of tonal sound production in whales in relation to sociality
Source: BMC Evol Biol. 2007 Aug 10;7:136. doi: 10.1186/1471-2148-7-136 (PMC2000896; doi:10.1186/1471-2148-7-136)
Supplement: Additional file 1 — Whistles as a unit for evolutionary analyses. As noted above there are several reasons why using conglomerate concepts like 'whistles' as units of study can hinder progress in the understanding of sound evolution. Apart from being rather arbitrarily defined, and hence differently by different authors, 'whistles' represent a set of characters that may vary independently and may each have different phylogenetic distributions. As a thought experiment let us think of an example where sound production is being compared in two sister lineages. Let us assume that some authors are interested in the evolutionary origin of tonal sounds called 'snorts', and that snorts are defined as narrowband, frequency modulated sounds, with a contour containing at least two inflection points and frequency above 10 kHz. In group A it is noted that sounds are narrowband, frequency modulated, with three inflection points and frequency ranging from 12–15 kHz. In group B sounds are narrowband, frequency modulated, with a contour of two inflection points and frequency ranging from 7–9 kHz. Under a 'broad concept' analysis we would therefore conclude that 'snorts' were present in A, but absent in B, and might conclude that snorts originated in the common ancestor of A (diagram a). However, this belies both the similarities and differences that exist in sound production in the two groups. It denies homology of frequency modulation, contours etc, and even suggests that tonal sounds evolved independently in each group (as 'snorts' are 'different' tonal sounds from non-snorts). Under a 'component' analysis (diagrams b and c), traits like frequency modulation and band width would be scored as identical in the two groups – their similarity would be taken as evidence of common ancestry, i.e. homology. Instead of 'snorts' originating in A, we would more simply explain the differences between the two groups in terms of frequency, and if e.g., the outgroups shared the lower frequency (indicated by white br [file 1471-2148-7-136-S1.pdf]

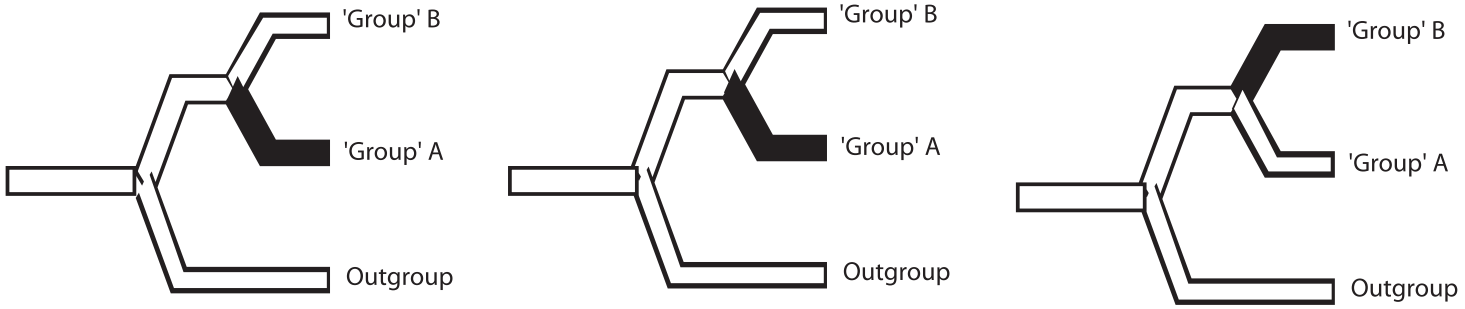

a. Optimization of Snorts
- Present
- Absent

b. Optimization of frequency
- 7-10 kHz
- 12-15 kHz

c. Optimization of inflection points
- 2 inflection points
- 3 inflection points
