# Supplementary material for: Phylogenetic review of tonal sound production in whales in relation to sociality
Source: BMC Evol Biol. 2007 Aug 10;7:136. doi: 10.1186/1471-2148-7-136 (PMC2000896; doi:10.1186/1471-2148-7-136)
Supplement: Additional file 2 — A cetacean phylogeny consistent with Arnason (2004). A majority rule consensus of all post-burnin trees from May-Collado et al. (2007) filtered to be congruent with the mitogenomic phylogeny of Arnason (2004). Numbers on nodes represent posterior probabilities. [file 1471-2148-7-136-S2.pdf]

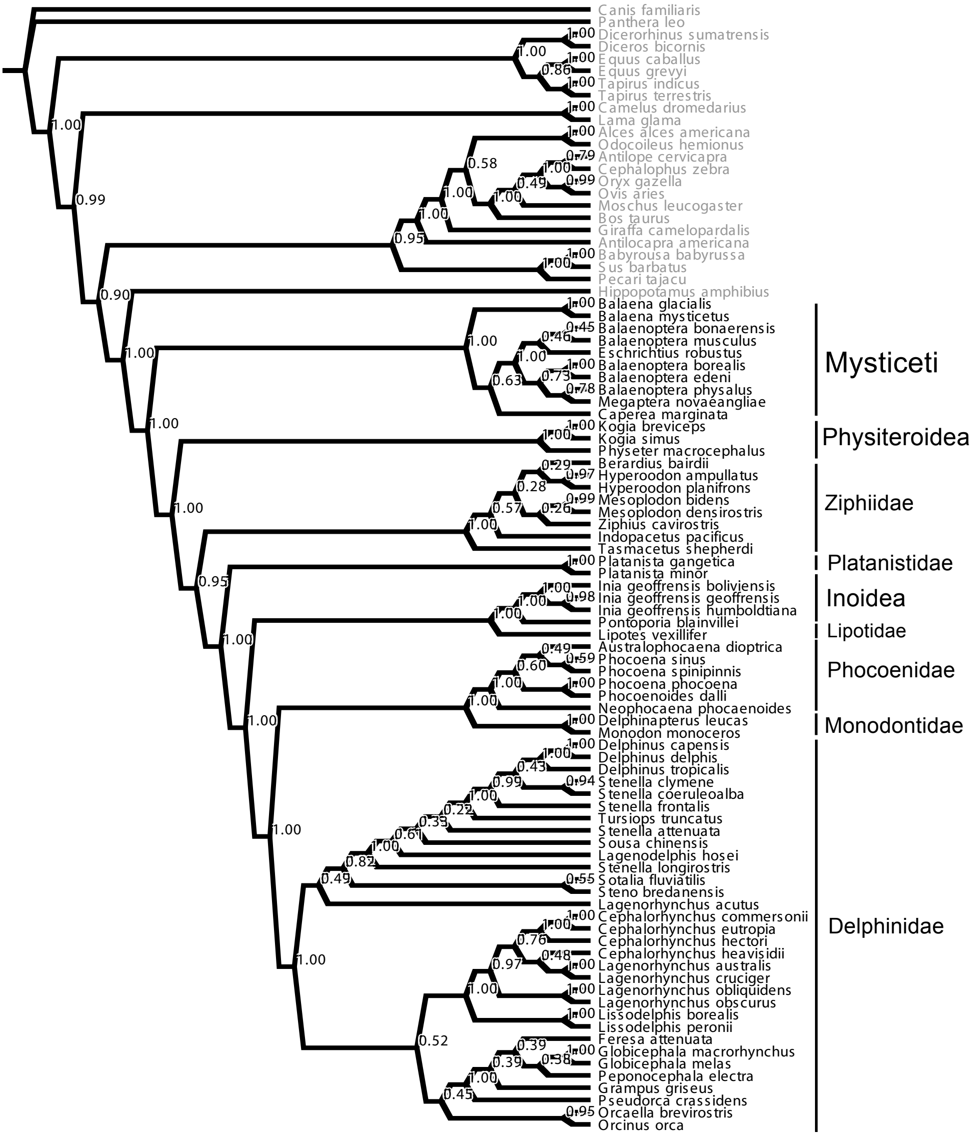

Canis familiaris
Panthera leo
Dicerorhinus sumatrensis
Diceros bicornis
Equus caballus
Equus grevyi
Tapirus indicus
Tapirus terrestris
Camelus dromedarius
Lama glama
Alces alces americana
Odocoileus hemionus
Antilope cervicapra
Cephalophus zebra
Oryx gazella
Ovis aries
Moschus leucogaster
Bos taurus
Giraffa camelopardalis
Antilocapra americana
Babyrousa babyrussa
Sus barbatus
Pecari tajacu
Hippopotamus amphibius
Balaena glacialis
Balaena mysticetus
Balaenoptera bonaerensis
Balaenoptera musculus
Eschrichtius robustus
Balaenoptera borealis
Balaenoptera edeni
Balaenoptera physalus
Megaptera novaeangliae
Caperea marginata
Mysticeti
Kogia breviceps
Kogia simus
Physeter macrocephalus
Physiteroidea
Berardius bairdii
Hyperoodon ampullatus
Hyperoodon planifrons
Mesoplodon bidens
Mesoplodon densirostris
Ziphius cavirostris
Indopacetus pacificus
Tasmacetus shepherdi
Ziphiidae
Platanista gangetica
Platanista minor
Platanistidae
Inia geoffrensis boliviensis
Inia geoffrensis geoffrensis
Inia geoffrensis humboldtiana
Inoidea
Pontoporia blainvillei
Lipotes vexillifer
Lipotidae
Australophocaena dioptrica
Phocoena sinus
Phocoena spinipinnis
Phocoena phocoena
Phocoenoides dalli
Neophocaena phocaenoides
Phocoenidae
Delphinapterus leucas
Monodon monoceros
Monodontidae
Delphinus capensis
Delphinus delphis
Delphinus tropicalis
Stenella clymene
Stenella coeruleoalba
Stenella frontalis
Tursiops truncatus
Stenella attenuata
Sousa chinensis
Lagenodelphis hosei
Stenella longirostris
Sotalia fluviatilis
Steno bredanensis
Lagenorhynchus acutus
Cephalorhynchus commersonii
Cephalorhynchus eutropia
Cephalorhynchus hectori
Cephalorhynchus heavisidii
Lagenorhynchus australis
Lagenorhynchus cruciger
Lagenorhynchus obliquidens
Lagenorhynchus obscurus
Lissodelphis borealis
Lissodelphis peronii
Feresa attenuata
Globicephala macrorhynchus
Globicephala melas
Peponocephala electra
Grampus griseus
Pseudorca crassidens
Orcaella brevirostris
Orcinus orca
Delphinidae
