# Supplementary material for: Phylogenetic review of tonal sound production in whales in relation to sociality
Source: BMC Evol Biol. 2007 Aug 10;7:136. doi: 10.1186/1471-2148-7-136 (PMC2000896; doi:10.1186/1471-2148-7-136)
Supplement: Additional file 3 — A cetacean phylogeny consistent with Messenger and McGuire (1998). A majority rule consensus of all post-burnin trees from May-Collado et al. (2007) filtered to be congruent with the combined morphological and molecular phylogeny of Messenger and McGuire (1998). Numbers on nodes represent posterior probabilities. [file 1471-2148-7-136-S3.pdf]

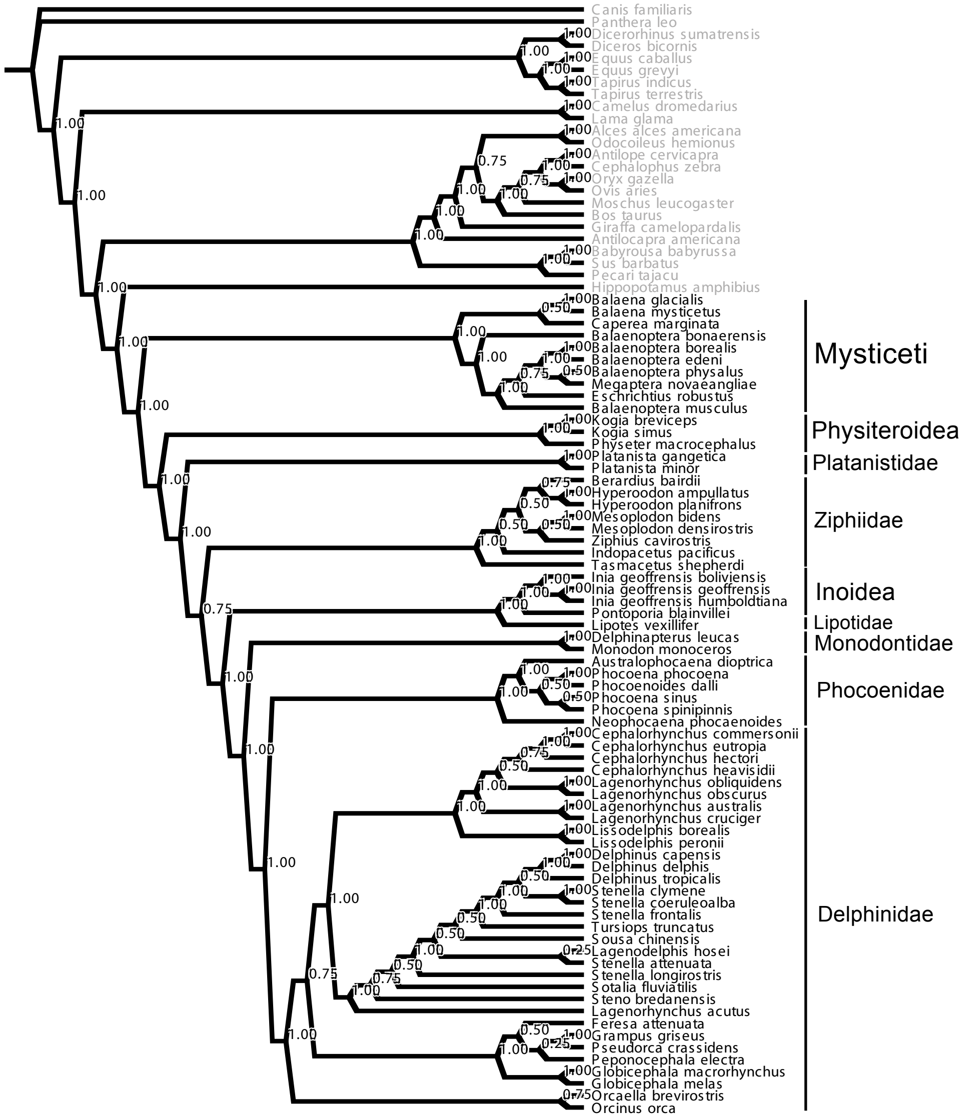

Canis familiaris
Panthera leo
Dicerorhinus sumatrensis
Diceros bicornis
Equus caballus
Equus grevyi
Tapirus indicus
Tapirus terrestris
Camelus dromedarius
Lama glama
Alces alces americana
Odocoileus hemionus
Antilope cervicapra
Cephalophus zebra
Oryx gazella
Ovis aries
Moschus leucogaster
Bos taurus
Giraffa camelopardalis
Antilocapra americana
Babyrousa babyrussa
Sus barbatus
Pecari tajacu
Hippopotamus amphibius
Balaena glacialis
Balaena mysticetus
Caperea marginata
Balaenoptera bonaerensis
Balaenoptera borealis
Balaenoptera edeni
Balaenoptera physalus
Megaptera novaeangliae
Eschrichtius robustus
Balaenoptera musculus
Kogia breviceps
Kogia simus
Physeter macrocephalus
Platanista gangetica
Platanista minor
Berardius bairdii
Hyperoodon ampullatus
Hyperoodon planifrons
Mesoplodon bidens
Mesoplodon densirostris
Ziphius cavirostris
Indopacetus pacificus
Tasmacetus shepherdi
Inia geoffrensis boliviensis
Inia geoffrensis geoffrensis
Inia geoffrensis humboldtiana
Pontoporia blainvillei
Lipotes vexillifer
Delphinapterus leucas
Monodon monoceros
Australophocaena dioptrica
Phocoena phocoena
Phocoenoides dalli
Phocoena sinus
Phocoena spinipinnis
Neophocaena phocaenoides
Cephalorhynchus commersonii
Cephalorhynchus eutropia
Cephalorhynchus hectori
Cephalorhynchus heavisidii
Lagenorhynchus obliquidens
Lagenorhynchus obscurus
Lagenorhynchus australis
Lagenorhynchus cruciger
Lissodelphis borealis
Lissodelphis peronii
Delphinus capensis
Delphinus delphis
Delphinus tropicalis
Stenella clymene
Stenella coeruleoalba
Stenella frontalis
Tursiops truncatus
Sousa chinensis
Lagenodelphis hosei
Stenella attenuata
Stenella longirostris
Sotalia fluviatilis
Steno bredanensis
Lagenorhynchus acutus
Feresa attenuata
Grampus griseus
Pseudorca crassidens
Peponocephala electra
Globicephala macrorhynchus
Globicephala melas
Orcaella brevirostris
Orcinus orca
Mysticeti
Physiteroidea
Platanistidae
Ziphiidae
Inoidea
Lipotidae
Monodontidae
Phocoenidae
Delphinidae
