# Supplementary material for: Phylogenetic review of tonal sound production in whales in relation to sociality
Source: BMC Evol Biol. 2007 Aug 10;7:136. doi: 10.1186/1471-2148-7-136 (PMC2000896; doi:10.1186/1471-2148-7-136)
Supplement: Additional file 5 — Cetacean social structure and group size. This table reviews published data on cetacean social structure and group size. Numbers in parenthesis correspond to state assigned to each characters as described in Table 1 (bold numbers represent the most common state reported for a particular species). [file 1471-2148-7-136-S5.doc]

| Species | Sociality 2-states  Character | Sociality  4-states  Character | Group  Mean Size | Sociality components | | | References |
| --- | --- | --- | --- | --- | --- | --- | --- |
| **Baleen Whales**  **(Mysticeti)**  Balaenidae |  |  |  | Description of Group Size | Stability/  Associations | Composition |  |
| *Eubalaena glacialis*** | 0 | 0 | 2.57 | -Singly, Pairs, **(0)**  -Breeding and feeding grounds aggregations, (3) | -Short (except for mother and calf) **(0)**  **-** Weak associations when found in groups**(1)** | -Pairs Mother+ Calf **(0)**  -Groups segregated by sex and age, Mixed (1,2) | 1, 2,3 |
| *Balaena mysticetus* | 0 | 0 | 1 | -Singly, Pairs, **(0)**  -Breeding and feeding grounds aggregations, (3) | Short (except for mother and calf) **(0)**  **-** Weak associations when found in groups**(1)** | -Pairs Mother+ Calf **(0)**  -Groups segregated by sex and age, Mixed (1,2) | 3, 4, 5 |
| Balaenopteridae |  |  |  |  |  |  |  |
| *Balaenoptera borealis* | 0 | 0 | 1 | -Singly, Pairs, **(0)**  -Breeding and feeding grounds aggregations, (3) | Short (except for mother and calf) **(0)**  **-** Weak associations when found in groups **(1)** | -Pairs Mother+ Calf **(0)**  -Groups segregated by sex and age, Mixed (1,2) | 3,6 |
| *B. bonaerensis* | 0 | 0 | 1 | -Singly, Pairs, **(0)**  - Breeding and feeding grounds aggregations, (3) | Short (except for mother and calf) **(0)**  **-** Weak associations when found in groups **(1)** | -Pairs Mother+ Calf **(0)**  -Groups segregated by sex and age, Mixed (1,2) | 3,7 |
| *B. edeni/ B. brydei* | 0 | 0 | 1 | -Singly, Pairs, **(0)**  -Breeding and feeding grounds aggregations, (3) | Short (except for mother and calf) **(0)**  **-** Weak associations when found in groups **(1)** | -Pairs Mother+ Calf **(0)**  -Groups segregated by sex and age, Mixed (1,2) | 3, 8, 9 |
| *B. musculus* | 0 | 0 | 1 | -Singly, Pairs, **(0)**  -Breeding and feeding grounds aggregations, (3) | Short (except for mother and calf) **(0)**  **-** Weak associations when found in groups **(1)** | -Pairs Mother+ Calf **(0)**  -Groups segregated by sex and age, Mixed (1,2) | 3 |
| *B. physalus* | 0 | 0 | 1.55 | -Singly, Pairs, **(0)**  -Small groups, (1)  -Breeding and feeding grounds aggregations, (3) | Short (except for mother and calf) **(0)**  **-** Weak associations when found in groups **(1)** | -Pairs Mother+ Calf **(0)**  -Groups segregated by sex and age, Mixed (1,2) | 3, 9-13 |
| *Megaptera novaeangliae* | 0 | 0 | 1 | -Singly, Pairs, **(0)**  - Breeding and feeding grounds aggregations, (3) | Short (except for mother and calf) **(0)**  **-** Weak associations when found in groups **(1)** | -Pairs Mother+ Calf **(0)**  -Groups segregated by sex and age, Mixed (1,2) | 3, 9, 14- 22 |
| Eschrichtidae |  |  |  |  |  |  |  |
| *Eschrichtius robustus* | 0 | 0 | 1 | -Singly, Pairs, **(0)**  - Breeding and feeding grounds aggregations, (3) | Short (except for mother and calf) **(0)**  **-** Weak associations when found in groups **(1)** | -Pairs Mother+ Calf **(0)**  -Groups segregated by sex and age, Mixed (1,2) | 3, 23-24 |
| Neobalaenidae |  |  |  |  |  |  |  |
| *Capera marginata* | 0 | 0 | 1 | -Singly, Pairs, **(0)**  - Breeding and feeding grounds aggregations, (3) | Short (except for mother and calf) **(0)**  **-** Weak associations when found in groups **(1)** | -Pairs Mother+ Calf **(0)**  -Groups segregated by sex and age, Mixed (1,2) | 3 |
| **Toothed Whales**  **(Odontoceti)**  Kogiidae |  |  |  |  |  |  |  |
| *Kogia breviceps*** | ? | ? | ~2 | -Singly, pairs (stranded animals) **(0)**  -Small group **(1)** | Unknown (except for the mother and calf) **(?)** | -Segregated by sex and age (possibly) **(1)**  -Mixed (possibly) **(2)** | 25-26 |
| *K. simus* | ? | ? | 1.87 | -Singly (stranded animals) (0)  -Small groups **(1)** | Unknown (except for the mother and calf) **(?)** | -Segregated by sex and age (possibly) **(1)**  -Mixed (possibly) **(2)** | 25-31 |
| Physeteridae |  |  |  |  |  |  |  |
| *Physeter macrocephalus* | 1 | 3 | 22.1 | -Solitary adult males **(0)**  -Small Female + calves (nursery groups) **(1)**  -Immature males groups (1) | -Weak associations in immature male groups **(1)**  -Long associations in Matrilineal groups **(3)** | -Segregated by sex and age **(1)** | 27, 32-40 |
| Ziphiidae |  |  |  |  |  |  |  |
| *Berardius bairdii (B. anurxii)* | 1 | ? | 7.2 | -Small groups (male biased) **(1)**  -Large Aggregations when traveling (3) | Unknown (except for the mother or father and calf in this case) **(?)**  - Males possibly do parental care but it is not clear what kind of associations they have **(?)** | -Mixed **(2)** | 41-48 |
| *Hyperoodon ampullatus* | 1 | 2 | 7 | -Small groups **(1)** | Fluid associations except for long-term associations between males **(2)** | -Segregated by sex and age **(1)** | 49-51 |
| *H. planifrons* | ? | ? | 3.61 | -Small groups **(1)** | Unknown (except for the mother and calf) **(?)** | Unknown **(?)** | 52 |
| *Mesoplodon bidens* | ? | ? | 3 | -Small groups **(1)** | Unknown (except for the mother and calf) **(?)** | Mixed **(2)** | 53-55 |
| *M. densirostris* | ? | ? | 3.7 | -Singly (strandings data), pairs **(0)**  -Small groups **(1)** | Unknown (except for the mother and calf) **(?)** | -Mother+calf **(0)**  -Mixed **(2)** | 54-58 |
| *Ziphius cavirostris* | ? | ? | 2.9 | -Singly (strandings data), pairs **(0)**  -Small groups **(1)** | Unknown (except for the mother and calf) **(?)** | -Mother+calf **(0)**  -Mixed **(2)** | 27, 28, 56, 59-62 |
| Platanistidae |  |  |  |  |  |  |  |
| *Platanista gangetica gangetica*  *Platanista gangetica minor* | 0 | 0 | 2.45 | -Singly, pairs (Mother+calf) (most common) **(0)**  -Aggregations (3) | -Relatively long for mother and calf **(0)**  -**W**eak associations when found in groups **(1)** | Mother+calf **(0)**  Unknown **(?)** | 27, 63-71 |
| Iniidae |  |  |  |  |  |  |  |
| *Inia geoffrensis* | 0 | 0/1 | 6.22 | -Singly (strandings data), pairs **(0)**  -Small groups **(1)**  -Aggregations in breeding and feeding grounds (3) | -Relatively long for mother and calf **(0)**  -**W**eak associations when found in groups **(1)** | -Mother+ Calf **(0)**  -Single sex **(1)**  -Mixed **(2)** | 27, 72*-*78 |
| Pontoporidae |  |  |  |  |  |  |  |
| *Pontoporia blainvillei* | 1 | 1 | 7.1 | -Solitary animals are rare (0)  -Small groups (traveling, feeding, socializing) **(1)** | -Weak associations (described as ‘fluid’) **(1)** | -Mixed **(2)** | 79-82 |
| Lipotidae |  |  |  |  |  |  |  |
| *Lipotes vexillifer* | 0 | 0/1 | 3.4 | -Singly, pairs (Mother+calf) (0)  -Small (most common) **(1)**  -Aggregations (3) | -Relatively long for mother and calf **(0)**  -**W**eak associations when found in groups **(1)** | Mother+calf **(0)**  Unknown **(?)** | 27, 83-86 |
| Phocoenidae |  |  |  |  |  |  |  |
| *Phocoena dioptrica* | 1 | ? | 3 | -Singly (0)  -Small groups **(1)** | -Unknown (except for the mother and calf) **(?)** | Mother+calf (0)  Unknown **(?)** | 87-89 |
| *Phocoena phocoena* | 1 | 1 | 5.7 | -Single **(0)**  -Pairs (most common)  -Small groups (most common) **(1)**  -Aggregations (3) | -Relatively long for mother and calf **(0)**  -**W**eak associations when found in groups **(1) –described as ‘fluid’** | Mother+calf **(0)**  Unknown **(?)** | 27, 90-94 |
| *P. sinus* | 1 | 1 | 2 | -Single  -Pairs (most common) **(0)**  -Small groups (1) | -Relatively long for mother and calf **(0)**  -**W**eak associations when found in groups **(1) –described as ‘fluid’** | Segregated by sex and age (possibly) **(1)** | 27, 95*-*97 |
| *P. spinipinnis* | 1 | ? | 4.5 | -Small (most common) **(1)**  -Aggregations (3) | -Unknown (except for the mother and calf) possibly short **(?)** | Mother+calf (0)  Unknown **(?)** | 27 |
| *Phocoenoides dalli* | 1 | 1 | 7.4 | -Single (sometimes) (0)  -Small groups (most common) **(1)**  -Large feeding aggregations (rare) (3) | -Relatively long for mother and calf **(0)**  -**W**eak associations when found in groups **(1) –described as ‘fluid’** | Segregated by sex and age (possibly) **(1)** | 27, 98-99 |
| *Neophocaena phocaenoides* | 1 | 1 | 3 | -Pairs (most common) **(0)**  -Small groups (most common in Yangtzee) **(1)** | -Relatively long for mother and calf **(0)**  -**W**eak associations when found in groups **(1) –described as ‘undeveloped’** | Mother+calf **(0)**  Unknown **(?)** | 27, 100-104 |
| Monodontidae |  |  |  |  |  |  |  |
| *Monodon monocerus* | 1 | 3 | 3 | -Small groups (most common) **(1)**  -Large schools (2) | -Short and fluid assocaitons (possibly in large groups) **(1)**  -Matrilineal (described as possibly ‘matrifocal’) **(3)** | -Segregated by sex and age (possibly) **(1)**  -Mixed **(2)** | 27, 105-110 |
| *Delphinapterus leucas* | 1 | 1/2 | 32.9 | -Singly (0)  -Small groups (most common in some areas) **(1)**  -Schools (most common in some areas) **(2)**  -Large Aggregations in breeding areas (3) | -Relatively long for mother and calf **(0)**  -**W**eak associations when found in groups **(1) –described as ‘fluid’** | Segregated by age and sex **(1)** | 27, 111-119 |
| Delphinidae |  |  |  |  |  |  |  |
| *Cephalorhynchus commersonii* | 1 | 1 | 6.9 | -Small groups **(1)** | -Weak (described as ‘fission-fusion’) **(1)** | Mother+calf **(0)**  Unknown **(?)** | 27, 120-121 |
| *C. eutropia* | 1 | 1 | 10 | -Small groups **(1)** | -Weak (described as ‘fission-fusion’) **(1)** | Mother+calf **(0)**  Unknown **(?)** | 120,122 |
| *C. hectori* | 1 | 1 | 8 | -Small groups **(1)** | -Weak (described as ‘fission-fusion’) **(1)** | Segregated by age and sex (possibly) **(1)** | 123-127 |
| *C. heavisidii* | 1 | 1 | 3.2 | -Small groups **(1)** | -Weak (described as ‘fission-fusion’) **(1)** | Mother+calf **(0)**  Unknown **(?)** | 128, 120 |
| *Lagenorhynchus australis* | ? | ? | 6.92 | -Small groups **(1)**  -Aggregations (rare) (3) | -Unknown (except for the mother and calf) **(?)**  -Weak (described as ‘fission-fusion’) **(1)** | Mother+calf **(0)**  Unknown **(?)** | 129-131  131b |
| *L.cruciger* | ? | ? | 7 | -Small groups **(1)** | - Unknown (except for the mother and calf) **(?)** | Mother+calf **(0)**  Unknown **(?)** | 132-133 |
| *L. obliquidens* | 1 | 1 | 127.38 | -Medium sized groups **(2)**  -Large schools (small units within) | -Weak associations (possible strong male associations) **(1,2)** | Segregated by age and sex **(1)** | 27, 129*,* 134-136 |
| *L. obscurus* | 1 | 1/2 | 86 | -Small groups (1)  -Medium sized groups (most common) **(2)**  -Large schools | -Weak **(1) -described as ‘fission-fusion’**  -Some long term associations **(2)** | Segregated by age and sex **(1)** | 27, 137-141 |
| *L. acutus* | 1 | 1 | 53.2 | -Small groups (most common in some areas) **(1)**  -Medium (most common in some areas) **(2)**  -Large schools | -Unknown (except for the mother and calf) possibly short **(?)** | Segregated by age and sex (based on strandings) **(1)** | 27, 142-144 |
| *Lissodelphins borealis* | 1 | ? | 110.2 | -Singly (0)  -Large schools (most common) **(2)** | -Unknown (except for the mother and calf) possibly short **(?)** | Mother+calf **(0)**  Unknown **(?)** | 27, 145-147 |
| *L. peronii* | 1 | ? | 210 | -Singly (0)  -Small groups (1)  -Large schools (most common) **(2)** | -Unknown (except for the mother and calf) possibly short **(?)** | Mother+calf **(0)**  Unknown **(?)** | 146, 148-150 |
| *Delphinus delphis* | 1 | 1/2 | 230.38 | -Small subunits (within large and Medium size schools) **(1)**  -Medium sized groups (most common in some areas) **(2)**  -Large schools (most common in some areas)  -Large aggregations (3) | -Strong subunits with possible related animals **(3)**  -Weak associations-**described as ‘highly fluid fission-fusion social system’** **(1)** | Segregated by age and sex (possibly) **(1)** | 28, 136, 151-155 |
| *Delphinus capensis* | 1 | ? | 411.69 | -Large schools **(2)** | -Unknown (except for the mother and calf) possibly short **(?)** | Mother+calf **(0)**  Unknown **(?)** | 136 |
| *Stenella attenuata* | 1 | 1/2 | 360 | -Small groups (1)  -Medium sized groups **(2)**  -Large schools | -Strong associations (possibly within male groups) **(2)**  -Weak associations (described as ‘fluid’) **(1)** | Segregated by age and sex **(1)** | 27-28, 156-162 |
| *S. clymene* | 1 | ? | 97.4 | -Small groups (1)  -Medium sized groups **(2)**  -Large schools | -Unknown (except for the mother and calf) possibly short **(?)** | Segregated by age and sex (based on strandings) **(1)** | 27-28, 136, 163-167 |
| *S. frontalis* | 1 | 1/2 | 10 | -Small groups **(1)** | -Strong associations (within male groups) **(2)**  -Weak associations (described as ‘fluid’) **(1)** | Segregated by age and sex **(1)** | 27, 168-173 |
| *S. coeruleoalba* | 1 | 1/2 | 302 | -Small (most common in some areas) **(1)**  -Medium (most common in some areas) **(2)**  -Large schools (most common in some areas) | -Strong associations (possibly within male groups) **(2)**  -Weak associations (described as ‘fluid’) **(1)** | -Segregated by age and sex **(1)**  -Mixed **(2)** | 27-28, 136, 174-175 |
| *S. longirostris* | 1 | 1/2 | 147.74 | -Large schools **(2)** | -Weak associations (described as ‘fluid’) **(1)**  -Strong (described as ‘strongly bonded’) for other group members not necessarily just males) **(2)** | -Segregation by sex and age **(1)** | 27-28, 136, 177*,* 179*,* 180*,* 178*,* 176, 181*,* |
| *Tursiops truncatus* | 1 | 1/2 | 92-2 | -Small groups (most common in some areas) **(1)**  -Medium sized groups (most common in some areas) **(2)**  -Large schools (most common in offshore ecotype) | -Strong (males coalitions and alliances) **(2)**  -Weak (described as ‘fission-fusion’) **(1)** | -Segregation by sex and age **(1)** | 27-28, 136, 182-*199* |
| *Lagenodelphis hosei* | 1 | 2 | 440.05 | -Large schools **(2)** | -Strong (described as more strong than other social dolphins like *Stenella*) **(2)** | -Mixed (based on strandings) **(2)** | 27, 136, 200*-*204 |
| *Sousa chinensis* | 1 | 1/2 | 14.9 | -Solitary large adults (0)  -Small groups (most common) **(1)**  -Medium sized groups (rare) | -Weak (described as ‘fluid with short-lasting associations’) **(1)**  -Strong mother +calf **(0)**  -Strong (affiliations in stable groups from Mozambique) **(2)** | -Segregation by sex and age **(1)**  -Mixed (all age classes) **(2)** | 27, 205-210 |
| *Sotalia fluviatilis (riverine) Sotalia guianensis (marine)* | 1 | 2 | 13 | -Singly (both species) (0)  -Small (riverine) (most common) **(1)**  -Medium (marine) (2)  -Large feeding aggregations (marine) (3) | -Weak associations **(1)**  -‘Family’ (2adults+calf) described in the marine species **(2)** | -Mixed **(2)** | 211-218 |
| *Steno bredanensis* | 1 | ? | 40 | -Small (most common in some areas) **(1)**  -Medium (most common in some areas) **(2)**  -Large aggregations (3) | Unknown (except for the mother and calf) **(?)** | -Mixed (based on strandings but largely unknown) **(2)** | 27-28, 136, 219-223 |
| *Feresa attenuata* | 1 | ? | 30.12 | -Small (more common in some areas) **(1)**  -Medium sized groups (more common in some areas) **(2)**  -Large schools (rare) | -Strong (possibly similar to other globicephaliinids were individuals are related) **(3)** | -Mixed (based on strandings but largely unknown) **(2)** | 27, 136, 224-227 |
| *Globicephala macrorhynchus* | 1 | 3 | 41.1 | -Small groups **(1)**  -Medium sized groups **(2)** | -Matrilineal (natal philopatry, males live the group) **(3)** | -Mixed **(2)** | 27-28, 45, 136, 228-234 |
| *G. melas* | 1 | 3 | 84.5 | -Medium sized groups **(2)**  -Large schools | -Matrilineal (natal philopatry, males live the group) **(3)**  -Weak (some evidence of short term associations) **(1)** | -Mixed **(2)** | 27, 228*-*229, 231-232, 234*,* 235-238 |
| *Grampus griseus* | 1 | 1/2 | 63 | -Small (more common in some areas) **(1)**  -Medium (more common in some areas)**(2)**  -Large schools (rare) | -Possibly strong, calves tend to stay longer than non-globicephaliinids dolphins. **(2)**  -Natal philopatry, males move between groups) **(3)** | -Mixed **(2)**  -Maybe some segregation by age **(1)** | 27-28, 136, 234, 239-243 |
| *Peponocephala electra* | 1 | 3 | 257.7 | -Large schools (more common) **(2)** | -Strong (described as ‘strong social bonds’) **(2)** | Mother+calf **(0)**  Unknown **(?)** | 27, 136, 234, 244-246 |
| *Pseudorca crassidens* | 1 | 3 | 36.16 | -Small groups (1)  -Medium (more common in some areas) **(2)**  -Large schools | -Strong (described as ‘strong affiliative behavior’ in stranded animals) **(2)** | -Mixed (based on strandings) **(2)** | 27-28, 45, 136, 247-250 |
| *Orcaella brevirostris (riverine)*  *Orcaella heinsohni (marine)* | 1 | 0 | 3.5 | -Small (most common) **(1)**  -Medium sized groups (rare) (2) | -Weak (described as ‘frequency mixing’ **(1)** | Mother+calf **(0)**  Unknown **(?)** | 251-254 |
| *Orcinus orca* | 1 | 3 | 12 | -Single (mainly males are infrequent) (0)  -Small to Medium (‘fish eating’) **(1)**  -Small (‘mammal eating’) **(1)**  -Large aggregations (3) | -Matrilineal with natal philopatry in fish eating orcas **(3)**  -Two generation matrilineal in mammal eating orcas (3) | -Mixed **(2)** | 27-28, 136, 234, 255*-*266 |

**Species which part of information comes from the sister species

**References**

1. Best PB, Schaeff CM, Reeb D, Palsboll PJ: **Composition and possible function of social groupings of southern right whales in South African waters**. *Behaviour* 2003, **140**:1469-1494.

2. Braham HC, Rice DW: **The Right whale *Balaena glacialis***. *Marine Fisheries Review* 1984, **46**: 38-47.

3. Bannister LJ: 2002. **Baleen Whales (Mysticetes).** In: *Encyclopedia of Marine Mammals* Edited by Perrin WF, Wursig B, Thewissen JGM. San Diego: Academic Press; 2002: 62-73.

4**.** Richardson WJ, Finley KJ, Miller GW, Davis RA, Koski WR: **Feeding social and migration behavior of bowhead whales, *Balaena mysticetus*, in Baffin Bay vs the Beaufort Sea regions with different amounts of human activity.** *Marine Mammal Science* 1995, **11**: 1-45.

5. Landino SW, Treacy SD, Zerwick SA, Dunlap JB: **A large aggregation of bowhead whales (*Balaena mysticetus*) feeding near Point Barrow, Alaska in late October 1992.** *Arctic* 1994, **47**: 232-235.

6. Schilling MR, Seipt I, Weinrich MT, Frohock SE, Kuhlberg AE, Clapham PJ: **Behavior of individually-identified sei whales *Balaenoptera borealis* during an episodic influx into the southern Gulf of Maine in 1986**. *Fishery Bulletin* 1992, 90: 749-755.

7. Lucena A: **Estrutura populacional da *Balaenoptera bonaerensis* (Burmeister) (Cetacea, Balaenopteridae) nas áreas de reprodução do Oceano Atlântico Sul.** *Revista Brasileira de Zoology* 2006, **23**:176-185.

8. O’Callaghan TM, Baker CS: **Summer cetacean community, with particular reference to Bryde’s whales in the Hauraki Gulf, New Zealand**. *Doc Science Internal Series* 55. Wellington, New Zealand: Department of Conservation Press; 2002:18 pp.

9. Tershy BR: **Body Size, Diet, Habitat Use, and Social Behavior of *Balaenoptera* Whales in the Gulf of California**. *Journal of Mammalogy* 1992, **73**: 477-486.

10**.** Berube M, Berchok C, Sears R: **Observation of a male-biased sex ratio in the Gulf of St. Lawrence fin whales (*Balaenoptera physalus*): temporal, geographical, or group structure segragation?** *Marine Mammal Science* 2001, **17**:371-381.

11. Canese S, Cardinali A, Fortuna CM, Giusti M, Lauriano G, Salvati E, Greco S: **The first identified winter feeding ground of fin whales (*Balaenoptera physalus*) in the Mediterranean Sea.** *Journal of Marine Biological Association of the United Kingdom* 2006, **86**: 903-907.

12. Gannier A: **Summer distribution of fin whales (*Balaenoptera physalus*) in the Northwestern Mediterranean Marine Mammals Sanctuary.** *Revue D Ecologie La Terre et la Vie* 2002, **57**:135-150.

13. Panigada S, Di Sciara GN, Panigada MZ: **Fin whales summering in the Pelagos Sanctuary (Mediterranean Sea): Overview of studies on habitat use and diving behaviour**. *Chemistry and Ecology* 2006, **22**: 255-263.

14. Tyack P, Whitehead H: **Male competition in large groups of wintering humpback whales**. *Behaviour* 1982, **83**: 1–23.

15. Mobley JR, Herman LM: **Transience of social affiliation among humpback whales (*Megaptera novaeangliae*) on the Hawaiian wintering grounds**. *Canadian Journal of Zoology* 1985. **63**:762-772.

16. Weinrich MT: **Stable social association among humpback whales (*Megaptera novaeangliae*) in the southern Gulf of Main.** *Canadian Journal of Zoology* 1991, **69**: 3012-3019.

17. Weinrich MT, Rosenbaum H, Baker CS, Blackmer AL, Whitehead H: **The influence of maternal lineages on social affiliations among humpback whales (*Megaptera novaeangliae*) on their feeding grounds in the Southern Gulf of Main.** *Journal of Heredity* 2006, **97**: 226-234.

18. Valsecchi E, Hale P, Corkeron P, Amoss W: **Social structure in migrating humpback whales (*Megaptera novaeangliae*).** *Molecular Ecology* 2002, **11**: 507-518.

19. Clapham PJ, Palsboll PJ, Mattila DK, Vasquez O: **Composition and dynamics of humpback whales competitive groups in the West-Indies**. *Behaviour* 1992, **122**:182-194.

20. Brown MB, Corkeron PJ, Hale PJ, Schultz KW, Bryden MM: **Evidence for a Sex-Segregated Migration in the Humpback Whale (*Megaptera novaeangliae*)**. *Proceedings Biological Sciences* 1995, **259**: 229-234.

21. Straley JM, Herman LM, Jacobsen J: **Evidence of a feeding aggregation of humpback whales (*Megaptera novaeangliae*) around Kodiak Island, Alaska**. *Marine mammal science* 1999, **15**: 210-220.

22. Pomilla C, Rosenbaum HC: **Estimates of relatedness in groups of humpback whales (*Megaptera novaeangliae*) on two wintering grounds of the Southern Hemisphere.** *Molecular Ecology* 2006, **15**: 2541-2555.

23. Urban-Ramirez J, Rojas-Bracho L, Perez-Cortes H, Gomez-Gallardo A, Swartz SL, Ludwig S, Brownell RL: **A review of gray whales (*Eschrichtius robustus*) on their wintering grounds in Mexican waters.** *Journal of Cetacean Research and Management* 2003, **5**: 281–295.

24. Calambokidis J, Darling JD, Deecke V, Gearin P, Gosho M, Megill W, Tombach CM, Goley D, Toropova C, Gisborn B: **Abundance, range, and movements of a feeding aggregation of gray whales (*Eschrichtius robustus*) from California to Southern Alaska in 1998**. *Journal of Cetacean Research and Management* 2002, **4**:267-276.

25. Baird RW: **Sightings of dwarf (*Kogia sima*) and Pygmy (*K. breviceps*) from the main Hawaiian islands**. *Pacific Science* 2005, **59**:461-466.

26. Cardona-Maldonado MA, Mignucci-Giannoni AA: **Pygmy and dwarf sperm whales in Puerto Rico and the Virgin Islands with a review of Kogia in the Caribbean**. *Caribbean Journal of Science* 1999, **35**: 29-37.

27. Acevedo-Guiterrez, A. **Group Behavior**. In: *Encyclopedia of Marine Mammals* Edited by Perrin WF, Wursig B, Thewissen JGM. San Diego: Academic Press; 2002: 537-545.

28. May-Collado LJ, Gerrodete T, Calambokidis J, Rassumen K, Sereg I: **Distribution of Cetaceans sightings in the EEZ of Costa Rica**. *Revista de Biología Tropical* 2005, 53:249-263.

29. Caldwell DK, Caldwell MC: **Pygmy sperm whale *Kogia breviceps* (de Blainville 1838) and dwarf sperm whale *Kogia simus* (Owen 1866)**. In: *Handbook of Marine Mammals* Edited by Ridgway SH, Harrison Sir H. New York: Academic Press; 1989: 235-260 [vol. 4.].

30. McAlpine DF: **Pygm and Dwarf sperm whales (*Kogia breviceps* and *K. sima*)**. In: *Encyclopedia of Marine Mammals* Edited by Perrin WF, Wursig B, Thewissen JGM. San Diego: Academic Press; 2002: 1007-1009.

31. Nagorsen DW, Stewart GE: **A dwarf sperm whale (*Kogia simus*) from the Pacific coat of Canada**. *Journal of Mammalogy* 1983, **64**:505-506.

32. Christal J, Whitehead H, Lettevall E: **Sperm whale social units: variation and change**. *Canadian Journal of Zoology* 1998, **76:** 1431–1440.

33. Mesnick SL: **Genetic relatedness in sperm whales: evidence and cultural implications**. *Behavioral and Brain Science* 2001, **24:** 346–347.

34. Lettevall E, Richter C, Jaquet N, Slooten E, Dawson S, Whitehead H, Christal J, McCall Howard P: **Social structure and residency in aggregatins of male sperm whales.** *Canadian Journal of Zoology* 2002, **80:** 1189-1196.

35. Rendell LE, Whitehead HL: **Do sperm whales share coda vocalizations? Insights into coda usage from acoustic size measurement**. *Animal Behaviour* 2004, **67:** 865-874.

36. Whitehead H: **Baby-sitting, dive synchrony, and indications of alloparental care in sperm whales**. *Behavioral Ecology and Sociobiology* 1996, **38:** 237–244.

37. Whitehead H: **Sperm whales (*Physeter macrocephalus*)**. In: *Encyclopedia of Marine Mammals* Edited by Perrin WF, Wursig B, Thewissen JGM. San Diego: Academic Press; 2002:1165-1171.

38. Whitehead H, Weilgart LS: **Patterns of visually observable behaviour and vocalizations in groups of female sperm whales**. *Behaviour* 1991, **118:** 275-296.

39. Whitehead H, Weilgart LS: **The sperm whale: social females and roving males.** *In Cetacean societies: field studies of dolphins and whale* Edited by Mann J, Connor RC, Tyack PL, Whitehead H. Chicago: University of Chicago Press; 2000: 154–172.

40. Whitehead H, Waters S, Lyrholm T: **Social organization in female sperm whales and their offspring: constant companions and casual acquaintances**. *Behavioral Ecology and Sociobiology* 1991, **29:** 385–389.

41. Kasuya T: **Distribution and behavior of Baird’s beake whales off the Pacific coast of Japan.** *The Scientific Report of Whales Research Institute* 1986, **37:** 61-83.

42. Kasuya T, Brownell RLJr, Balcomb III KC: **Life history of Baird's beaked whales off the Pacific coast of Japan.** *Reports of the International Whaling Commission* 1997, **47:** 969-979.

43. Balcomb KC: **Baird’s beaked whale *Berardius baiirdi* Stejneger,1883: Arnoux’s beaked whale *Berardius arnuxii* Duvernoy, 1851**. In: *Handbook of Marine Mammals* Edited by Ridgway SH, Harrison Sir H. New York: Academic Press; 1989: 261-288. [vol. 4].

44. Aurioles-Gamboa D: **Notes on a mass stranding of Baird beaked-whales in the Gulf of California, Meximo**. *California Fish and Game* 1992, **78**:116-123.

45. Connor RC, Mann J, Tyack PL, Whitehead H: **Social evolution in toothed whales**. *Trends in Ecology and Evolution* 1998, **13:** 228–232.

46. Dawson S, Barlow J, Ljungblad D: **Sounds recorded from Baird’s beaked whale, *Berardius bairdii*.** *Marine Mammal Science* 1998, **14:** 335-344.

47. Ponganis PJ, Kooyman GL: **Multiple sightings of Arnoux’s beaked whales along the Victoria land coast.** *Marine Mammal Science* 1995, **11**: 247-250.

48. Rogers TL, Brown SM: **Acoustic observations of Arnoux’s beaked whale (*Berardius arnuxii*) off Kemp Land, Antarctica**. *Marine Mammal Science* 1999, **15**:192-198.

49. Hooker SK, Whitehead H, Gowans S, Baird RW: **Fluctuations in distribution and patterns of individual range use of northern bottlenose whales**. *Marine Ecology Progress Series* 2002, **225**:287-297.

50. Gowans S, Rendell L: **Head-butting in northern bottlenose whales (*Hyperoodon ampullatus*): a possible function for big heads?** *Marine Mammal Science* 1999, **15**:1342-1350.

51. Gowans S, Whitehead H, Hooker SK: **Social organization in northern bottlenose whales, *Hyperoodon ampullatus*: not driven by deep-water foraging?** *Animal Behaviour* 2001, **62**:369-377.

52. Kasamatsu F, Joyce GD: **Current status of odontocetes in the Antarctic**. *Antarctic Science* 1995, **7**: 365-379.

53. Hooker SK, Baird RW. [**Observations of Sowerby's Beaked Whales, *Mesoplodon bidens,* in the Gully, Nova Scotia**](http://apps.isiknowledge.com.ezproxy.fiu.edu/WoS/CIW.cgi?SID=L1FAnEGHoJfBk4b2B3G&Func=Abstract&doc=8/7). *Canadian Field-Naturalist* 1999, **113**: 273-277.

54. Pitman RL: 2002. **Mesoplodon Whales (*Mesoplodon* sp).** In: *Encyclopedia of Marine Mammals* Edited by Perrin WF, Wursig B, Thewissen JGM. San Diego: Academic Press; 2002: 738-742.

55. Mead JG: **Beaked whales of the genus *Mesoplodon***. In: *Handbook of Marine Mammals* Edited by Ridgway SH, Harrison Sir H. New York: Academic Press; 1989: 349-415. [vol. 4.].

56. Baird RW, McSweeney DJ, Ligon AD, Webster DL: **Tagging feasibility and diving of Cuvier’s beaked whales (*Ziphius cavirostris*) and Blainville’s beaked whales (*Mesoplodon densirostris*) in Hawaii**. *Report to the Wildlife Fund, Volcano, HI. Order No. AB133F-03-SE-0986. SWFSC, NMFS, La Jolla CA* 2004.

57. Borsa P: [**Marine mammal strandings in the New Caledonia region, Southwest Pacific**](http://apps.isiknowledge.com.ezproxy.fiu.edu/WoS/CIW.cgi?SID=L1FAnEGHoJfBk4b2B3G&Func=Abstract&doc=11/3)**.** *Comptes Rendus Biologies* 2006, **329**: 277-288.

58. MacLeod CD, Zuur AF: [**Habitat utilization by Blainville's beaked whales off Great Abaco, northern Bahamas, in relation to seabed topography**](http://apps.isiknowledge.com.ezproxy.fiu.edu/WoS/CIW.cgi?SID=L1FAnEGHoJfBk4b2B3G&Func=Abstract&doc=11/6). *Marine Biology* 2005, **147**: 1-11.

59. Baird RW, Webster DL, McSweeney DJ, Ligon AD, Schorr GS: **Diving behavior and ecology of Cuvier’s (*Ziphius cavirostris*) and Blainville’s beaked whales (*Mesoplodon densirostris*) in Hawaii. Report to Cascadia Research Collective, WA**. *Order No. AB133F-04-RQ-0928. SWFSC, NMFS, La Jolla CA* 2005.

60. Gomercic H, Gomercic MD, Gomercic T, Lucic H, Dalebout M, Galov A, Skrtic D, Curkovic S, Vukovic S, Huber D: **Biological aspects of Cuvier's beaked whale (*Ziphius cavirostris*) recorded in the Croatian part of the Adriatic Sea.** *European Journal of Wildlife Research* 2006, **52**: 182-187

61. Marini L, Consiglio C, Angradi AM, Catalano B, Sanna A, Valentini T:

**Distribution, abundance and seasonality of cetaceans sighted during scheduled ferry crossings in the central Tyrrhenian Sea: 1989-1992**. *Italian Journal of Zoology* 1996, **63**: 381-388.

62. Heyning JE: **Cuvier’s Beaked Whale *Ziphius cavirostris* G. Cuvier, 1823**. In: *Handbook of Marine Mammals* Edited by Ridgway SH, Harrison Sir H. New York: Academic Press; 1989: 289-308. [vol.4]

63. Akbar M, Mehal QM, Arshed MJ: **Population estimation of Indus dolphin from Jinnah-Guddu Barrage**. *Journal of Applied Sciences* 2004, **4**:21-23.

64. Kasuya T, Haque AKM: **Some informations on distributions and seasonal movement of the Ganges Dolphin.** *The Scientific Report of Whales Research Institute* 1972, **24:** 109-115.

65. Reeves RR, Brownell RLJr: **Susu, *Platanista gangetica* (Roxburg 1801) and *Platanista minor* (Owen 1853).** In: *Handbook of Marine Mammals* Edited by Ridgway SH, Harrison Sir H. New York: Academic Press; 1989: 69-99. [vol.4].

66. Braulik GT: **Status assessment of the Indus river dolphin, *Platanista gangetica minor*, March-April 2001**. *Biological Conservation* 2006, **129**:579-590.

67. Smith BD: 1990 **Status and conservation of the Ganges river dolphin *Platanista gangetica* in the Karnali River, Nepal**. *Biological Conservation* 1993, **66**:159-169.

68. Smith BD: **Susu and Bhulan (*Platanista gangetica gangetica* and *P.g.minor*).** In: *Encyclopedia of Marine Mammals* Edited by Perrin WF, Wursig B, Thewissen JGM. San Diego: Academic Press; 2002:1208-1213.

69. Smith BD, Braulik G, Strindberg S, Ahmed B, Mansur R: **Abundance of Irrawady dolphins (*Orcaella brevirostris*) and Ganges River dolphins (*Platanista gangetica gangetica*) estimated using concurrent counts made by independent teams in waterways of the sundarbans mangrove forest in Bangladesh**. *Marine Mammal Science* 2006, **22**: 527-547.

70. Smith BD, Ahmed B, Ali ME, Braulik G: **Status of the Ganges river dolphin or shushuk *Platanista gangetica* in Kaptai Lake and southern rivers of Bangladesh**. *Oryx* 2001, **35**: 61-72.

71. Reeves RR: **River dolphins**. In: *Encyclopedia of Marine Mammals* Edited by Perrin WF, Wursig B, Thewissen JGM. San Diego: Academic Press; 2002:1039–1242.

72. Best RC, da Silva VMF: **Amazon River Dolphin, Boto *Inia geoffrensis* (de Blainville 1817)**. In: *Handbook of Marine Mammals* Edited by Ridgway SH, Harrison Sir H. New York: Academic Press; 1989: 1-23. [vol.4].

73. Aliaga-Rossel E: **Distribution and abundance of the river dolphin (*Inia geoffrensis*) in the Tijamuchi River, Beni, Bolivia**. *Aquatic Mammals* 2002, **28:** 312-323.

74. Da Silva VMF: **Amazon River Dolphin (*Inia geoffrensis*).** In: *Encyclopedia of Marine Mammals* Edited by Perrin WF, Wursig B, Thewissen JGM. San Diego: Academic Press; 2002: 18-20.

75. Martin AR, da Silva VMF: **Sexual dimorphism and body scarring in the boto (Amazon River dolphin) *Inia geoffrensis***. *Marine Mammals* *Science* 2006, **22:** 25–33.

76. McGuire TL, Winemiller KO: **Ocurrence patterns, habitat associations, and potential prey of the river dolphin, *Inia geoffrensis*, in the Cinaruco River, Venezuela**. *Biotropica* 1998, **30:** 625-638.

77. Muizon de C: **River dolphins evolutionary history**. In: *Encyclopedia of Marine Mammals* Edited by Perrin WF, Wursig B, Thewissen JGM. San Diego: Academic Press; 2002: 1043-1050.

78. Trujillo FG: **Habitat use and social behaviour of the freshwater dolphin *Inia geoffrensis* (de Blainville 1817) in the Amazon and Orinoco basins**. *Ph.D. Thesis*, University of Aberdeen, Scotland 2000: 138 pp.

79. Brownell RLJr: **Franciscana, *Pontoporia blainvillei* (Gervais and d’Orbigny 1844).** In: *Handbook of Marine Mammals* Edited by Ridgway SH, Harrison Sir H. New York: Academic Press; 1989: 45-67. [vol.4].

80. Cremer MJ, Simoes-Lopes PC: **The occurrence of *Pontoporia blainvillei* (Gervais & d’Orbigny)(Cetacea, Pontoporidae) in an estuarine area in southern Brazil**. *Revista Brasileira de Zoologia* 2005, **22**: 717-723.

81. Crespo EA: **Franciscana (*Pontoporia blainvillei*).** In: *Encyclopedia of Marine Mammals* Edited by Perrin WF, Wursig B, Thewissen JGM. San Diego: Academic Press; 2002: 482-484.

82. Danilewicz D, Claver JA, Perez Carrera AL, Secchi ER, Fontoura NF: **Reproductive biology of male Franciscanas from Rio Grande do Sul, southern Brazil.** *Fishery Bulletin* 2004, **102:** 581-592.

83. Kaiya Z: **Baiji (*Lipotes vexillifer*).** In: *Encyclopedia of Marine Mammals* Edited by Perrin WF, Wursig B, Thewissen JGM. San Diego: Academic Press; 2002: 58-61.

84. Pexiun C: **Baiji, *Lipotes vexillifer* (Miller 1918).** In: *Handbook of Marine Mammals* Edited by Ridgway SH, Harrison Sir H. New York: Academic Press; 1989: 25-43. [vol.4].

85. Zhang X, Wang D, Liu R, Wei Z, Hua Y, Wang Y, Chen Z, Wang L: **The Yangtze River dolphin or baiji (*Lipotes vexillifer*): population status and conservation issues in the Yangtze River, China.** *Aquatic Conservation: Marine and Freshwater Ecosystems* 2003, **13**: 51-64.

86. Brownell JRJr, Herald ES: ***Lipotes vexillifer***. *Mammalian Species* 1972, **10**: 1-4.

87. Brownell RLJr, Clapham RJ: **Spectacle Porpoise *Phocoena dioptrica* (Lahille, 1912).** In: *Handbook of Marine Mammals* Edited by Ridgway SH, Harrison Sir H. New York: Academic Press; 1999: 379-393. [vol.6].

88. Goodall RNP: **Spectacled Porpoise (*Phocoena dioptrica*).** In: *Encyclopedia of Marine Mammals* Edited by Perrin WF, Wursig B, Thewissen JGM. San Diego: Academic Press; 2002: 1158-1161.

89. Goodall RNP, Schiavini ACM: **On the biology of the spectacled porpoise, *Australophocoena dioptrica***. *International Whaling Commision* 1995, Special Issue **16:** 411-453.

90. Culik BM, Koschinski S, Tregenza N, Ellis GM: **Reactions of harbor porpoises *Phocoena phocoena* and herring *Clupea harengus* to acoustic alarms.** *Marine Ecology Progress Series* 2001, **211**:255-260.

91. Bjφrge A, Tolley KA: **Harbor porpoise (*Phocoena phocoena*).** In: *Encyclopedia of Marine Mammals* Edited by Perrin WF, Wursig B, Thewissen JGM. San Diego: Academic Press; 2002: 549-551.

92. Hoek W: **An unusual aggregation of Harbor porpoises (*Phocoena phocoena*).** *Marine Mammal Science* 1992, **8**:152-155.

93. Read, A. J. **Harbor porpoise *Phocoena phocoena* (Linnaeus, 1758).** In: *Handbook of Marine Mammals* Edited by Ridgway SH, Harrison Sir H. New York: Academic Press; 1999: 323-355. [vol.6].

94. Read AJ, Westgate AJ: **Monitoring the movements of harbour porpoises (*Phocoena phocoena*) with satellite telemetry**. *Marine Biology* 1997, **130**: 315-322.

95. Jaramillo-Legorreta AM, Rojas-Bracho L, Gerrodette T: **A new abundance estimate for vaquitas. First step for recovery**. *Marine Mammal Science* 1999, **15:** 957-973.

96. Rojas-Brucho L, Jaramillo-Lagorreta A: **Vaquita (*Phocoena sinus).*** In: *Encyclopedia of Marine Mammals* Edited by Perrin WF, Wursig B, Thewissen JGM. San Diego: Academic Press; 2002: 1277-1280.

97. Vidal O, Brownell RL, Findley LT: **Vaquita - *Phocoena sinus* (Norris and McFarland, 1958).** In: *Handbook of Marine Mammals* Edited by Ridgway SH, Harrison Sir H. New York: Academic Press; 1999: 357-378. [vol.4].

98. Jefferson TA: ***Phocoenoides dalli****.* *Mammalian Species* 1988, **319:** 1-7.

99. Jefferson TA: **Dall’s Porpoise (*Phocoenoides dalli*).** In: *Encyclopedia of Marine Mammals* Edited by Perrin WF, Wursig B, Thewissen JGM. San Diego: Academic Press; 2002: 308-310.

100. Jefferson TA, Hung SK: ***Neophocaena phocaenoides***. *Mammalian Species* 2004, **746:** 1-12.

101. Amano M. 2002. **Finless Porpoise (*Neophocaena phocaenoides*).** In: *Encyclopedia of Marine Mammals* Edited by Perrin WF, Wursig B, Thewissen JGM. San Diego: Academic Press; 2002: 432-435.

102. Kasuya T: ***Neophocaena phocaenoides* (Cuvier, 1829).** In: *Handbook of Marine Mammals* Edited by Ridgway SH, Harrison Sir H. New York: Academic Press; 1999: 411-442. [vol.6].

103. Wang K, Wang D, Akamatsu T, Li S, Xiao J: **A passive acoustic monitoring method applied to observation and group size estimation of finless porpoises**. *Journal of the Acoustical Society of America* 2005, **118**: 1180-1185.

104. Wei ZD, Wang D, Kuang X, Wang K, Wang X, Xiao J, Zhao Q, Zhang X: **Observations on behavior and ecology of the Yangtze finless porpoise (*Neophocaena phocaenoides asiaeorientalis*) group at Tian-e-Zhou Oxbow of the Yangtze River**. *Raffles Bulletin of Zoology* 2002, **Suppl** **10**:97–103.

105. Hay KA, Mansfield AW: **Narwhal, *Monodon monocerus* (Linnaeus 1758).** In: *Handbook of Marine Mammals* Edited by Ridgway SH, Harrison Sir H. New York: Academic Press; 1989: 145-176. [vol.4].

106. Heide-Jørgensen MP: **Narwhal (*Monodon monocerus*).** In: *Encyclopedia of Marine Mammals* Edited by Perrin WF, Wursig B, Thewissen JGM. San Diego: Academic Press; 2002: 783-787.

107. Cosens SE, Dueck LP: **Group size and activity patterns of belugas (*Delphinapterus leucas)* and narwhals (*Monodon moncerus*) during spring migration in Lancaster Sound**. *Canadian Journal of Zoology* 1991, 6:1630-1635.

108. Pallsboll PJ, Heide-Jorgensen MP, Dietz R: **Population structure and seasonal movements of narwhals, *Monodos monocerus*, determined from mtDNA analysis**. *Heredity* 1997, **78**: 284-292.

109. Silverman HB: **Social organization and behaviour of the narwhal, *Monodon monoceros* L. in Lancaster Sound, Pond Inlet and Tremblay Sound, Northwest Territories**. *M.Sc. Thesis*, McGill University, Montreal, Quebec; 1979.

110. Reeves RR, Tracey S: ***Monodon monocerus***. *Mammalian Species* 1980, **127**:1-7.

111. Brodie PF: **White whale, *Delphinapterus leucas* (Pallas 1776)**. In: *Handbook of Marine Mammals* Edited by Ridgway SH, Harrison Sir H. New York: Academic Press; 1989: 119-141. [vol.4].

112. Bel’kovitch VM, Kirillova OI: **Investigation of white whales (*Delphinapterus leucas*) biology in reproductive period**. *Zoologichesky Zhurnal* 2000, **79**:89-96.

113. Litovka DI: **Distribution of the Beluga *Delphinapterus leucas* in the Anadyr Estuary in 2000**. *Russian Journal of Marine Biology* 2002, **28**: 263-266.

114. Lønø O, Øynes P: **White whale fishery at Spitzbergen.** *Norway Whaling Gaz* 1961, **50:** 267–286.

115. Lukin LR, Vasil’ev LY: **Distribution of white whales (*Delphinapterus leucas*) in the White Sea and the Southeastern region of the Barents Sea during the ice season.** *Russian Journal of Marine Biology* 2004, **35**:236-241.

116. Lydersen C, Martin AR, Kovacs KM, Gjertz I: **Summer and autumn movements of white whales *Delphinapterus leucas* in Svalbard, Norway.** *Marine Ecology Progress Series* 2001, **219:** 265-274.

117. Michaud R: **Social organization of the St. Lawrence beluga whale**. *13th Biennial Conference Biology Marine Mammals:* 28 Nov–3 Dec 1999; Wailea, Maui, Hawaiii.

118. Richard PR, Martin AR, Orr JR: **Summer and autumn movements of belugas of the Eastern Beaufort Sea stock**. *Arctic* 2001, **54:** 223–236.

119. Smith TG, Hammill MO, Martin AR: **Herd composition and behaviour of white whales (*Delphinapterus leucas*) in two Canadian arctic estuaries**. *Meddelelser om Grønland, Bioscience* 1994, **39:** 175–184.

120. Dawson SM: ***Cephalorhynchus* dolphins**. In: *Encyclopedia of Marine Mammals* Edited by Perrin WF, Wursig B, Thewissen JGM. San Diego: Academic Press; 2002: 200-204.

121. Lescrauwaet AC, Gibbons J, Guzman L, Schiavini A: **Abundance estimation of Commerson’s dolphin in the eastern area of the Strait of Magalla-Chile**. *Revista Chilena de Historia Natural* 2000, **73**: 473-478.

122. Goodall RNP: **Chilean dolphins *Cephalorhynchus eutropia* (Gray, 1846).** In: *Handbook of Marine Mammals* Edited by Ridgway SH, Harrison Sir H. New York: Academic Press; 1994: 269-287. [vol.5].

123. Bräger S: **Association patterns in three populations of Hector’s**

**dolphin, *Cephalorhynchus hectori***. *Canadian Journal of Zoology* 1999, **77**:13–18

124. Slooten E, Dawson SM: **Hector’s dolphin Cephalorhynchus hectori (van Beneden, 1881)**. In: *Handbook of Marine Mammals* Edited by Ridgway SH, Harrison Sir H. New York: Academic Press; 1994: 311–333. [vol.5].

125. Slooten E: **Behaviour of Hectorís dolphins: classifying behaviour by**

**sequence analysis**. *Journal of Mammalogy* 1994, **75**: 956-964.

126. Slooten E: **Population Biology, Social Organisation and Behaviour of**

**Hectorís Dolphin**. PhD thesis. University of Canterbury, Christchurch; 1990.

127. Slooten E, Dawson SM, Whitehead H: **Associations among photographically identified Hectorís dolphins**. *Canadian Journal of Zoology* 1993, **71**: 2311-2318.

128. Best PB, Abernethy RB: **Heavyside's dolphin *Cephalorhynchus heavisisdii* (Gray, 1828).** In: *Handbook of Marine Mammals* Edited by Ridgway SH, Harrison Sir H. New York: Academic Press; 1994:289-310. [vol.5].

129. Brownell RLJr, Crespo EA, Donahue MA: ***Lagenorhynchus australis* (Peale, 1848).** In: *Handbook of Marine Mammals* Edited by Ridgway SH, Harrison Sir H. New York: Academic Press; 1999: 105-120. [vol.6].

130. Viddi FA, Lescrauwaet AK: **Insights on habitat selection and behavioural patterns of Peale’s dolphins (*Lagenorhynchus australis*) in the Strait of Magellan, Southern Chile**. *Aquatic Mammals* 2005, **31**:176-183.

131. Goodall RN: **Peale’s Dolphin (*Lagenorhynchus australis*).** In: *Encyclopedia of Marine Mammals* Edited by Perrin WF, Wursig B, Thewissen JGM. San Diego: Academic Press; 2002: 890-894.

132. Goodall RN: **Hourglass Dolphin (*Lagenorhynchus cruciger*).** In: *Encyclopedia of Marine Mammals* Edited by Perrin WF, Wursig B, Thewissen JGM. San Diego: Academic Press; 2002: 583-585.

133. Brownell RLJr, Donahue MA: ***Lagenorhynchus cruciger* (Quoy and Gaimard, 1824).** In: *Handbook of Marine Mammals* Edited by Ridgway SH, Harrison Sir H. New York: Academic Press; 1999: 121-137. [vol.4].

134. Van Waerebeek K, Würsig B: **Pacific white-sided dolphin and dusky dolphins.** In: *Encyclopedia of Marine Mammals* Edited by Perrin WF, Wursig B, Thewissen JGM. San Diego: Academic Press; 2002: 859-861.

135. Morton A: **Occurrence, photo-identification and prey of Pacific White-sided dolphins (*Lagenorhynchus obliquidens*) in the Broughton Archipelago, Canada 1984-1998**. *Marine Mammal Science* 2000, **16**:80-93.

136. Ferguson MC, Barlow J, Fiedler P, Reilly SB, Gerrodette T: **Spatial models of delphinids (family Delphinidae) encouter rate and group size in the eastern tropical Pacific Ocean**. *Ecological Modelling* 2006, **193**:645-662.

137. Markowitz TM: **Social organization of the New Zealand dusky dolphin.** *Ph.D. Thesis***. Texas A&M University**, 2004. 255 pp.

138. Brownell, RLJr, Cipriano F: ***Lagenorhynchus obscurus* (Gray, 1828).** In: *Handbook of Marine Mammals* Edited by Ridgway SH, Harrison Sir H. New York: Academic Press; 1999: 85-104. [vol.6].

139. Würsig B, Würsig M: **Behavior and ecology of the dusky dolphins, *Lagenorhynchus obscurus*, in the South Atlantic**. *Fishery Bulletin* 1980, **77:** 871-890.

140. Würsig B, Bastida R: **Long-range movement and individual association of two dusky dolphins (*Lagenorhynchus obscurus*) off Argentina**. *Journal of Mammalogy* 1986, **67:** 773-774.

141.[Würsig](http://ecol.zool.kyoto-u.ac.jp/~chaka/DPSE/bionote.htm#wursig) B, Pearson H, Markowitz T: **Delphinid Behavioral Flexibilities: Social Strategies in Dusky Dolphins*.*** *Kyoto Conference. Delphinid and Primate Social Ecology: A Comparative Discussion:* July 29 – 30 2005; Kyoto, Japan.

142. Cipriano F: **Atlantic White-Sided Dolphin (*Lagenorhynchus acutus*).** In: *Encyclopedia of Marine Mammals* Edited by Perrin WF, Wursig B, Thewissen JGM. San Diego: Academic Press; 2002: 49-50.

143. Reeves RR, Smeenk C, Brownell RLJr, Kinze CC: **Atlantic white-sided dolphin-*Lagenorhynchus acutus* (Gray 1828).** In: *Handbook of Marine Mammals* Edited by Ridgway SH, Harrison Sir H. New York: Academic Press; 1999: 31-57. [vol. 6].

144. Sergeant DE, Aubin DJ, Gerarci JR: **Life history and northwest Atlantic status of the Atlantic white-sided dolphin, *Lagenorhynchus acutus***. *Cetology* 1980, **37:** 1-12

145. Leatherwood S, Walker WA: **The northern right whale dolphin *Lissodelphis borealis* Peale in the eastern North Pacific**. In *Behavior of marine mammals* Edited by Winn HE, Olla BL. New York: Plenum Press; 1979: 85-141. [vol.3].

146. Jefferson TA, Newcomer MW, Leatherwood S, Van Waebebeek K: **Righ whale dolphins-*Lissodelphis borealis* (Peale 1848) and *Lissodelphis peronni* (Lacepede, 1804).** In: *Handbook of Marine Mammals* Edited by Ridgway SH, Harrison Sir H. New York: Academic Press; 1994:335-363. [vol.5].

147. Jefferson TA, Newcomer MW: ***Lissodelphis borealis****. Mammalian Species* 1993, **425:** 1-6.

148. Cruickshank RA, Brown SG: **Recent observations and some historical records of southern right-whale dolphins *Lissodelphis peronii***. *Fisheries Bulletin (South Africa)* 1981, **15:** 109-121.

149. Newcomer MW, Jefferson TA, Brownell RLJr: ***Lissodelphis peronii***. *Mammalian Species* 1996, **531:** 1-5.

150. Van Waerebeek KJ, Canto J, Gonzalez J, Oporto J, Brito JL: **Southern right whale dolphins, *Lissodelphis peronii* off the Pacific coast of South America**. *Zeitschrif für Säugetierkunde* 1991, **56**:284-295.

151. Perrin WF: **Common Dolphins (*Delphinus delphis*).** In: *Encyclopedia of Marine Mammals* Edited by Perrin WF, Wursig B, Thewissen JGM. San Diego: Academic Press; 2002: 245-248.

152. Bearzi G, Reeves RR, Notarbartolo-Di-Sciara G, Politi E, Cañadas A, Frantzis A, Mussi B: **Ecology, status and conservation of short-beaked common dolphins Delphinus delphis in the Mediterranean Sea**. *Mammal Review* 2003, *33*:224-252.

153. Evans WE: **Common dolphin, white-bellied porpoise *Delphinus delphis* Linnaeus, 1758.** In: *Handbook of Marine Mammals* Edited by Ridgway SH, Harrison Sir H, vol. 5. New York: Academic Press; 1994:191–224.

154. Bruno S: **Il metodo della fotoidentificazione applicato allo studio della socio-ecologia di delfini comuni (Delphinus delphis) nel Mar Ionio orientale**, Degree in Biological Sciences Thesis. University of Padova, Italy 2001.

155. Bruno S, Politi E, Bearzi G: **Social organisation of a common dolphin community in the eastern Ionian Sea: evidence of a fluid fission-fusion society**. *European Research on Cetaceans* 2004, **15**.

156. Perrin WF, Hohn AA: **Pantropical spotted dolphin *Stenella attenuata* (Gray 1846).** In: *Handbook of Marine Mammals* Edited by Ridgway SH, Harrison Sir H. New York: Academic Press; 1994: 71–98. [vol. 5.]

157. Perrin WF: **Stenella *attenuata***. *Mammalian Species* 2001, **683:** 1-8.

158. Perrin WF: **Pantropical spotted dolphins (*Stenella attenuata*).** In: *Encyclopedia of Marine Mammals* Edited by Perrin WF, Wursig B, Thewissen JGM. San Diego: Academic Press; 2002: 865-867.

159. Pryor K, Shallenberger IK: **Social structure in spotted dolphins (*Stenella attenuata*) in the tuna purse seine fishery in the eastern tropical Pacific**. In: *On behavior: essays and research* Edity by K. Pryor. Washington DC: Sunshine Books; 1995: 135–172.

160. May-Collado LJ, Morales-Ramirez A: **Presencia y patrones de comportamiento del delfín manchado costero, *Stenella attenuata graffmani* (Cetacea: Delphinidae) en el Golfo de Papagayo, Costa Rica.** *Revista de Biología Tropical* 2005, **53:** 265-276

161. Kasuya T: **Reconsidertion of life history parameters of the spotted and striped dolphins based on cemental layers**. *The Scientific Report of Whales Research Institute* 1976, **28:** 73-106.

162. Kasuya T: **Overview of cetacean life histories: an essay in their evolution**. In: *Whales, seals, fish and man* Edited by AS Blix, Walløe L, Ulltang Ø. Netherlands: Elsevier Science; 1995: 481–497.

163. Perrin WF, Mead JG: **Clymene Dolphin, *Stenella clymene* (Gray, 1828).** In: *Handbook of Marine Mammals* Edited by Ridgway SH, Harrison Sir H. New York: Academic Press; 1994: 161-172. [vol.5].

164. Mullin KD, Higgins LV, Jefferson TA, Hansen LJ: **Sightings of the Clymene dolphin (*Stenella clymene*) in the Gulf of Mexico.** *Marine Mammal Science* 1994, **10:** 464–470.

165. Jefferson TA, Curry BE: ***Stenella clymene***. *Mammalian Species* 2003, **726:** 1-5.

166. Jefferson TA: **Clymene Dolphin (*Stenella clymene*).** In: *Encyclopedia of Marine Mammals* Edited by Perrin WF, Wursig B, Thewissen JGM. San Diego: Academic Press; 2002: 234-236.

167. Jefferson TA, Odell DK, Prunier KT: **Notes on the biology of the Clymene dolphin (*Stenella clymene*) in the northern Gulf of Mexico**. *Marine Mammal Science* 1995, **11**:564– 573.

168. Herzing DL: **Vocalizations and associated underwater behavior of free-ranging Atlantic spotted dolphins, *Stenella frontalis*, and bottlenose dolphins, *Tursiops truncatus*.** *Aquatic Mammals* 1996, **22:** 61–79.

169. Herzing DL: **The natural history of free-ranging Atlantic spotted dolphins (*Stenella frontalis*): age classes, color phases, and female reproduction**. *Marine Mammals Science* 1997, **13:** 40-59.

170. Herzing DL: **Acoustics and social behavior of wild dolphins: implications for a sound society.** In *Hearing by whales and dolphins* Edited by Au WWL, Popper AN, Fay RE. New York: Springer Press; 2000: 225-272.

171. Perrin WF: **Atlantic spotted dolphin (*Stenella frontalis*).** In: *Encyclopedia of Marine Mammals* Edited by Perrin WF, Wursig B, Thewissen JGM. San Diego: Academic Press; 2002: 47-49.

172. Perrin WF, Caldwell DK, Caldwell MC: **Atlantic spotted dolphin *Stenella frontalis* (G. Cuvier, 1829).** In: *Handbook of Marine Mammals* Edited by Ridgway SH, Harrison Sir H. New York: Academic Press; 1994: 173–190. [vol.5].

173. Perrin WF: ***Stenella frontalis***. *Mammalian Species* 2002, **702:** 1-6.

174. Archer FI, Perrin WF: ***Stenella coeruleoalba***. *Mammalian Species* 1999, **603**:1-9.

175. Perrin WF, Wilson CE, Archer FI: **Striped dolphin. *Stenella coeruleoalba* (Meyen, 1833).** In: *Handbook of Marine Mammals* Edited by Ridgway SH, Harrison Sir H. New York: Academic Press; 1994:129-159.[vol.5].

176. Perrin WF: **Spinner dolphin (*Stenella longirostris*).** In: *Encyclopedia of Marine Mammals* Edited by Perrin WF, Wursig B, Thewissen JGM. San Diego: Academic Press; 2002: 1174-1178.

177. Perrin WF, Gilpatrick JW: **Spinner dolphin, *Stenella longirostris* (Gray, 1828).** In: *Handbook of Marine Mammals* Edited by Ridgway SH, Harrison Sir H. New York: Academic Press; 1994:99–128. [vol.5].

178. Perrin WF: ***Stenella longirostris***. *Mammalian Species* 1998, **599:** 1–7.

179. Würsig B, Wells RS, Würsig M, Norris KS: **Population structure**. In: *The Hawaiian spinner dolphin* First Edition Edited by Norris KS, Würsig B, Wells RS, Würsig M. Berkeley: University of California Press; 1994: 122–140.

180. Norris KS, Wursig B, Wells RS, Würsig M: **The Hawaiian Spinner dolphin***.* Berkeley: University of California Press; 1994.

181. Karczmarski L, Würsig B, Gailey G, Larson KW, Vanderlip C: **Spinner dolphins in remote Hawaiian atoll: social grouping and population structure.** *Behavioral Ecology and Sociobiology* 2005, **16**:675-685.

182. Wells RS: **The role of long-term study in understanding the social structure of a bottlenose dolphin community***.* In*: Dolphin societies: discoveries and puzzles,* First Edition Edited by Pryor K, Norris KS. Berkeley: University of California Press; 1991: 199–225.

183. Wells RS, Scott MD, Irvine AB: **The social structure of free-ranging bottlenose dolphins**. *Current Mammalogy* 1987, **1:** 247–305

184. Wells RS: **Dolphin social complexity: lessons from long-term study and life history**. In: *Animal social complexity: intelligence, culture, and individualized societie*s, First Edition Edited by Waal FBM, Tyack PL. Cambridge: Harvard University Press; 2003: 32–56.

185. Würsig B: **Occurrence and group organization of Atlantic bottlenose porpoises (*Tursiops truncatus*) in an Argentine bay**. *Biological Bulletin* 1978, **154:** 348–359

186. Defran RH, Weller DW: **The occurrence, distribution, and site fidelity of bottlenose dolphins (*Tursiops truncatus*) in San Diego, California**. *Marine Mammal Science* 1999, **15:** 366–380.

187. Defran RH, Weller DW, Kelly DL, Espinoza MA: **Range characteristics of Pacific bottlenose dolphins within the Southern California Bight**. *Marine Mammal Science* 1999, **15:** 381–393.

188. Connor RC, Smolker RA, Richards AF: **Dolphin alliances and coalitions.** In: *Coalitions and Alliances in Humans and* *Other Animals* Edited by Harcourt AH, de Waal FBM. Oxford: Oxford University Press; 1992: 415–443.

189. Smolker RA, Richards AF, Connor RC, Pepper JW: **Sex differences in patterns of association among Indian Ocean bottlenose dolphins.** *Behaviour* 1992, **123:** 38–69

190. Smolker R, Pepper JW: **Whistle convergence among allied male bottlenose dolphins (Delphinidae, *Tursiops* *spp*.).** *Ethology* 1999, **105:** 595–617.

191. Smolker R, Mann J, Smuts B: **Use of signature whistles during separations and reunions between bottlenose dolphin mothers and infants**. *Behavioral Ecological Sociobiology* 1993, **33:** 393–402.

192. Samuels A, Gifford T: **A quantitative assessment of dominance relations among bottlenose dolphins.** *Marine Mammal Science* 1997, **13:** 70-99.

193. Parsons KM, Durban JW, Claridge DE, Balcomb KC, Noble LS, Thompson PM: **Kinship as a basis for alliance formation between male bottlenose dolphins, *Tursiops truncatus*, in the Bahamas**. *Animal Behaviour* 2003, **66:** 185–194.

194. Möller LM, Beheregaray LB, Harcourt RG, Krutzen M: **Alliance membership and kinship in wild male bottlenose dolphins (*Tursiops aduncus*) of southeastern Australia.** *Proceedings of the Royal Society of London, Series B* 2001, **268:** 1941–1947.

195. Lusseau D, Schneider K, Boisseau OJ, Haase P, Slooten E, Dawson SM: **The bottlenose dolphin community of Doubtful Sound features a large proportion of long-lasting associations. Can geographic isolation explain this unique trait?** *Behavioral Ecology Sociobiology* 2003, **54:** 396–405.

196. Connor RC, Whitehead H: **Alliances II. Rates of encounter during resource utilization: a general model of intrasexual alliance formation in fission-fusion societies.** *Animal Behaviour* 2005, **69:** 127-132.

197. Gero S, Bejder H, Whitehead H, Mann J, Connor RC: **Behaviourally specific preferred associations in bottlenose dolphins *Tursiops* spp**. *Canadian Journal of Zoology* 2005, **83**:1566-1573.

198. Rogers C: **The social structure of bottlenose dolphins, *Tursiops truncatus* in the Bahamas**. *Marine Mammal Science* 2006, **20**:688-708.

199. Krützen M, Barré LM, Connor RC, Mann J, Sherwin WB: **‘O father:where art thou?’- Paternity assessment in an open fission-fusion society of wild bottlenose dolphins (*Tursiops* sp.) in Shark Bay, Western Australia**. *Molecular Ecology* 2004, **13**:1975-1990.

200. Amano M, Miyazaki N, Yanagisawa F: **Life history of Fraser’s dolphin, *Lagenodelphis hosei*, based on a school captured off the Pacific coast of Japan**. *Marine Mammal Science* 1996,**12**:199-214.

201. Dolar MLL: **Fraser’s Dolphin (*Lagenodelphis hosei*).** In: *Encyclopedia of Marine Mammals* Edited by Perrin WF, Wursig B, Thewissen JGM. San Diego: Academic Press; 2002:485-486.

202. Jefferson TA, Leatherwood S: ***Lagenodelphis hosei***. *Mammalian Species* 1994, **470:** 1-5.

203. Leatherwood S, Jefferson TA, Norris JC, Stevens WE, Hansen LJ, Mullin KD: **Occurrence and sounds of Fraser’s dolphins (*Lagenodelphis hosei*) in the gulf of Mexico**. *The Texas Journal of Science* 1993, **45:** 349-354.

204. Perrin WF, Leatherwood S, Collet A: **Fraser’s dolphin-*Lagenodelphis hosei* (Fraser 1956).** In: *Handbook of Marine Mammals* Edited by Ridgway SH, Harrison Sir H. New York: Academic Press; 1994: 225-241. [vol.5].

205. Saayman GS, Tayler CK: **The socioecology of humpback dolphins (*Sousa* sp.).** In: *The behaviour of marine animals* Edited by Winn HE, Olla BL. New York: Plenum Press; 1979:165-226. [vol.3].

206. Karczmarski L: **Group dynamics of humpback dolphins (*Sousa chinensis*) in the Algoa Bay region, South Africa**. *Journal of Zoology London* 1999, **249:** 283–293.

207. Karczmarski L, Cockcroft VG, McLachlan A:**Group size and seasonal pattern of occurrence of humpback dolphins *Sousa chinensis* in Algoa Bay, South Africa**. *South African Journal of Marine Science* 1999, **21:** 89–97.

208. Karczmarski L, Winter P, Cockcroft VG, McLachlan A: **Population analyses of Indo-Pacific humpback dolphins *Sousa chinensis* in Algoa Bay, Eastern Cape, South Africa**. *Marine Mammal Science* 1999, **15:** 1115–1123.

209. Guissamulo AT, Cockcroft VG: **Ecology and population estimates of the Indo-Pacific humpback dolphins (*Sousa chinensis*) in Maputo Bay, Mozambique.** *Aquatic Mammals* 2004, **30:** 94–102.

210. Jefferson TA, Karczmarski L: ***Sousa chinensis***. *Mammalian Species* 2001, **655**:1-9.

211. Da Silva VMF, Best RC: **Tucuxi-*Sotalia fluviatilis* (Gervais 1853).** In: *Handbook of Marine Mammals* Edited by Ridgway SH, Harrison Sir H. New York: Academic Press; 1994: 43-70. [vol.5].

212. Da Silva VMF, Best RC: ***Sotalia fluviatilis*.** *Mammalian Species* 1996, **527:** 1–7.

213. Montero-Filho ELA: **Group organization of the dolphin *Sotalia fluviatilis guianensis* in an estuary of southeastern Brazil***. Ciencia e Cultura Journal of the Brazilian Association for the Advancement of Science* 2000, **52:** 97-101.

214. Flores PAC: **Tucuxi *Sotalia fluviatilis*.** In: *Encyclopedia of Marine Mammals* Edited by Perrin WF, Wursig B, Thewissen JGM. San Diego: Academic Press; 2002: 1267–1269.

215. Azevedo AF, Viana SC, Oliveira AM, Van Sluys M: **Group characteristics of marine tucuxis (*Sotalia fluviatilis*) (Cetacea: Delphinidae) in Guanabara Bay, south-eastern Brazil**. *Journal of the Marine Biology Association of the United Kingdom* 2005, **85**:209-212.

216. Gamboa-Poveda M, May-Collado LJ: **Insights on the occurrence, residency, and behavior of two coastal dolphins from Gandoca-Manzanillo, Costa Rica: *Sotalia guianensis* and *Tursiops truncatus* (Family Delphinidae).** *International Whaling Commission Scientific Committee Meeting Document* 2006, **SC/58/SM4**: 1-9.

217. Acevedo-Gutiérrez A, DiBerardinis A, Larkin S., Larkin K., Forestell P: **Social interactions between tucuxis and bottlenose dolphins in Gandoca-Manzanillo, Costa Rica**. *Latin American Journal of Aquatic Mammals* 2005, **4**:49-54.

218. Geise L, Gomes N, Cerqueira R: **Behavior, habitat use and population size of *Sotalia fluviatilis* (Gervais, 1853) (Cetacea, Delphinidae) in the Cananeia Estuary Region, São Paulo, Brazil**. *Revista Brasileira de Biologia* 1999, **59**:183-194.

219. Mobley JR, Spitz SS, Forney KA, Grotefendt R, Forestell PH: **Distribution and abundance of odontocete species in Hawaiian waters: preliminary results of 1993-98 aerial surveys**. *Southwest Fisheries Science Center Administrative Report* 2000, LJ-00-14C.

220. Miyasaki N, Perrin WF: **Rough-toothed dolphin- *Steno bredanensis* (Lesson 1828).** In: *Handbook of Marine Mammals* Edited by Ridgway SH, Harrison Sir H. New York: Academic Press; 1994: 1-22. [vol.5].

221. Jefferson TA: **Rough-Toothed Dolphin (*Steno bredanensis*).** In: *Encyclopedia of Marine Mammals* Edited by Perrin WF, Wursig B, Thewissen JGM. San Diego: Academic Press; 2002: 1055-1059.

222. Perkins JS, Miller W: **Mass stranding of Steno bredanensis in Belize**. *Biotropica* 1983, **15**:235-236.

223.Ferrero RC, Hodder J, Cesarone J: **Recent strandings of rough-toothed dolphins, *Steno bredanensis*, on the Oregon and Washington coasts**. *Marine Mammal Science* 1994, **10**:114-6.

224. Ross GJB, Leatherwood S: **Pygmy killer whale *Feresa attenuata* Gray 1874.** In: *Handbook of Marine Mammals* Edited by Ridgway SH, Harrison Sir H. New York: Academic Press; 1994: 487-404. [vol.5].

225. Donahue MA, Perryman WL: **Pygmy Killer Whale (*Feresa attenuata*).** In: *Encyclopedia of Marine Mammals* Edited by Perrin WF, Wursig B, Thewissen JGM. San Diego: Academic Press; 2002:1009-1010.

226. Mignucci-Giannoni AA, Toyos-González GM, Pérez-Padilla J, Rodríguez-López MA, Overing J: **Mass stranding of pygmy killer whales (*Feresa attenuata*) in the British Virgin Islands.** *Journal of the Marine Biology Association of the United Kingdom* 2000, **80**:759-760.

227. Williams AD, Williams R, Brereton T: **The sighting of pygmy killer whales (*Feresa attenuata)* in the southern Bay of Biscay and their association with cetacean calves.** *Journal of the Marine Biology Association of the United Kingdom* 2002, **82**:509-511.

228. Amos B, Schlötterer C, Tautz D: **Social structure of pilot whales revealed by analytical DNA profiling**. *Science* 1993, **260**: 670–672.

229. Bernard HJ, Reilly SB: ***Globicephala* (Lesson 1828).** In: *Handbook of Marine Mammals* Edited by Ridgway SH, Harrison Sir H. New York: Academic Press; 1999: 245-280. [vol.6.].

230. Norris KS. Prescott JH: **Observations on Pacific cetaceans of California and Mexican waters.** *University of California Publications in Zoology* 1961, **63:** 291-402.

231. Kasuya, T. **Japanese fisheries exploiting southern form short-finned pilot whales*.*** *International Whaling Commission Scientific Committee Meeting Document* 1986. **SC/38/SM19**: 1-12.

232. Heimlich-Boran JR: **Social organisation of the short-finned pilot whale, *Globicephala macrorhynchus*, with special reference to the comparative social ecology of Delphinids.** PhD Thesis. Cambridge University; 1993.

233. Whitehead H, Rendell L, Osborne RW, Würsig B: **Culture and conservation of non-humans with reference to whales and dolphins: review and new directions**. *Biological Conservation* 2004, **120**:427-437.

234 Connor RC: **Group living in whales and dolphins**. In: *Cetacean Societies: Field studies of dolphins and whales.* Edited by Mann J, Connor RC, Tyack PL, Whitehead H. Chicago: The University of Chicago Press; 2000: 199-218.

235. Ottensmeyer A, Whitehead H: **Behavioural evidence for social units in long finned pilot whales**. *Canadian Journal of Zoology* 2003, **81:** 1327–1338.

236. Weilgart, LS, Whitehead H: **Vocalizations of the North Atlantic pilot whale (*Globicephala melas*) as related to behavioural contexts**. *Behavioral Ecology and Sociobiology* 1990, **26:** 399–402.

237. Fullard KJ, Early G, Heide-Jorgensen MP, Bloch D, Rosing-Asvid A, Amos W: **Population structure of long-finned pilot whales in the North Atlantic: a correlation with sea surface temperature?** *Molecular Ecology* 2002, **9:** 949–958.

238. Andersen LW, Siegismund HR: **Genetic evidence for migration of males between schools of the long-finned pilot whale *Globicephala melas***. *Marine Ecology Progress* series 1994, **105**:1-7.

239. Amano M, Miyazaki N: **Composition of a school of Risso’s dolphins, *Grampus griseus*.** *Marine Mammal Science* 2004, **20**:152-160.

240. Zucca P, Di Guardo G, Francese M, Scaravelli D, Genov T, Mazzatenta A: **Causes of stranding in four Risso’s dolphins (*Grampus griseus*) found beached along the North Adriatic Sea coast.** *Veterinary Research Communications* 2005, **29**: 261-264.

241. Baird RW: (2002a). **Risso’s Dolphin (*Grampus griseus*).** In: *Encyclopedia of Marine Mammals* Edited by Perrin WF, Wursig B, Thewissen JGM. San Diego: Academic Press; 2002:1037–1039.

242. Kruse S: **Aspects of the biology, ecology, and behavior of Risso’s dolphis (*Grampus griseus*) off the California coast**. *M.Sc. Thesis*. University of California at Santa Cruz; 1989.

243. Leatherwood S, Hubbs CL, Fisher M: **First records of Risso’s dolphin (*Grampus griseus*) from the Gulf of California with detailed notes on mass stranding**. *Transactions of the San Diego Society of Natural History* 1979, **19:** 45-52.

244. Jefferson TA, Barros NE: ***Peponocephala electra***. *Mammalian Species* 1997, **553:** 1-6.

245. Miyasaki N, Yoshihiro F, Iwata K: **Biological analysis of a mass stranding of melon-headed whales (*Peponochela electra*) at Aoshima, Japan**. *Bulletin of the National Science Museum Series A Tokyo* 1998, **24:** 31-60.

246. Perryman WL: **Melon-headed whale (*Peponocephala electra*).** In: *Encyclopedia of Marine Mammals* Edited by Perrin WF, Wursig B, Thewissen JGM. San Diego: Academic Press; 2002:733-734.

247. Stayce PJ, Leatherwood S, Baird RW: ***Pseudorca crassidens*.** *Mammalian Species* 1994, **456:** 1-6.

248. Odell DK, McClune KM: **False Killer Whale-*Pseudorca crassidens****.* In: *Handbook of Marine Mammals* Edited by Ridgway SH, Harrison Sir H. New York: Academic Press; 1999: 213-244. [vol.6].

249. Baird RW: False Killer Whale (*Pseudorca crassidens*). In: *Encyclopedia of Marine Mammals* Edited by Perrin WF, Wursig B, Thewissen JGM. San Diego: Academic Press; 2002:411–412.

250. Amano M: **Odontoceti social structure and life history parameters: interspecific comparisons**. *Kyoto Conference, Delphinid and Primate Social Ecology: a comparative discussion*. Kyoto, Japan, July 2005: 29-30.

251. Arnold PW: **Irrawaddy dolphin (*Orcaella brevirostris*).** In: *Encyclopedia of Marine Mammals* Edited by Perrin WF, Wursig B, Thewissen JGM. San Diego: Academic Press; 2002: 652-655.

252. Marsh H, Lloze R, Heinsohn GE, Kasuya T. **Irrawady Dolphin, *Orcaella brevirostris* (Gray 1866)**. In: *Handbook of Marine Mammals* Edited by Ridgway SH, Harrison Sir H. New York: Academic Press; 1989:101-118. [vol.4].

253. Parra GJ, Corkeron PJ, Marsh H: **Population sizes, site fidelity and residence patterns of Australian snubfin and Indo-Pacific humpback dolphins: Implications for conservation.** *Biological Conservation* 2006**, 129:** 167-180

254. Stacey PJ, Arnold PW: ***Orcaella brevirostris***. *Mammalian Species* 1999, **616**:1-8.

255. Bigg MA, Olesiuk PF, Ellis GM, Ford JKB, Balcomb KC: **Social organization and genealogy of resident killer whales (*Orcinus orca*) in the coastal waters of British Columbia and Washington State**. *Report of the International Whaling Commission, Special Issue* 1990*,* **12:** 383-405.

256. Baird RW, Abrams PL, Dill LM: **Possible indirect interactions between transient and resident killer whales: implications for the evolution of foraging specializations in the genus *Orcinus*.** *Oecologia* 1992, **89:** 125-132.

257. Baird RW, Dill LM: **Ecological and social determinants of groups size in transient killer whales**. *Behavioral Ecology* 1996, **7:** 408-16.

258. Baird RW, Whitehead H: **Social organization of mammal-eating killer whales: group stability and dispersal patterns**. *Canadian Journal of Zoology* 2000, **78:** 2096–2105.

259. Baird RW: **The killer whale: foraging specializations and group hunting**. In: *Cetacean Societies: Field Studies of Dolphins and Whales* Edited by Mann J, Connor RC, Tyack PL, Whitehead H. Chicago: University of Chicago Press; 2000:127-154.

260. Yurk H, Barrett-Lennard LG, Ford JKB, Matkin CO: **Cultural transmission within maternal lineages: Vocal clans in resident killer whales in Southern Alaska**. *Animal Behaviour* 2002, **63:** 1103-1119.

261. Ford JKB: **Vocal traditions among resident killer whales (*Orcinus orca*) in coastal waters of British Columbia**. *Canadian Journal of Zoology* 1991, **69:** 1454-1483.

262. Heyning JE, Dahlheim ME: ***Orcinus orca***. *Mammalian Species* 1988, **304**:1-9.

263. Brault S, Caswell H: **Pod-specific demography of killer whales (*Orcinus orca*).** *Ecology* 1993, **74**:1444-1454.

264. Hoezel AR, Dahlheim M, Stern SJ: **Low genetic variation among whales (Orcinus orca) in the Eastern North Pacific and genetic differentiation between foraging specialists.** *Journal of Heredity* 1998, **89**:121-128.

265. Hoezel AR, Natoli A, Dahlheim ME, Olavarria C, Baird RW, Black NA: **Low worldwide genetic diversity in the killer whale (*Orcinus orca*): implications for demographic history.** *Proceeding of the Royal Society of London, Series B* 2002, **269**:1467-1473.

266. Guinet C: **Intentional stranding apprenticeship and social play in killer whales (*Orcinus orca*).** *Canadian Journal of Zoology* 1991, **69**:2712-2716.
