# Supplementary figures and images for: Phylogenetic review of tonal sound production in whales in relation to sociality
Source: BMC Evol Biol. 2007 Aug 10;7:136. doi: 10.1186/1471-2148-7-136 (PMC2000896; doi:10.1186/1471-2148-7-136)

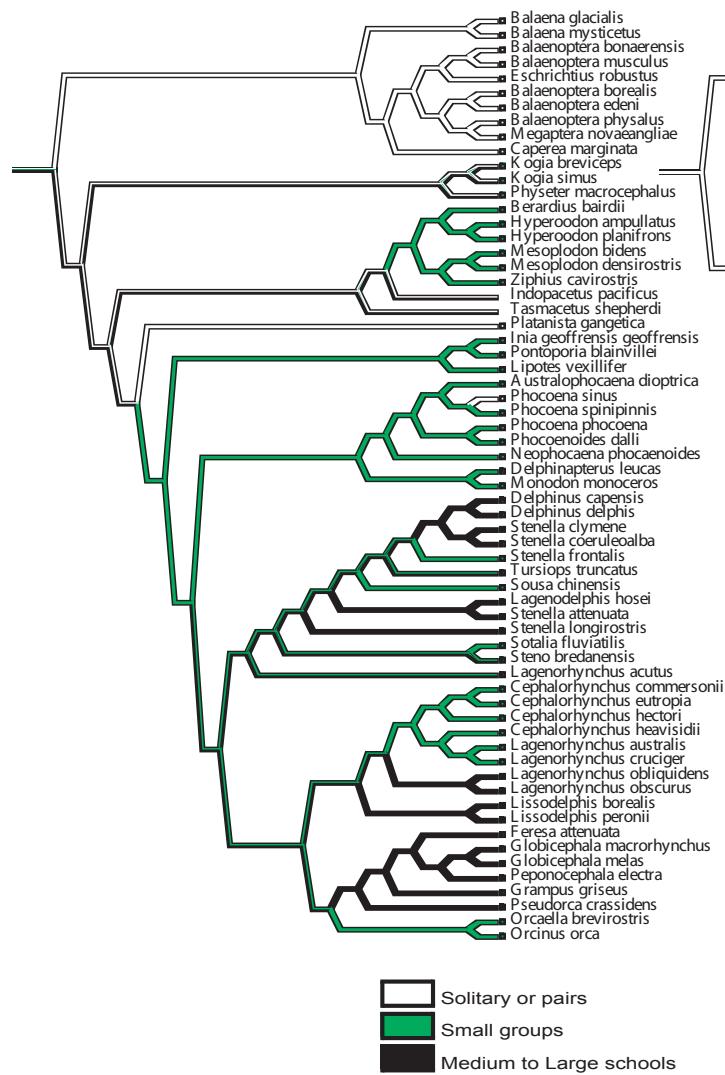

a. Group Size

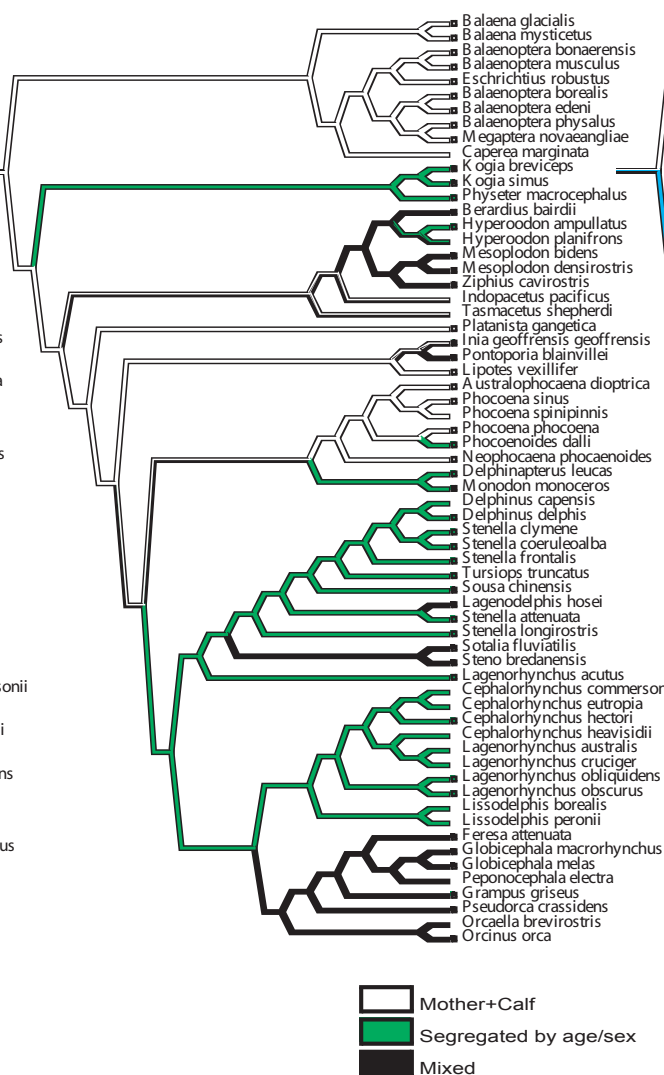

b. Group Composition

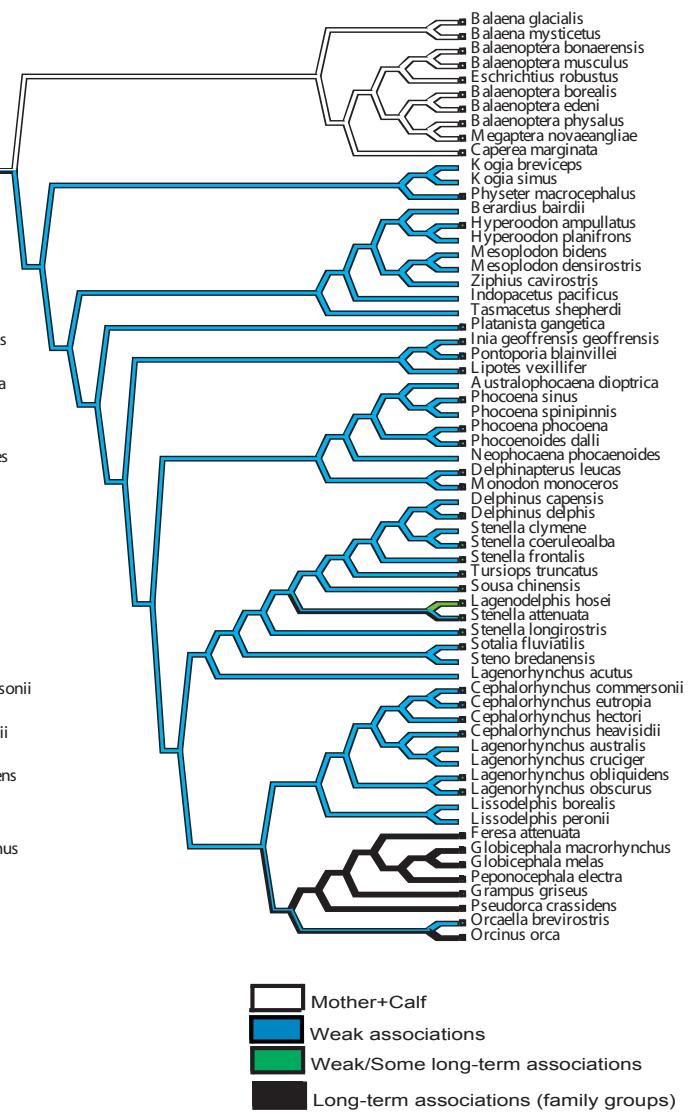

c. Group Stability/Associations

Supplement: Additional file 6 — Optimization of components of sociality. This figure shows social components optimization (a = group size, b = group composition, c = group stability/association patterns) on the preferred phylogeny. Note that this optimization contains polymorphic species and thus family based group like Physeter and Monodon and species with long-term associations between non-related group members are all optimized using the lowest state of sociality. [file 1471-2148-7-136-S6.pdf]

p=0.03 (6Gains,2Losses)

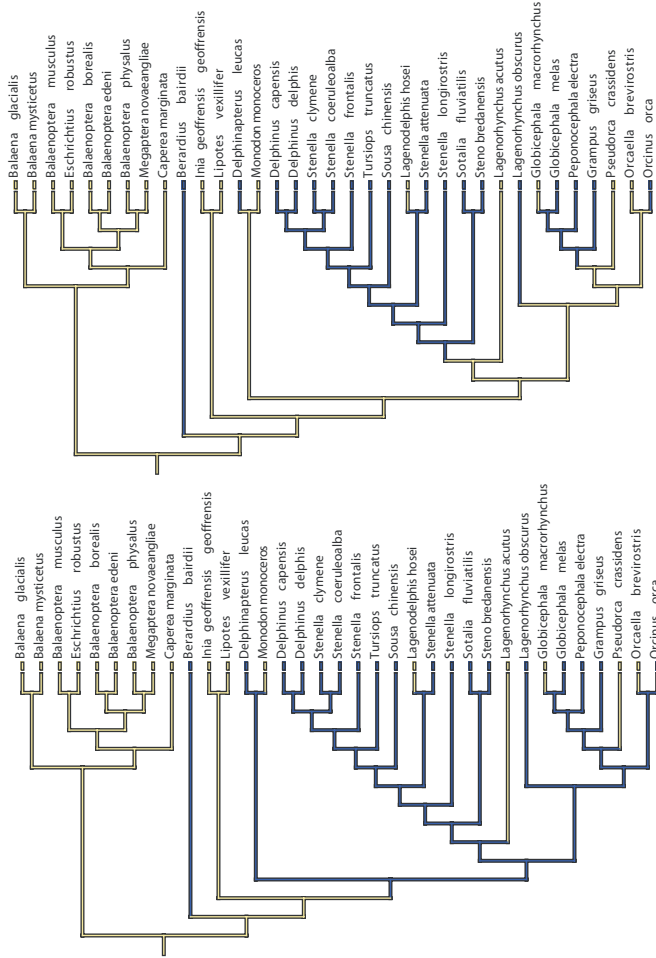

p=0.03 (2Gains,6Losses)

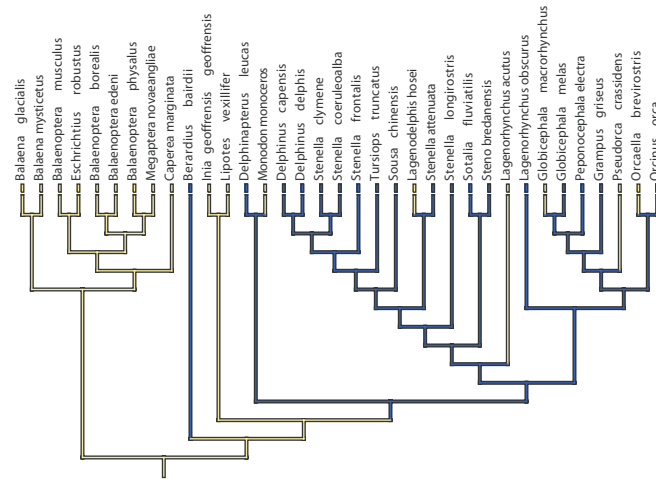

p=0.04 (4Gains,4Losses)

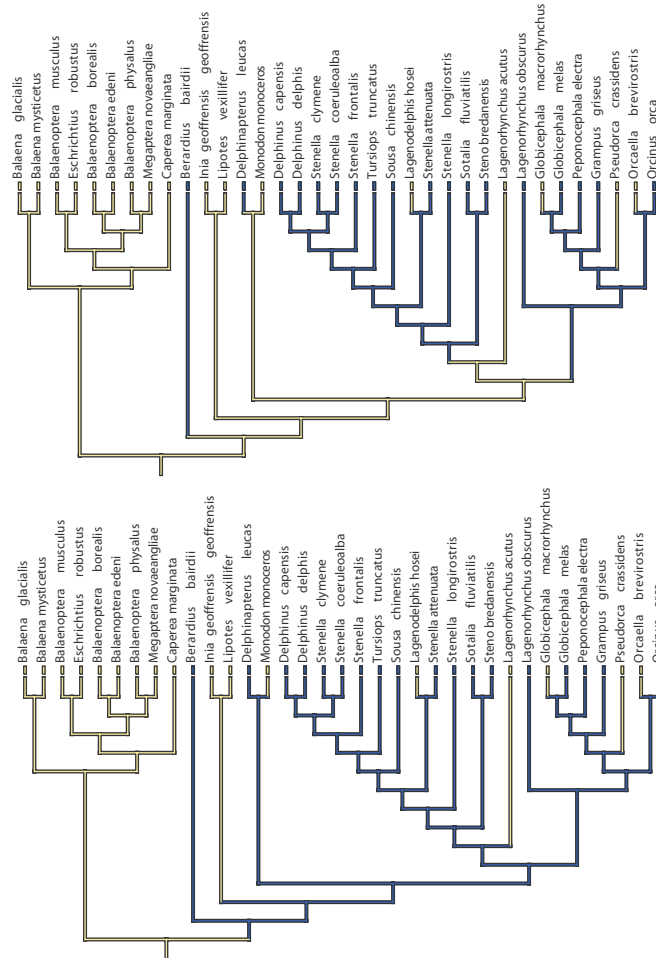

p=0.14(1Gain,7Losses)

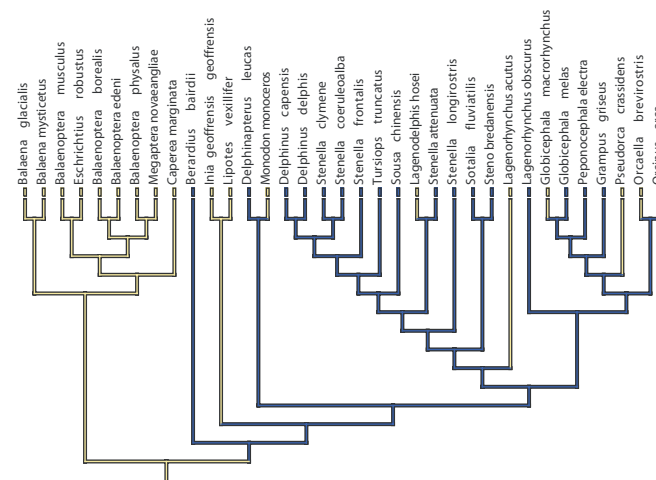

p=0.05(3Gains,5Losses)

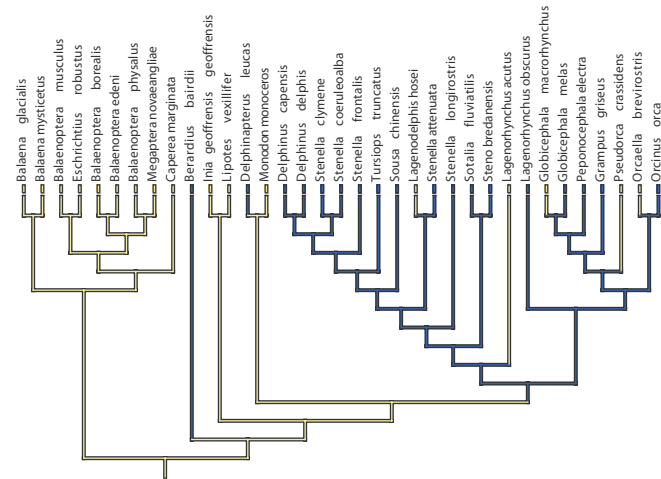

Supplement: Additional file 8 — Optimization of tonal sound complexity and the association between sociality and tonal sound complexity. Most parsimonious optimizations of tonal sound complexity (based on mean number of inflection points, MIP) and results from the concentrated changes test for sociality and tonal sound complexity (yellow = state 0, tonal sounds with MIP ≤ 1, blue = state 1, tonal sounds with MIP > 1). [file 1471-2148-7-136-S8.pdf]

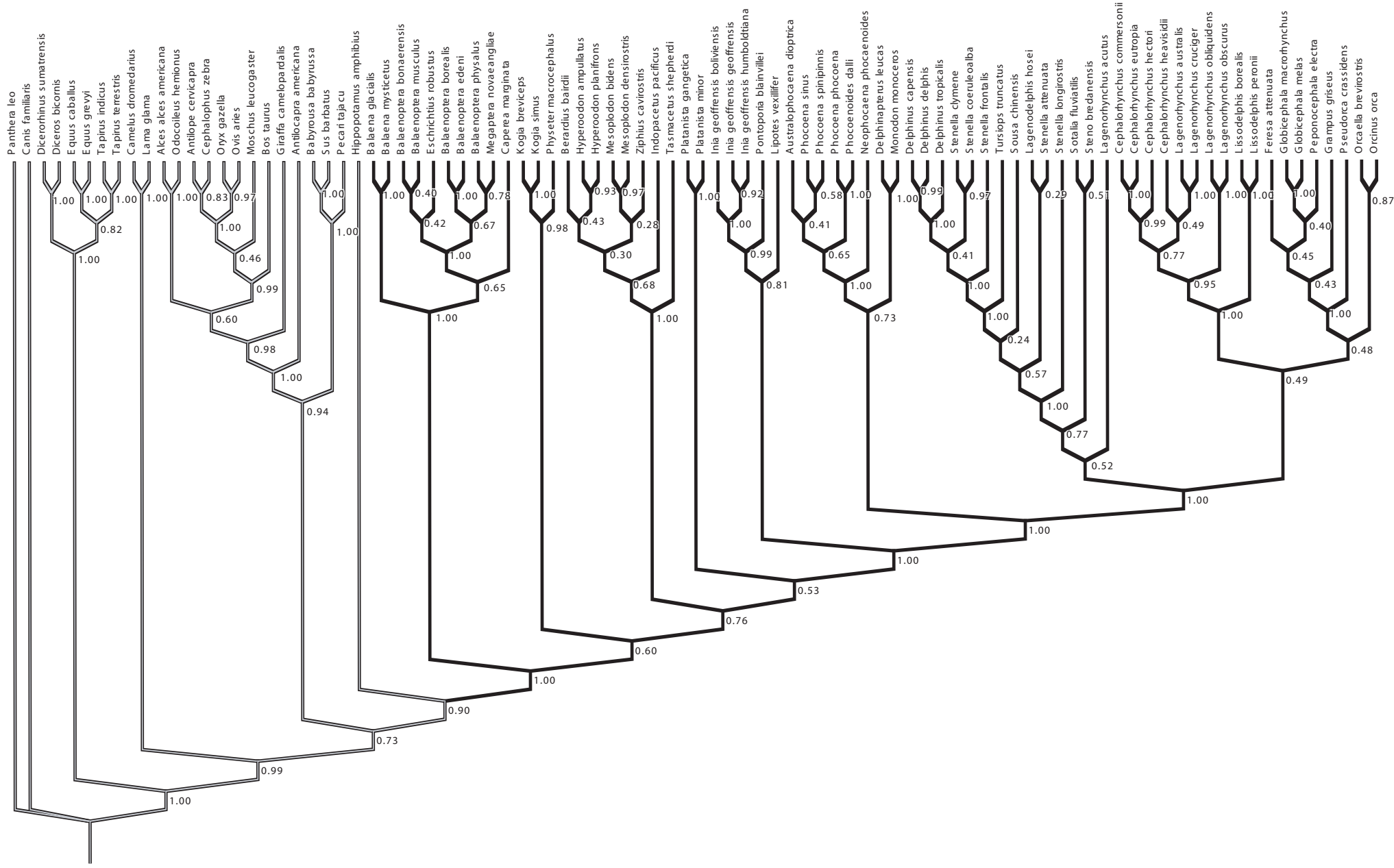

Supplement: Additional file 13 — Phylogeny of Cetacea. This figure reproduces the preferred phylogenetic hypothesis of May-Collado et al. (2007), used here for all main analyses. Numbers on nodes represent posterior probabilities. [file 1471-2148-7-136-S13.pdf]
