# Supplementary material for: Phylogenetic review of tonal sound production in whales in relation to sociality
Source: BMC Evol Biol. 2007 Aug 10;7:136. doi: 10.1186/1471-2148-7-136 (PMC2000896; doi:10.1186/1471-2148-7-136)
Supplement: Additional file 7 — Cetacean tonal sound acoustic parameters. This table reviews published data on cetacean tonal sound acoustic parameters. Numbers in bold correspond to the preferred value used in the optimizations (see Methods). [file 1471-2148-7-136-S7.doc]

| Species | Max  (kHz) | MMax  (kHz) | Min  (kHz) | MMin  (kHz) | MaxD  (s) | MD  (s) | MinD  (s) | Complexity [Inflection points-IP] | | | References |
| --- | --- | --- | --- | --- | --- | --- | --- | --- | --- | --- | --- |
|  |  |  |  |  |  |  |  | Mean | IP-two state | IP-four state |  |
| **Baleen Whales**  **(Mysticeti)**  Balaenidae |  |  |  |  |  |  |  |  |  |  |  |
| *Eubalaena glacialis* | 11.23 | 3.14 | 0.02 | 0.05 | 2.08 | 0.99 | 0.26 | 1≤* | 0 | 0 | 267 |
| *B. mysticetus* | **2**  1 | 0.165  **0.3** | 0.09  **0.02** | **0.02**  0.05 |  | 1.1 |  | 1≤* | 0 | 0 | 268  269 |
| Balaenopteridae |  |  |  |  |  |  |  |  |  |  |  |
| *Balaenoptera bonaerensis* | ? | ? | ? | ? | ? | ? | ? | ? | ? | ? |  |
| *B. borealis* | **3.5**  1 | ? | 1.5  ~**0.1** | ? |  | 0.04  **1.1** |  | 1≤* | 0 | 0 | 270  271 |
| *B. edeni/ B. brydei* | **0.245**  0.180  0.079 |  | 0.07  0.09  **0.0207** |  |  | 0.42  **2.3** |  | 1≤* | 0 | 0 | 272  273  274 |
| *B. musculus* | 0.0185  0.0202  0.122  0.052  0.0277  **0.242** | 0.0189  **0.0883** | 0.0157  0.0182  **0.011**  0.050  0.0189  0.0143 | 0.0172  **0.0166** | 10  **28.2** | 11  10  **29**  0.80  8  16  22.8 | 3  **1** | 1≤* | 0 | 0 | 275  276  277  278  279  280  281  282 |
| *B. physalus* | **0.118**  0.058 | 0.023  **0.042** | **0.010**  0.017 | **0.018**  0.020 |  | **1**  0.7 |  | 1≤* | 0 | 0 | 283  284  285 |
| *Megaptera novaeangliae* | 4 | 1.315 | 0.25 | 0.925 |  | 0.96 |  | 1≤* | 0 | 0 | 286 |
| Eschrichtidae |  |  |  |  |  |  |  |  |  |  |  |
| *Eschrichtius robustus* | **0.2**  0.2 | 0.3 | **0.02**  0.1 | 0.25 |  | 1.54 |  | 1≤* | 0 | 0 | 287  288  289 |
| Neobalaenidae |  |  |  |  |  |  |  |  |  |  |  |
| *Capera marginata* | 0.135 | ? | 0.06 | ? |  | 0.18 |  | 1≤* | 0 | 0 | 290 |
| **Toothed Whales**  **(Odontoceti)**  Kogiidae |  |  |  |  |  |  |  |  |  |  |  |
| *Kogia breviceps* | ? | ? | ? | ? | ? | ? | ? | ? | ? | ? |  |
| *K. simus* | ? | ? | ? | ? | ? | ? | ? | ? | ? | ? |  |
| Physeteridae |  |  |  |  |  |  |  |  |  |  |  |
| *Physeter macrocephalus* | n/a | n/a | n/a | n/a | n/a | n/a | n/a | n/a | n/a | n/a |  |
| Ziphiidae |  |  |  |  |  |  |  |  |  |  |  |
| *Berardius bairdii (B. anurxii)* | 8.0 | ? | 4 | ? | ~3.5 |  | ~2 | ~3 | 1 | 2 | 46 |
| *Hyperoodon ampullatus* | n/a | n/a | n/a | n/a | n/a | n/a | n/a | n/a | n/a | n/a |  |
| *H. planifrons* | ? | ? | ? | ? | ? | ? | ? | ? | ? | ? |  |
| *Mesoplodon bidens* | ? | ? | ? | ? | ? | ? | ? | ? | ? | ? |  |
| *M. densirostris* | ? | ? | ? | ? | ? | ? | ? | ? | ? | ? |  |
| *Ziphius cavirostris* | ? | ? | ? | ? | ? | ? | ? | ? | ? | ? |  |
| Platanistidae |  |  |  |  |  |  |  |  |  |  |  |
| *Platanista gangetica* | ? | ? | ? | ? | ? | ? | ? | ? | ? | ? |  |
| Iniidae |  |  |  |  |  |  |  |  |  |  |  |
| *Inia geoffrensis* | **48.10**  5.16  13 | **24.71**  2.97 | 5.03  **0.22**  3 | 15.06  **2.54** | 0.080  **4.42** | 0.009  **1.14** | **0.002**  0.16 | 1.05 | 1 | 1 | 291  292-293  294 |
| Pontoporidae |  |  |  |  |  |  |  |  |  |  |  |
| *Pontoporia blainvillei* | ? | ? | ? | ? | ? | ? | ? | ? |  |  |  |
| Lipotidae |  |  |  |  |  |  |  |  |  |  |  |
| *Lipotes vexillifer* | 4.5  **4.6** | 5.84  **6.1** | 3.8  3.8 | 5.0  **4.9** | **1.8**  1.6 | 1  1 | **0.4**  0.5 | 0.72 | 1 | 0 | 295  296 |
| Phocoenidae |  |  |  |  |  |  |  |  |  |  |  |
| *Phocoena dioptrica* | n/a | n/a | n/a | n/a | n/a | n/a | n/a | n/a | n/a | n/a |  |
| *Phocoena phocoena* | n/a | n/a | n/a | n/a | n/a | n/a | n/a | n/a | n/a | n/a |  |
| *P. sinus* | n/a | n/a | n/a | n/a | n/a | n/a | n/a | n/a | n/a | n/a |  |
| *P. spinipinnis* | n/a | n/a | n/a | n/a | n/a | n/a | n/a | n/a | n/a | n/a |  |
| *Phocoenoides dalli* | n/a | n/a | n/a | n/a | n/a | n/a | n/a | n/a | n/a | n/a |  |
| *Neophocaena phocaenoides* | n/a | n/a | n/a | n/a | n/a | n/a | n/a | n/a | n/a | n/a |  |
| Monodontidae |  |  |  |  |  |  |  |  |  |  |  |
| *Monodon monocerus* | **18**  10  8.84 | 7.18 | **0.3**  5  0.360 | 0.718 | **6**  0.85  1.26 | 1.19 | **0.05**  0.1  0.68 | 1≤*  1≤*  1≤* | 0 | ? | 297  298  299 |
| *Delphinapterus leucas* | **19.6**  15.8 | 6.8  4.33  **11.65** | **0.2**  0.4 | 3.8  3.38  **1.99** | **3.92**  3.2 | 1.06  0.75  **1.12** | **0.01**  0.05 | 1>*  1>*  **13.5** | 1 | 3 | 300  301  302 |
| Delphinidae |  |  |  |  |  |  |  |  |  |  |  |
| *Cephalorhynchus commersonii* | n/a | n/a | n/a | n/a | n/a | n/a | n/a | n/a | n/a | n/a |  |
| *C. eutropia* | n/a | n/a | n/a | n/a | n/a | n/a | n/a | n/a | n/a | n/a |  |
| *C. hectori* | n/a | n/a | n/a | n/a | n/a | n/a | n/a | n/a | n/a | n/a |  |
| *C. heavisidii* | n/a | n/a | n/a | n/a | n/a | n/a | n/a | n/a | n/a | n/a |  |
| *Lagenorhynchus australis* | n/a | n/a | n/a | n/a | n/a | n/a | n/a | n/a | n/a | n/a |  |
| *L.cruciger* | ? | ? | ? | ? | ? | ? | ? | ? | ? | ? |  |
| *L. obliquidens* | ~13 | ~10 | ~1 | ? | 0.2 | 0.7 | 1.2 | ~>1 | 1 | 0 | 303 |
| *L. obscurus* | 27.3 | **16.49**  13.22 | 1.04 | **8.11**  8.15 | **3.14**  1.04 | **1.03**  0.535 | 0.18  **0.014** | 1.97 | 1 | 1 | 292  304 |
| *L. acutus* | ? | 12.14 | ? | 8.21 |  | 0.5 |  | 0.92 | 1 | 0 | 305 |
| *Lissodelphins borealis* | ? | ? | ? | ? | ? | ? | ? | ? | ? | ? |  |
| *L. peronii* | ? | ? | ? | ? | ? | ? | ? | ? | ? | ? |  |
| *Delphinus delphis* | 19.8 | 11.65  **13.6** | 4.8 | **6.42**  7.4 |  | 0.8 |  | 1>*  **1.2** | 1 | 1 | 306  307 |
| *Delphinus capensis* | ? | 15.5 | ? | 7.7 |  | 0.70 |  | 1.3 | 1 | 1 | 307 |
| *Stenella attenuata* | 21.4 | 15.72  **18.7** | 3.13 | 8.73  **8.2** | 1.95 | 0.53  **0.9** | 0.09 | 0.70  **1.9** | 1 | 1 | 292  307 |
| *S. clymene* | ? | **19.2**  13.62 | ? | **6.33**  9.25 |  | **0.61**  0.41 |  | 1>* | 1 | ? | 308  309 |
| *S. frontalis* | 19.8 | 16.04 | 5 | 7.91 | 2.07 | 0.82 | 0.08 | 3.43 | 1 | 3 | 292 |
| *S. coeruleoalba* | 22.99 | 11.53  **14.8** | 1.1 | **6.84**  8.1 |  | 0.54  **0.8** |  | 1.3  **1.9** | 1 | 1 | 310  307 |
| *S. longirostris* | 22.5  24  **25.25**  23.04 | 15.2  13.7  16.5  **17.56**  16.8  14.32 | 3.91  **0.85**  4 | 9.03  9.1  9.99  9.66  10.19  **8.76** | 1.82  3.35  **4.49**  1.87 | **0.75**  0.6  0.72  0.49  0.61  0.43 | 0.10  0.016  0.013  **0.040** | 1.07  **1.9**  0.55 | 1 | 1 | 292  307  311  312  313  305 |
| *Tursiops truncatus* | **41**  21.6 | **17.2**  11.35  11.95  16.24 | 1.86  **0.94** | 7.4  **5.46**  7.33 | 3.20  3.20 | 0.86  **1.4**  0.70  0.75  1.3 | 0.05  0.05 | **3.7**  1.86  2.14  2.86 | 1 | 3 | 314  307  292  315  305 |
| *Lagenodelphis hosei* | 24.0  18.3  13.4 | **16.9**  14.9 | 4.3  6.6 | 9.36  11  **7.64** | 0.5 | **0.77**  0.46 | 0.4 | 0.80 | 1 | 0 | 316  317  318 |
| *Sousa chinensis* | **22**  20 | 16.3 | **0.9**  3  1.2 | 4.5 | 1.3 | **1.1**  0.2  0.13 | 0.01 | 1>* | 1 | ? | 319  320  321 |
| *Sotalia fluviatilis*** | 23.9  **38.25**  18  17.49 | 19.95  15.41  **21.32**  13  13.312  15.65 | 1.34  3.65  2.714  **0.5**  1.031 | **7.21**  10.2  13.14  7.6  10.521  9.18 | **1.064**  1.04  0.852  2.2 | 0.381  0.41  0.103  **0.79**  0.63 | 0.038  0.06  0.01  **0.009** | 0.77  **1.38**  0.7  1.3 | 1 | 1 | 322  292, 293  Authors unpublished data  323  324  325 |
| *Steno bredanensis* | 7.0 | 9.1 | 4 | 6.03 |  | 0.6 |  | 1.3 | 1 | 1 | 326  307 |
| *Feresa attenuata* | ? | ? | ? | ? | ? | ? | ? | ? | ? | ? |  |
| *Globicephala macrorhynchus* | 23.6 | **10.87**  6.1 | 0.24 | 6.25  **3.6** |  | **0.56**  0.4 |  | 0.69  **0.7** | 1 | 0 | 327  307 |
| *G. melas* | 21.2 | **8.86**  4.716 | 0.32 | **3.48**  2.82 |  | **0.72**  0.71 |  | 0.98  **1.01** | 1 | 1 | 327  305 |
| *Grampus griseus* | 23.8 | **20**  13.44 | 1.90 | **3.9**  8.83 |  | 4.9  **0.53** |  | 1>*  **1.37** | 1 | 1 | 328  327 |
| *Peponocephala electra* | 24.5 | 12.14 | 5.5 | 8.381 | 0.9 | 0.54 | 0.1 | **1.05**  0.04 | 1 | 1 | 317  329 |
| *Pseudorca crassidens* | 18.1 | **8.29**  6.1 | 1.87 | 5.43  **4.7** |  | **0.56**  0.4 |  | **0.75**  0.5 | 1 | 0 | 327  307 |
| *Orcaella brevirostris*** | 6.0 | 4.2 | 1.1 | 3.2 | 0.3 | 0.3 | 0.1 | ~1≤ | 1 | 0 | 330 |
| *Orcinus orca* | **18**  8.9  16.7 | 6.61  9.9  **12.64** | 0.05  2.4 | **1.5**  4.27  5.4  3.36 | 18.3 | **1.8**  1.11 | 0.06 | 1>  **21.14** | 1 | 3 | 331  332  333  334  335 |

**In this paper these species are still treated as one single species (with two ecotypes: riverine and marine), however there is recent evidence that each may be a separate species (see details in references 115 and 116).

**References**

267. Parks SE, Tyack PL: **Sound production by North Atlantic right whales (*Eubalaena glacialis*) in surface active groups**. *Journal of the Acoustical Society of America* 2005,**117**: 3297-3306.

268. Ljungblad DK, Thompson PO, Moore SE: **Underwater sounds recorded from migrating bowhead whales, *Baleana mysticetus*, in 1979**. *Journal of the Acoustical Society of America* 1982, **71**:477.

269. Clark CW, Johnson JH: **The sounds of the bowhead whale, *Balaena mysticetus*, during the spring migrations of 1979 and 1980**. *Canadian Journal of Zoology* 1984, **62**:1436-1441.

270. Knowlton AR, Clark CW, Kraus SD: **Sounds recorded in the presence of sei whales (*B. borealis*).** *9^th^ Biennial Conference on the Biology of Marine Mammals*: 5^th^-9^th^ December 1991; Chicago. 1991:40.

271. McDonald MA, Hildebrand JA, Wiggins SM, Thiele D, Glasgow D, Moore SE: **Sei whale sounds recorded in the Antarctic.** *Journal of the Acoustical Society of America* 2005, **118**:3941-3945.

272. Cummings WC, Thompson PO, Ha SJ: **Sounds from Bryde’s, *Balaenoptera edeni*, finback, B. physalus, whales in the Gulf of California.** *Fish Bulletin U. S.* 1986, **84**:359-370.

273. Edds P: **Vocalisations of a captive juvenile and free-raning adult-calf pairs of Bryde’s whales, *Balaenoptera edeni*.** *Marine Mammal Science* 1993, **9**: 269-284.

274. Heimlich SL, Mellinger DK, Nieukirk SL, Fox CG: **Types, distribution, and seasonal occurrence of sounds attributed to Bryde’s whales (*Balaenoptera edeni*) recorded in the eastern tropical Pacific, 1999-2001**. *Journal of the Acoustical Society of America* 2005, **118**:1830-1837.

275. Mellinger DK, Clark CW: **Blue whale (*Balaenoptera musculus*) sounds from the North Atlanctic**. *Journal of the Acoustic Society of America* 2003, **114**:1108-1119.

276. Stafford KM, Nicukirk, SL, Fox CG: **Geographic and seasonal variation of blue whale calls in the North Pacific.** *Journal of Cetacean Research and Management* 2001, **3**:65-76.

277. Alling A. Personal Communication to Mellinger DK and Clark CW. In Mellinger DK, Clark CW: **Blue whale (*Balaenoptera musculus*) sounds from the North Atlanctic**. *Journal of the Acoustic Society of America* 2003, **114**:1108-1119.

278. Ljungblad DK, Stafford KM, Shimada H: **Sound attributed to blue whales recorded off the southwest coast of Australia in December 1995**. *Reports of the International Whaling Commission* 1997, **47**: 435-439.

279. Watkins WA, Daher MA, George JE, Rodriguez D: **Twelve years of tracking 52-Hz whale calls from a unique source in the North Pacific.** *Deep-Sea Research I* 2004, **51**:1889-1901.

280. Sirovic A, Hildebrand JA, Wiggins SM, McDonald MA, Moore SE, Thiele D: **Seasonality of blue and fin whales calls and the influence of sea ice in the Western Antarctic Peninsula**. *Deep-Sea Research II* 2004, **51**:2327-2344.

281. Stafford KM: **Acoustic detection and location of blue whales (*Balaenoptera musculus*) from SOSUS by matched filtering.** *Journal of the Acoustical Society of America* 1994, **96**:3250.

282. Berchok CL, Bradley DL, Gabrielson TB: St. **Lawrence blue whale vocalizations revisited: Characterization of calls detected from 1998-2001**. *Journal of the Acoustical Society of America* 2006, **120**:2340-2354.

283. Edds P: **Characteristics of finback, *Balaenoptera physalus*, vocalizations in the St. Lawrence Estuary.** *Bioacoustics* 1988, **1**:131-149.

284. Watkins WA: **The 20 Hz signals of finback whales (*Balaenoptera physalus*).** *Journal of the Acoustical Society of America* 1987, **82**:1901-1912.

285. Thompson PO, Friedl WA: **A long term study of low frequency sounds from several species of whales off Oahu, Hawaii**. *Cetology* 1982, **45:** 1–19.

286. Hafner GW, Hamilton CL, Steiner WW, Thompson TJ, Winn HE: **Signature information in the song of the humpback whale**. *Journal of the Acoustical Society of America* 1979, **66**:1-6.

287. Cummings WC, Thompson PO, Cook R: **Underwater sounds of migrating gray whales, *Eschrichtius glaucus*.** *Journal of the Acoustical Society of America* 1968, **44**:1278-1281.

288. Fish JF, Sumich JL, Lingle GL: **Sounds produced by the gray whale, *Eschrichtius robustus***. *Marine Fisheries Review* 1974, **36**:38-45.

289. Dahlheim ME, Fisher HD, Schempp JD: **Sound production by the gray whale and ambient noise levels in Laguna San Ignacion, Baja California Sur, Mexico**. In: *The Gray Whale* Edited by Jones ML, Swartz L, Leatherwood S. Orlando: Academic Press; 1984: 511-541.

290. Dawbin WH, Cato DH: **Sounds of a pygmy right whale (*Caperea marginata*).** *Marine Mammal Science* 1992, **8**:213:219.

291. May-Collado LJ, Wartzok D: **The freshwater dolphin *Inia geoffrensis geoffrensis* produces high frequency whistles.** *Journal of Acoustical Society of America* 2007, **121**: in press

292. Wang D, Wursig B, Evans WE: **Comparisons of whistles among seven odontocete species.** In: *Sensory Systems of Aquatic Mammals* Edited by Kastelein RA, Thomas JA, and Nachtigal PE. The Netherlands: DeSpil; 1995: 299–323

293. Wang D, Wursig B, Leatherwwod S: **Whistles of boto, *Inia geoffrensis*, and tucuxi, *Sotalia fluviatilis*.** *Journal of the Acoustical Society of America* 2001, **109**: 407–414.

### 294. Diazgranados MC, Trujillo F: Vocal repertoire of the freshwater dolphins Inia geoffrensis and *Sotalia fluviatilis* in Colombia, South America. *Journal of the Acoustical Society of America* 2002, 112: 2400.

295. Wang D, Wang K, Akamatsu T, Fujita F: **Study on whistles of the Chinese River Dolphin or baiji *Lipotes vexillifer*.** *Oceanologia et Limonologia Sinica* 1999, **30**:349–354.

296. Wang X, Wang D, Akamatsu T, Fujita K, Shiraki R: **Estimated detection distance of a baiji’s (Chinese river dolphin, *Lipotes vexillifer*) whistles using a passive acoustic survey method.** *Journal of the Acoustical Society of America* 2006, **120**: 1361-1365.

297. Ford JKB, Fisher HD: **Underwater acoustic signals of the narwhal (*Monodon monocerus*).** *Canadian Journal of Zoology* 1978, **56**: 552-560.

298. Watkins WA, Schevill WE, Ray C: **Underwater sounds of *Monodon* (Narwhal).** *Journal of the Acoustical Society of America* 1970, **49**: 595-599.

299. Shapiro A: **Preliminary evidence for signature vocalizations among free-ranging narwhals (*Monodon monocerus*).** *Journal of the Acoustical Society of America* 2006, **120**: 1695-1705.

300. Karlsen JD, Bisther A, Lydersen C, Haug T, Kovacs KM: **Summer vocalisations of adult male white whales (*Delphinapterus leucas*) in Svalbard, Norway**. *Polar Biology* 2002, **25**:808–817.

301. Sjare BL, Smith TG: **The vocal repertoire of white whales, *Delphinapterus leucas*, summering in Cunningham Inlet, Northwest Territories**. *Canadian Journal of Zoology* 1986, **64**: 407-415.

302. Belikov RA, Bel’kovich VM: **Characteristics of white sea beluga whale (*Delphinapterus leucas* Pall) whistle-like signals**. *XI Session of the Russian Acoustical Society*, November 2001; Moscow, 2001: 19-23.

303. Caldwell MC, Caldwell DK: **Statistical evidence for individual signature whistles in the Pacific whitesided dolphin, *Lagenorhynchus obliquidens***. *Cetology* 1970, **16**:1-21.

304. Yin SE: **Movement patterns, behaviors, and whistle sounds of dolphin groups off Kaikoura, New Zealand**. *M.Sc. Thesis*. Texas A&M University; 1999: 107 pp.

305. Steiner WW: **Species-specific differences in pure tonal whistle vocalizations of five western North Atlantic dolphin species**. *Behavioral Ecology Sociobiology* 1981, **9**:241–246.

306. Moore SE, Ridgway SH: **Whistles produced by common dolphins from Southern California Bight.** *Aquatic Mammals* 1995, **21**:55-63.

307. Oswald JN, Barlow J, Norris TF: **Acoustic identification of nine delphinids species in the eastern tropical Pacific Ocean.** *Marine Mammal Science* 2003, **19**: 20–37.

308. Mullin KD, Higgins LV, Jefferson TA, Hansen LJ: **Sightings of the Clymene dolphin (*Stenella clymene*) in the Gulf of Mexico.** *Marine Mammal Science* 1994, **10** :464–470.

309. Watkins WA, Wartzok D: **Sensory biophysics of marine mammals**. *Marine Mammal Science* 1985, **1**: 219−260.

310. Matthews JN, Rendell LE, Gordon JCD, MacDonald DW: **A review of frequency and time parameters of cetacean tonal calls.** *Bioacoustics* 1999, **10**: 47–71. 21

311. Barzúa-Durán MC, Au WWL: **Whistles of Hawaiian spinner dolphins**. *Journal of the Acoustical Society of America* 2002, **112**: 3064–3072.

312. Barzúa-Durán MC, Au WWL: **Geographic variations in the whistles of spinner dolphins (*Stenella longirostris*) of the Main Hawaiian Islands.** *Journal of the Acoustical Society of America* 2004, **116**: 3757–3769.

313. Driscoll AD: **The whistles of Hawaiian spinner dolphins, *Stenella longirostris***,

*M.Sc. Thesis*, University of California at Santa Cruz; 1995.

314. Boisseau O: **Quantifying the acoustic repertoire of a population: the vocalizations of free-ranging bottlenose dolphins in Fiordland, New Zealand.** *Journal of the Acoustical Society of America* 2005, **117**: 2318-2329.

315. Wang D, Wursig B, Evans WE: **Whistles of bottlenose dolphins: comparisons among populations.** *Aquatic Mammals* 1995, **21**: 65–77.

316. WatkinsWA, Daher MA, Fristrup KM, Notarbartolo di Sciara G: **Fishing and acoustic behavior of Fraser’s dolphin (*Lagenodelphis hosei*) near Dominica, southeast Caribbean**. *Caribbean Journal of Science* 1994, **30**:76–82.

317. Oswald JN, Rankin S, Barlow J: **First description of whistles of Pacific Fraser’s dolphins, *Lagenodelphis hosei***. *Bioacoustics*, submitted.

318. Leatherwood S, Jefferson TA, Norris JC, Stevens WE, Hansen LJ, Mullin KD: **Occurrence and sounds of Fraser’s dolphins (*Lagenodelphis hosei*) in the gulf of Mexico.** *The Texas Journal of Science* 1993, **45**:349-354.

319. Van Parijs SM, Corkeron PJ: **Vocalizations and Behavior of Pacific Humpback dolphins *Sousa chinensis*.** *Ethology* 2001, **107**:701-716.

320. Zbinden K, Pilleri G, Kraus C, Bernath O: **Observations on the behaviour and underwater sounds of the plumbeous dolphin (*Sousa chinensis* G. Cuvier 1829) in the Indus Delta region**. In: *Investigations on Cetacea* Edited by Pilleri G. Germany: University of Bern; 1977: 259-286

321. Schultz KW, Corkeron PJ: **Interspecific differences in whistles produced by inshore dolphins in Moreton Bay, Queensland, Australia.** *Canadian Journal of Zoology* 1994, **72**: 1061–1068

322. Azevedo AF, Van Sluys M: **Whistles of tucuxi dolphins (*Sotalia fluviatilis*) in Brazil: comparisons among populations**. *Journal of the Acoustical Society of America* 2005, **117**: 1456-1464.

323. Azevedo AF, Simão SM: **Whistles produced by marine tucuxi dolphins *Sotalia fluviatilis* in Guanabara Bay, southeastern Brazil**. *Aquatic Mammals* 2002, **28**: 261–266.

324. Erber C, Simão SM: **Analysis of whistles produced by the tucuxi dolphin *Sotalia fluviatilis* from Sepetiba Bay, Brazil**. *Annals of the Brazilian Academy of Sciences* 2004, **76:**381–385.

325. Podos J, da Silva VMF, Rossi-Santos MR: **Vocalizations of Amazon river dolphins, *Inia geoffrensis*: insights into the Evolutionary origins of delphinid whistles**. *Ethology* 2002, **108**: 601–612.

326. Busnell RG, Dziedzic A: **Caracteristiques physiques de certains signaux acoustiques du delphidide *Steno bredanensis*, Lesson**. *Comptes reduns del’Académie des Sciences Paris Series D* 1968, **262**:143-146.

327. Rendell LE, Matthews JN, Gill A, Gordon JCD, MacDonald DW: **Quantitative analysis of tonal calls from five odontocete species, examining interspecific and intraspecific variation**. *Journal of Zoology* 1999, **249**: 403–410.

328. Corkeron PJ, Van Parijs SM: **Vocalizations of eastern Australian Risso’s dolphins, *Grampus griseus****.* *Canadian Journal of Zoology* 2001, **79**: 160–164.

329. Watkins WA, Daher MA, Samuels A, Gannon DP: **Observations of *Peponocephala electra*, the Melon-headed whale, in the southeastern Caribbean**. *Caribbean Journal of Science* 1997, **33**:34-40.

330. Van Parijs SM, Parra GJ, Corkeron PJ: **Sounds produced by Australian Irrawaddy dolphins, *Orcaella brevirostris.*** *Journal of the Acoustical Society of America* 2000, **108**:1938–1940

331. Ford JKB: **Acoustic behavior of resident killer whales (*Orcinus orca*) off Vancouver Island, Bristish Columbia (Canada).** *Canadian Journal of Zoology* 1989, **67**:727-745.

332. Dahlheim ME, Awbrey F: **A classification and comparison of vocalizations of captive killer whales (*Orcinus orca)*.** *Journal of the Acoustical Society of America* 1982, **72**:661–670.

333. Steiner WW, Hain JH, Winn HE, Perkins PJ: **Vocalizations and feeding behavior of the killer whale (*Orcinus orca*).** *Journal of Mammalogy* 1979, **60**:823-827.

334. Thomsen F, Franck D, Ford JKB: **Characteristics of whistles from the acoustic repertoire of resident killer whales (*Orcinus orca*) off Vancouver Island, British Columbia.** *Journal of the Acoustical Society of America* 2001, **109**:1240-1246.

335. Riesch R, Ford JKB, Thomsen F: **Stability and group specificity of stereotyped whistles in resident killer whales, Orcinus orca, off British Columbia**. *Animal Behaviour* 2006, **71**:79-91
