# Supplementary material for: Phylogenetic review of tonal sound production in whales in relation to sociality
Source: BMC Evol Biol. 2007 Aug 10;7:136. doi: 10.1186/1471-2148-7-136 (PMC2000896; doi:10.1186/1471-2148-7-136)
Supplement: Additional file 9 — Association between components of sociality and tonal sound complexity. This table summarizes results from SIMMAP analyses of character associations between social components (selecting the highest social state for polymorphic species) and components of tonal sound complexity on the preferred phylogeny. [file 1471-2148-7-136-S9.doc]

| Tonal Sound Complexity  **Four states** | Group size (p<0.027, p>0.973) | | | |
| --- | --- | --- | --- | --- |
|  | 0 | 1 | 2 | 3 |
| 0 (0-1) *Dij*  *p-value* | 0.079  0.91 | 0.0090  0.86 | -0.028  0.001* |  |
| 1 (1.1-2) *Dij*  *p-value* | -0.036  0.005* | 0.0023  0.79 | 0.123  0.92 |  |
| 2 (2.1-3) *Dij*  *p-value* | -0.0049  0.015* | 0.052  0.90 | -0.022  0.007* |  |
| 3 (>3.1) *Dij*  *p-value* | -0.0051  0.021* | 0.026  0.89 | -0.0025  0.013* |  |
| Tonal Sound Complexity  **Four states** | Group associations/stability (p<0.027, p>0.973) | | | |
|  | 0 | 1 | 2 | 3 |
| 0 (0-1) *Dij*  *p-value* | 0.080  0.93 | 0.0098  0.88 | -0.031  p<0.0001* | 0.0012  0.84 |
| 1 (1.1-2) *Dij*  *p-value* | -0.036  0.005* | 0.023  0.89 | 0.061  0.91 | 0.042  0.89 |
| 2 (2.1-3) *Dij*  *p-value* | -0.062  0.009* | 0.014  0.92 | 0.023  0.92 | -0.054  0.010* |
| 3 (>3.1) *Dij*  *p-value* | -0.0057  0.009* | 0.020  0.94 | 0.0054  0.87 | -0.00069  0.027* |
| Tonal Sound Complexity  **Four states** | Group Composition (p<0.027, p>0.973) | | | |
|  | 0 | 1 | 2 | 3 |
| 0 (0-1) *Dij*  *p-value* | 0.087  0.94 | -0.024  0.004* | -0.0069  0.021* | 0.0049  0.86 |
| 1 (1.1-2) *Dij*  *p-value* | -0.017  0.004* | 0.075  0.93 | 0.031  0.91 | -0.0007  0.04 |
| 2 (2.1-3) *Dij*  *p-value* | -0.0014  0.033 | -0.0043  0.021* | 0.029  0.91 | 0.003  0.84 |
| 3 (>3.1) *Dij*  *p-value* | 0.0089  0.84 | 0.014  0.90 | -0.0016  0.015* | 0.0055  0.56 |

*Significant negative associations, **significant positive associations

D=0.394, p<0.0001, n_p-value_=510, n_D_=2000 Group Size and Tonal Sound Complexity

D=0.364 p<0.0001, n_p-value_=553, n_D_=2000 Group Association/Stability and Tonal Sound Complexity

D= 0.306 p<0.0001 , n_p-value_=832, n_D_=2000 Group Composition and Tonal Sound Complexity
