# Supplementary material for: Phylogenetic review of tonal sound production in whales in relation to sociality
Source: BMC Evol Biol. 2007 Aug 10;7:136. doi: 10.1186/1471-2148-7-136 (PMC2000896; doi:10.1186/1471-2148-7-136)
Supplement: Additional file 10 — Association between sociality and tonal sound complexity. This table summarizes results from SIMMAP analyses of character associations between social structure (categorized as 1–4) and tonal sound complexity on the preferred phylogeny across reference phylogenies (see Methods). [file 1471-2148-7-136-S10.doc]

| Tonal Sound Complexity  **Four states**  D=0.375 p<0.001* (2states)  D=0.356 p<0.001* (4states) | Social Structure (p<0.027, p>0.973)  **Arnasson *et al.* 2004 filtered (n=325)** | | | |
| --- | --- | --- | --- | --- |
|  | 0 | 1 | 2 | 3 |
| 0 (≤ 1 mean inflection point)  *D-statistic*  *p-value* | 0.081  0.73 | 0.053  0.75 | -0.041  0.003* | -0.0052  0.006* |
| 1 (≤ 1 mean inflection point)  *D-statistic*  *p-value* | -0.044  p<0.001* | 0.002  0.95 | 0.111  0.99** | 0.036  0.98** |
| 0 (0-1) *Dij*  *p-value* | 0.082  0.94 | 0.016  0.84 | -0.033  p<0.001* | 0.0094  0.90 |
| 1 (1.1-2) *Dij*  *p-value* | -0.037  0.006* | 0.026  0.91 | 0.077  0.95 | 0.022  0.93 |
| 2 (2.1-3) *Dij*  *p-value* | -0.0036  0.009* | 0.010  0.87 | 0.020  0.92 | -0.0024  0.006* |
| 3 (>3.1) *Dij*  *p-value* | -0.0045  0.006* | 0.016  0.88 | 0.005  0.84 | 0.0025  0.86 |
| Tonal Sound Complexity  **Four states**  D=0.382 p<0.001* (2states)  D=0.358 p<0.001* (4states) | **Nikaido *et al.* 2001 filtered (n=341)** | | | |
|  | 0 | 1 | 2 | 3 |
| 0 (≤ 1 mean inflection point)  *D-statistic*  *p-value* | 0.083  0.75 | 0.053  0.72 | -0.043  p<0.001* | -0.0047  0.003* |
| 1 (≤ 1 mean inflection point)  *D-statistic*  *p-value* | -0.046  p<0.001* | 0.0004  0.91 | 0.1156  0.99** | 0.036  0.99** |
| 0 (0-1) *Dij*  *p-value* | 0.082  0.91 | 0.00006  0.74 | -0.033  0.005* | 0.0090  0.85 |
| 1 (1.1-2) *Dij*  *p-value* | -0.037  p<0.001* | 0.0234  0.91 | 0.0776  0.92 | 0.0235  0.92 |
| 2 (2.1-3) *Dij*  *p-value* | -0.0044  0.005* | 0.0133  0.92 | 0.0223  0.87 | -0.0032  0.020* |
| 3 (>3.1) *Dij*  *p-value* | -0.0050  0.011* | 0.0165  0.93 | 0.0054  0.89 | 0.0026  0.90 |
| Tonal Sound Complexity  **Four states**  D=0.442/0.269 p<0.001* (2states)  D=0.360/0.217 p<0.001* (4states) | **Messenger and McGuire 1998 (n=4) filtered/**  **Bayesian (n=2001)** | | | |
|  | 0 | 1 | 2 | 3 |
| 0 (≤ 1 mean inflection point)  *D-statistic*  *p-value* | 0.085/0.072  p>0.999**/0.86 | 0.064/0.016  0.5/0.65 | -0.050/-0.027  p<0.001*/0.004* | -0.015/-0.007  p<0.001*/0.025* |
| 1 (≤ 1 mean inflection point)  *D-statistic*  *p-value* | -0.044/-0.053  p<0.001*/0.004* | -0.015/0.015  p<0.001*/0.96 | 0.12/0.048  p>0.999**/0.98** | 0.05/0.031  p>0.999**/0.95 |
| 0 (0-1) *Dij*  *p-value* | 0.081/0.052  p>0.999**/0.94 | 0.0062/0.013  0.75/0.85 | -0.032/-0.014  p<0.001*/0.04 | -0.026/-0.009  p<0.001*/0.06 |
| 1 (1.1-2) *Dij*  *p-value* | -0.036/-0.033  p<0.001*/0.021* | 0.023/0.0007  p>0.999**/0.81 | 0.072/0.037  p>0.999**/0.93 | 0.038/0.025  p>0.999**/0.92 |
| 2 (2.1-3) *Dij*  *p-value* | -0.0041/0.0011  p<0.001*/0.78 | 0.0060/0.012  0.75/0.87 | 0.026/-0.0032  0.75/0.064 | -0.0032/0.003  p<0.001*/0.81 |
| 3 (>3.1) *Dij*  *p-value* | -0.0057/-0.0019  p<0.001*/0.09 | 0.018/0.006  0.5/0.85 | 0.0028/0.0015  0.75/0.77 | 0.0037/0.005  p>0.999**/0.87 |
| Tonal Sound Complexity  **Four states**  D=0.378 p<0.001* (2states)  D=0.355 p<0.001* (4states) | **May-Collado et al. 2007 filtered (n=1069)** | | | |
|  | 0 | 1 | 2 | 3 |
| 0 (≤ 1 mean inflection point)  *D-statistic*  *p-value* | 0.082  0.74 | 0.053  0.73 | -0.041  0.007* | -0.006  p<0.001* |
| 1 (≤ 1 mean inflection point)  *D-statistic*  *p-value* | -0.045  p<0.001* | 0.0012  0.92 | 0.112  0.99** | 0.037  0.97** |
| 0 (0-1) *Dij*  *p-value* | 0.082  0.92 | 0.0018  0.83 | -0.033  p<0.001* | 0.009  0.89 |
| 1 (1.1-2) *Dij*  *p-value* | -0.037  0.0009* | 0.025  0.93 | 0.077  0.93 | 0.023  0.90 |
| 2 (2.1-3) *Dij*  *p-value* | -0.0037  0.021* | 0.013  0.91 | 0.020  0.90 | -0.003  0.012* |
| 3 (>3.1) *Dij*  *p-value* | -0.0043  0.013* | 0.014  0.89 | 0.007  0.89 | 0.002  0.84 |

*Significant negative associations, **significant positive associations
