# Supplementary material for: Phylogenetic review of tonal sound production in whales in relation to sociality
Source: BMC Evol Biol. 2007 Aug 10;7:136. doi: 10.1186/1471-2148-7-136 (PMC2000896; doi:10.1186/1471-2148-7-136)
Supplement: Additional file 11 — Regression between group size and tonal sound characteristics. This table summarizes results from PDAP regression between group size and mean minimum frequency (MMinF) and mean number of inflection points (IP) across reference phylogenies (see Methods). [file 1471-2148-7-136-S11.doc]

*Significant results

| Acoustic Parameters vs  Group Size | May-Agnarsson  2000 trees burnin | May-Collado  filtered | Messenger and McGuire (1998)  filtered | Messenger and McGuire (1998)  Parsimony on their nuclear/morphology data | Messenger and McGuire (1998)  Bayesian on their nuclear/morphology data | Arnasson et al (2003)  Filtered | Nikaido et al. (2001)  filtered |
| --- | --- | --- | --- | --- | --- | --- | --- |
| All Cetaceans | | | | | | | |
| MMinF  R-square  Df  p-value | 3.5  29  0.31 | 3.2  29  0.33 | 1.9  29  0.24 | 2.8  21  0.44 | 2.9  21  0.43 | 3.7  29  0.30 | 3.1  29  0.17 |
| IP  R-square  Df  p-value | 7.5  33  0.05* | 7.4  33  0.05* | 10.2  33  0.03* | <1  22  0.86 | <1  22  0.85 | 5.1  33  0.09 | 3.7  33  0.13 |
| Toothed-Whales | | | | | | | |
| MMinF  R-square  Df  p-value | 13.8  23  0.03* | 13.2  23  0.03* | 5.8  23  0.12 | 11.4  16  0.08 | 9.7  16  0.21 | 15.4  23  0.03* | 13.5  23  0.03* |
| IP  R-square  Df  p-value | 7.3  24  0.09 | 7.1  24  0.09 | 10  24  0.06 | <1  17  0.92 | <1  17  0.93 | 4.7  24  0.14 | 3.5  24  0.17 |
