# Supplementary material for: Phylogenetic review of tonal sound production in whales in relation to sociality
Source: BMC Evol Biol. 2007 Aug 10;7:136. doi: 10.1186/1471-2148-7-136 (PMC2000896; doi:10.1186/1471-2148-7-136)
Supplement: Additional file 12 — Regression between duration and other acoustic variables. This table summarizes results from PDAP regression analyses between duration (s) and absolute (AbsMinF) and mean minimum (MMin) frequency and mean number of inflection points (IP) across reference phylogenies (see Methods). [file 1471-2148-7-136-S12.doc]

| Acoustic Parameters vs  Group Size | May-Agnarsson  2000 trees burnin | May-Collado  filtered | Messenger and McGuire (1998)  filtered | Messenger and McGuire (1998)  Parsimony on their nuclear/morphology data | Messenger and McGuire (1998)  Bayesian on their nuclear/morphology data | Arnasson et al (2003)  Filtered | Nikaido et al. (2001)  filtered |
| --- | --- | --- | --- | --- | --- | --- | --- |
| All Cetaceans | | | | | | | |
| Abs MinF  R-square  Df  p-value | 17  31  0.017 | 16.5  31  0.018 | 19.4  31  0.01 | 29.7  20  0.008 | 37  20  0.002 | 16.5  31  0.02 | 16.6  31  0.018 |
| MMinF  R-square  Df  p-value | 17.5  29  0.019 | 19  29  0.014 | 12.5  29  0.05 | Not significant | Not significant | 19  29  0.014 | 19  29  0.014 |
| IP  R-square  Df  p-value | 11.9  32  0.05 | 11.7  32  0.04 | 9.4  32  0.07 | 35.6  21  0.002 | 42.3  20  0.001 | 11.9  32  0.04 | 12.2  32  0.04 |
| Toothed-Whales | | | | | | | |
| Abs MinF  R-square  Df  p-value | 37.8  22  0.001 | 39  22  0.001 | 38.7  22  0.001 | 43.9  15  0.003 | 45.9  15  0.002 | 39.2  22  0.001 | 38.1  22  0.001 |
| MMinF  R-square  Df  p-value | 23.8  23  0.013 | 26.7  23  0.008 | 24.6  23  0.01 | 25.8  16  0.03 | 23.9  16  0.03 | 26.4  23  0.008 | 26  23  0.009 |
| IP  R-square  Df  p-value | 44.8  23  p<0.001 | 44.1  23  P<0.001 | 44.2  23  p<0.001 | 38.3  16  0.006 | 46  15  0.002 | 45  23  p<0.001 | 46.1  23  p<0.001 |
